# Immunological Study of Reconstructed Common Ancestral Sequence of Adenovirus Hexon Protein

**DOI:** 10.3389/fmicb.2021.717047

**Published:** 2021-10-27

**Authors:** Yingchen Wang, Zhe Zhang, Lei Shang, Hong Gao, Xiqiao Du, Falong Li, Ya Gao, Guiyun Qi, Weiyuan Guo, Zhangyi Qu, Tuo Dong

**Affiliations:** ^1^Department of Microbiology, Public Health College, Harbin Medical University, Harbin, China; ^2^Harbin Center for Disease Control and Prevention, Harbin, China; ^3^The Second Affiliated Hospital, Harbin Medical University, Harbin, China; ^4^Department of Natural Focus Disease Control, Institute of Environment-Associated Disease, Sino-Russia Joint Medical Research Center, Harbin Medical University, Harbin, China

**Keywords:** human adenovirus, protein structure, evolutionary trace, common ancestor reconstruction, serum immunity

## Abstract

**Aim:** To reconstruct the ancestral sequence of human adenoviral hexon protein by combining sequence variations and structural information. And to provide a candidate hexon protein for developing new adenoviral vector capable of escaping the pre-existing immunity in healthy populations.

**Methods:** The sequences of 74 adenovirus-type strains were used to predict the ancestral sequence of human adenovirus hexon protein using FastML and MEGA software. The three-dimensional structure model was built using homology modeling methods. The immunological features of ancestral loop 1 and loop 2 regions of sequences were tested using protein segments expressed in a prokaryotic expression system and polypeptides synthesized with human serum samples.

**Results:** The tower region of the hexon protein had the highest sequence variability, while the neck and base regions remained constant among different types. The modern strains successfully predicted the common ancestral sequence of the human adenovirus hexon. The positive sera against neutralizing epitopes on the common ancestor of adenoviral hexon were relatively rare among healthy adults.

**Conclusion:** The existing strains inferred the common ancestor of human adenoviruses, with epitopes never observed in the current human strains. The predicted common ancestor hexon is a good prospect in the improvement of adenovirus vectors.

## Introduction

Adenovirus is among the most important model organisms in medical research. In healthy adults, adenoviruses cause transient or mild infections, making adenoviruses good candidates for gene therapy, immunotherapy, or immunization (Khanal et al., 2018). Adenoviral vectors are orthodox in vaccine research and development, gene therapy, oncolytic biotherapy, and genetic engineering (Wold and Toth, 2013). However, the pre-existing adenovirus immunity in healthy populations limits the application of adenoviral vectors, including HAdv5 (Mast et al., 2010). Therefore, re-designing the adenoviral structural proteins will improve the vectors and probably escape pre-existing immunity.

The adenovirus forms an icosahedral nucleocapsid with 252 capsids, including 240 hexons and 12 pentons, and each penton is connected with one or two fibers. Protein IIIa, protein VIII, protein IX, and other small-molecular cement proteins are located between the penton and the hexon to help maintain capsid stability (Liu et al., 2010; Reddy et al., 2010; Reddy and Nemerow, 2014). The X-ray diffraction determined the three-dimensional structures of the three major structural adenoviral proteins; hexon, penton, and fiber. The adenoviral hexon is a homotrimeric protein interspersed and surrounded by three hexon monomers forming a complete hexon (Pichla-Gollon et al., 2007). The hexon is divided into three regions from the bottom to the top: the base, neck, and tower with three minor towers. Two adjacent hexon monomers entangle each tower. The hexon base area is β sheets and α helices, while the tower region consists of random coils and corners. The currently known neutralizing epitopes are located in the adenoviral hexon. When penetrating the host cell membrane, this ligand binds to the host cell dynein (Scherer and Vallee, 2015). The human adenoviral penton is a homopentamer with five monomers, divided into a base and a top. The base has folds and alpha-helices, and the top has irregular coils (Szolajska et al., 2012). The adenoviral fiber is a homotrimer composed of three monomers (van Raaij et al., 1999), and the monomer N-terminus and mid-section have random coils and are twisted into a shaft of the fiber. The C-terminus has two sets of anti-parallel β sheets attached to form a fiber knob. The fiber knob is the ligand that binds adenovirus capsid to the host cell receptors (Persson et al., 2009).

Hexon protein is the main neutralizing antigen of human adenovirus. Anti-hexon antibody protects host cells by preventing adenovirus from invading host cells (Crawford-Miksza and Schnurr, 1996). Hexon proteins are type-specific, and so, replacing type 5 adenovirus hexon with type 2 converted the neutralizing antigen to type two (Gall et al., 1998). Another study showed that the neutralizing epitopes of human adenovirus type 3 are linear epitopes located in the five exposed hypervariable loop regions on the hexon tower region (Yuan et al., 2009). Replacing all the five hypervariable loop regions of human type 7 adenovirus with the corresponding amino acid sequence of human type 3 adenovirus converts the neutralizing antigen of the chimeric virus to type 3 (Tian et al., 2011). Monoclonal antibodies against adenovirus type 7 can be isolated from mice immunized with chimeric adenoviral strains (Liu et al., 2014).

The pre-existing immunity in the healthy population limits the clinical application of adenoviral vectors (Alonso-Padilla et al., 2016). However, adenoviral vectors are traditional in gene therapy and vaccine research and development (Zhang and Zhou, 2016). Human adenovirus types 2, 3, 5, and 7 are adenoviral types frequently reported (Gray et al., 2007; Mandelboim et al., 2011; Alharbi et al., 2012). Serological studies depicted that these viruses have high levels of neutralizing antibodies in healthy adults (Mennechet et al., 2019). For example, the positive rate of serum neutralizing antibodies against human adenovirus type 5 in the adult population in Guangzhou, China, is 73.1% (Zhang et al., 2013). The sera positive rates for human adenovirus type 3 and type 7 were 38.13 and 18.71% respectively (Tian et al., 2020). High levels of serum neutralizing antibodies against adenovirus type 5 in natural populations failed a clinical trial of an adenoviral HIV vaccine (Ondondo, 2014).

The COVID-19 vaccines based on adenoviral vectors were approved for marketing and have achieved good results (Pushparajah et al., 2021). The COVID vaccine designed by the Russian research team involves two vaccinations using two adenoviral vectors, type 5 and 26, respectively, for effective immunity (Logunov et al., 2021). The Convidecia^(*TM*)^ COVID vaccine produced by the Chinese CanSinoBio is based on human adenovirus type 5 vectors. Clinical trials showed that the pre-existing immunity in the population affects the Convidecia^(*TM*)^ vaccine. After vaccination, the anti-coronavirus antibodies produced by the high-level pre-existing immunity group (anti-adenovirus titer greater than 1:200) were half lower than the control group (Zhu et al., 2020). The Oxford-AstraZeneca used the chimpanzee adenoviral vector type 68 to produce the ChAdOx1^(*TM*)^ vaccine. Antibodies against simian adenovirus type 68 are rare in the human population. Humans vaccinated with the ChAdOx1^(*TM*)^ vaccine had high-titer anti-simian adenovirus type 68 antibodies, and the antibodies are likely to persist in the human serum (Ramasamy et al., 2021). Therefore, the adverse effects of anti-vector immunity must be considered when developing adenoviral vector vaccines. Nevertheless, these findings also imply that a novel adenoviral vector with altered neutralizing epitopes would escape the pre-existing immunity.

Molecular evolution theory is successfully applied in protein engineering. Several proteins and viruses have been successfully engineered from predicted common ancestor sequences (Spence et al., 2021). Gonzalez et al. (2014) analyzed the molecular evolution of hydrophobic phosphate-binding proteins expressed by humans and different animals and found that anti-HIV activity significantly improved the solubility of the ancestral protein in *Escherichia coli*. By molecular evolution analysis, Doria-Rose et al. (2005) predicted the common ancestral sequence of the type 1 HIV subtype B membrane protein gene. The constructed ancestral membrane protein had complete biological functions, such as the host cell CCR5 receptor in the eukaryotic system. Gullberg et al. (2010) compared the surface antigen VP1 protein sequence of different Coxsackievirus group B type 5 strains, inferred their common ancestor sequence, and replaced the VP1 coding sequence in a modern train with the synthesized ancestral sequence. The resulting chimeric virus had complete biological functions, such as infectivity (Gullberg et al., 2010). Elsewhere, inferring the common ancestor sequence and artificially synthesizing the corresponding sequence effectively modified the adeno-associated virus vector (Santiago-Ortiz et al., 2015). The tissue tropism of the modified adeno-associated virus vector widened (Zinn et al., 2015). Adenovirus and adeno-associated virus (AAV) are two different viruses. The AAV is applicable in gene therapy vectors, but virus vectors only carry small (<5 kbp) external gene fragments. However, the gutless adenoviral vectors carry up to 30 kbp of foreign fragments (Crystal, 2014).

The hexon protein is the most important adenoviruses neutralizing antigen. Therefore, constructing the common ancestor sequence of human adenovirus hexon facilitates designing a novel hexon serotype with an unchanged skeleton. The common ancestral hexon protein may have a lower pre-existing immune response in the human population.

## Materials and Methods

### Bio Sequence and Structural Data Source

Adenoviral genome sequences were downloaded from the GenBank database. Protein structures of adenoviral proteins were obtained from the PDB (RCSB) database. The accession numbers of sequences and structures used in this study are summarized in the Supplementary File.

### Antigen and Serum Preparation

The coding sequences of antigen protein segments were cloned into PET-28a plasmids and expressed in *E. coli* BL21. Expressed protein segments were purified and prepared by nickel affinity chromatography. The antigen peptide was artificially synthesized and conjugated to the keyhole limpet hemocyanin (KLH) by GL BioChem Ltd., (Shanghai, China). New Zealand white rabbits were inoculated twice with each KLH-conjugated peptide. Positive control rabbit sera were collected 28 days after the last inoculation. Negative control sera were collected from healthy rabbits.

Human serum samples were collected from healthy volunteers visiting the physical examination center at the 2nd Affiliated Hospital of Harbin Medical University. All volunteers were above 18 years old. Each enrolled volunteer signed a written informed consent form. The experimental design in this report met the requirements of the Helsinki declaration. The Ethics Committee of Harbin Medical University approved the research scheme presented in this report.

### Molecular Evolution Analysis of Adenoviruses

#### Homology Modeling of Adenoviral Hexon

The three-dimensional structure models of the hexon proteins were built using the Swiss-Model online server (https://swissmodel.expasy.org), with human adenovirus types 3 (strain GB, GenBank AY599834) and 7 (strain Gomen, GenBank AY594255) (Waterhouse et al., 2018), respectively. The hexon structure of adenovirus type 5 was adopted from the X-Ray resolved structures in the PDB (RCSB) database (accession numbers 1P30 and 3TG7) (Rux et al., 2003). The Modeller package (Fiser et al., 2000) repaired the loop regions missing 1P30 and 3TG7. The reference structure in evolutionary trace projection was the hexon model of GB strain because this model had known epitopes (Yuan et al., 2009). The models were optimized using the conjugate gradient and the steepest descent methods, respectively (Kini and Evans, 1991). The Verifi-3D algorithm evaluated the model reliability (Yuan et al., 2012).

#### Evolutionary Analysis of Adenoviral Genome

The complete adenovirus genome sequence data was screened from the GenBank to understand the evolutionary characteristics of the adenovirus genome. The artificially constructed strains and strains with incorrect annotations were eliminated. Qualified full sequences were selected to construct a reference dataset. The amino acid sequence of each encoded product was extracted according to the genome annotation, and a protein sequence data subset was constructed. The adenovirus genome sequences were aligned using the Clustal Omega software (Sievers et al., 2011), and the results were edited using the BioEdit software (version 7.09). The genomic and phylogenetic tree of the hexon was constructed using the MEGA software (version 10.0.1). Bootstraps of 1,000 replications tested the robustness of evolutionary trees. The bootstrap values are shown percentages above phylogenetic tree internal nodes.

The amino acid sequence homology at each residue position was counted based on the alignment results of the adenovirus hexon sequence dataset T74. The following formula was used:


Homogeneity=Frequency⁢of⁢the⁢most⁢abundant⁢amino⁢acid⁢residue⁢on⁢each⁢column/number⁢of⁢sequences

The variability index was calculated based on the homogeneity using the formula:


Variability⁢index=1-Homogeneity

The variability index was projected to the hexon structure model of the GB strain by the PyMol software using an in-house script. The variability results of each site are divided into five categories, shown by the five colors. Gray represents the completely conserved residues, blue represents residues with 0–0.2 variability, green represents residues with 0.2–0.4 variability, yellow represents residues with 0.4–0.6 variability, and red represents residues with variability larger than 0.6.

### The Common Ancestor Sequence of Human Adenoviral Hexon

#### Prediction of the Ancestral Sequence to Adenoviral Hexon

The common ancestor sequence of human adenovirus hexon was predicted using FastML (version 3.15) software (Ashkenazy et al., 2012) based on the sequence alignment and constructed phylogenetic tree. A stratified sampling method extracted four subsets from the original dataset of adenovirus hexon sequences to test the robustness of the constructed ancestral sequence. The subsets are the classical type data (containing 74 types of human adenovirus), classical species data (including four species and 23 types of human adenovirus), 95% homology, and 90% homology data. The CD-HIT software, widely used for redundancy analysis of large databases, prepared the 90% and 95% homology data (Fu et al., 2012). For example, using the 90% homology data, the preparing process followed this series: first, all sequences were divided into several groups based on the homology of the pairwise alignment. The cut-off homogeneity within each group was 90%. The cut-off homogeneity between groups < 90%, and the representative sequence of each group was the central sequence. The representative sequences of all groups aggregated into a 90% homology dataset. The common ancestor sequence was constructed from the four subsets mentioned above, and the influence of data screening criteria on the construction of the common ancestor sequence was analyzed. The Rate4Site software measured the credibility of different amino acid positions in each common ancestor sequence (Mayrose et al., 2004).

#### Prediction of the Three-Dimensional Structure of the Adenovirus Hexon Common Ancestor

Homology modeling of hexon proteins was performed using the SWISS-MODEL online server with the predicted common ancestor sequence as the target protein (Burley et al., 2019). The QSQE module is the core algorithm for the SWISS-Model server for constructing homology models (Waterhouse et al., 2018). The algorithm is optimized for improved homologous and heterologous multimeric protein structure modeling (Bertoni et al., 2017). The qualitative model energy analysis method (QMEAN) measured successfully constructed protein models (Benkert et al., 2011). The modeling results in the PDB file format were visualized *via* PyMol and RosMol software, and the side, top, and bottom views of the structural model were drawn.

### Antigenicity of the Ancestral Hexon

#### Experiment Design

Based on the predicted common ancestral sequence and structure of human adenovirus hexon, the target for measuring antigenicity was the linear neutralizing epitope in the second loop region of the hexon. The purpose of the immunoassay is to compare the antigenic difference between the hypervariable and conserved regions of the hexon from ancestral and modern strains. Although the ancestral sequence was predicted using 74 adenoviral types, it was not necessary to test the ancestral sequence against all 74 strains in serum assays. Three modern strains were selected as the cross reference, including human adenovirus type 3, 5, and 7. The HAdv-3 was selected for it had been dominating strains in Harbin city in recent years (Wang et al., 2018). The HAdv-5 was selected for it had been widely utilized as adenoviral vectors (Zhang and Zhou, 2016). The HAdv-7 was selected for it was the most abundant type within 959 complete genome sequences of human adenovirus as illustrated in the Supplementary Table 1.

The neutralizing epitope polypeptides were synthesized for the common ancestor of human adenovirus, human adenovirus types 3, 5, and 7, and universal antigen polypeptides from the conserved region of human adenovirus, respectively. The coding sequences of the loop 1 region of the ancestral sequence and current strains were cloned into PET-28a plasmids and expressed in *E. coli* BL21. Expressed protein segments were purified and prepared by nickel affinity chromatography. The positive control sera were obtained from rabbits immunized with antigen polypeptides or proteins, and the negative control from healthy rabbits. These rabbit sera were used to estimate the proportion of positive samples against these adenoviral epitopes in healthy human volunteers.

#### Peptide ELISA Assays

The antigen polypeptide solution was diluted to 20 μg/ml with ELISA coating solution, and 100 μl was added per well on the ELISA plate and incubated overnight at 4°C. The coating solution was discarded, the plate was washed thrice with PBST. To each well, 200 μl of 5% BSA blocking solution was added and incubated for 2 h at 37°C. Incubated plates were washed thrice with PBST. The human serum for testing and the control serum (rabbit serum) were diluted to a 1:200 final ratio, including positive and negative control sera. To each well, 100 μl of respective samples for testing were added. The samples included: human serum, control rabbit serum, and PBST. The plates containing samples were incubated at 37°C for 1 h and washed thrice with PBST. To each well, 100 μl of HRP-labeled secondary antibody (goat anti-human or goat anti-rabbit antibody) diluted at 1:8,000 was added, and the plate was incubated at 37°C for 1 h. After washing three times with PBST, 100 μl of substrate TMB solution was added to each well. Color development took 15 min, after which 50 μl of the color-stopping solution was added per well to stop color development, and the A450 absorption value was measured using the microplate reader Tecan(TM) Infinite 200 Pro.

#### Protein ELISA Assay

Purified antigen protein segments containing the loop 1 region of the ancestral sequence, human adenovirus type 3, type 5, and type 7 were prepared separately. Each protein segment was diluted to 20 μg/ml with ELISA coating solution, and 100 μl of diluted proteins were added per ELISA plate well. The plate was incubated overnight at 4°C. The rest of the protocol was as described in the peptide ELISA assay above.

### Statistics

Measurement data between groups and within groups were analyzed using ANOVA and paired *t* test, chi-square test with count data, grade data using Kruskal–Wallis test analysis. All statistical analysis was utilized with R Language (version 4.0.5).

## Results

### Molecular Evolution Analysis of Human Adenovirus

By August 21st 2021, there were 1,327 adenoviral genome sequences published in the GenBank database. A total of 353 adenovirus strains were obtained and included in the dataset Gnm353 after removing artificially engineered, constructed adenoviral strains, and strains with redundant sequences. The Gnm353 data has 271 complete genome sequences of human adenovirus, including seven human adenovirus species with 74 types. The dataset HAdvGnm959 containing all the 959 human adenovirus strains was also prepared to summarize the human adenovirus species and types. The sequences were downloaded in FASTA format before alignment and manually checked for errors.

#### Homogeneity of Genes in Human Adenoviral Genome

The homogeneity for each column was calculated based on the whole genome sequence alignment of Gnm353. The AY599834 (human adenovirus type 3 GB strain) encoded 44 products whose coding regions were summarized separately (Table 1). The coding regions of the nucleic acid sequences corresponding to most of the human adenovirus proteins are highly conserved (Table 1). Seventeen of the 44 products have >80% average homology, and 20 products have 70–80% average homology while the rest are below 70%. The IVa2 protein has the highest homology among the products with known functions. The IVa2 coding region of the nucleic acid sequence has 88.77% homology. The IVa2 protein hydrolyzes host cell ATP during the virus assembly and energizes the assembly process. The fiber protein had the lowest homology (65.06%) in the coding region of the nucleic acid sequence. Among the major antigens on the adenoviral surface, hexon had the highest homology (84.11%), followed by penton (82.44%) and fiber (65.06%).

**TABLE 1:** Homogeneity of coding sequences from adenovirus.

Gene	Product	Homogeneity	Gene	Product	Homogeneity
E1A	29.1 kDa Protein	72.62%	L4	Hexon associated Protein	78.47%
E1A	25 kDa Protein	72.04%	L4	33 kDa Protein	72.26%
E1A	6 kDa Protein	72.34%	L4	22 kDa Protein	69.35%
EIB	Small T antigen	77.27%	L4	Protein VIII	87.15%
EIB	Large T antigen	78.59%	E3	12.5 kDa Glycoprotein	82.08%
IX	Protein IX	75.45%	E3	16 kDa Protein	65.79%
IVa2	IVa2 Protein	88.77%	E3	18.5 kDa Glycoprotein	66.18%
E2B	DNA Polymerase	86.60%	E3	20.1 kDa Protein	60.37%
E2B	20.6 kDa Protein	88.82%	E3	20.1 kDa Protein	55.81%
E2B	19 kDa Protein	88.09%	E3	9 kDa Glycoprotein	29.62%
E2B	DNA binding protein	79.88%	E3	10.3 kDa Protein	77.57%
E2B	Terminal Protein	86.33%	E3	14.9 kDa Protein	70.96%
E2B	9.7 kDa Protein	85.72%	E3	14 kDa Protein	79.02%
L1	55 kDa Protein	83.69%	U	U Protein	81.38%
L1	Protein IIIa	83.84%	L5	Fiber	65.06%
L2	Penton	82.44%	E4	Orf6/7	74.18%
L2	Protein VII	83.11%	E4	33.2 kDa Protein	79.23%
L2	Protein V	78.45%	E4	13.6 kDa Protein	73.36%
L3	Protein VI	80.97%	L5	Unknown Protein	78.69%
L3	Hexon	84.11%	E4	11 kDa Protein	81.19%
L3	23 kDa Proteinase	85.47%	E4	14.3 kDa Protein	72.74%
E2A	DNA binding Protein	78.00%	E4	13.9 kDa Protein	74.32%

#### Construction of the Evolutionary Tree of Human Adenovirus Hexon

**FIGURE 1:**
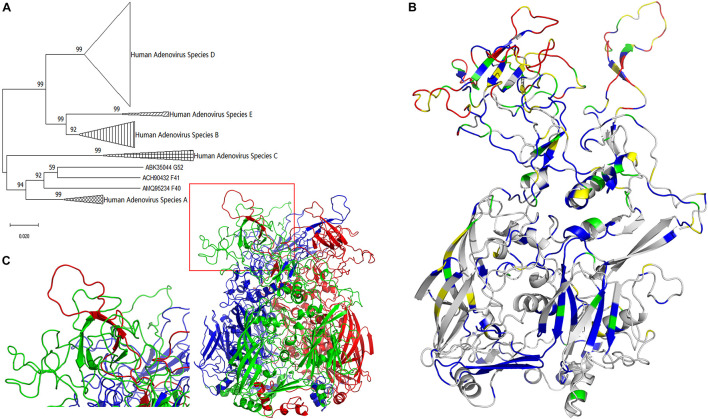
Evolutionary trace of human adenovirus hexon. **(A)** The neighbor-joining tree of adenovirus was based on hexon protein amino acid sequences. The bootstrap values in percentages were shown above branches. **(B)** The evolutionary trace was projected to a monomer of hexon protein. Gray represents the completely conserved residues, blue represents residues with 0–0.2 variability, green represents residues with 0.2–0.4 variability, yellow represents residues with 0.4–0.6 variability, and red represents residues with variability larger than 0.6. The conservative gray and blue sites with lower variability are concentrated at the bottom, and the highly variable red and yellow spots are concentrated on the top. **(C)** The three-dimensional structure model of the human adenovirus hexon was drawn with the ribbon view. The adenoviral hexon was a homotrimer including three monomers colored in red, green and blue respectively. The one tower region surrounded by a red box was magnified to the lower left corner, showing this tower region was composed of an L1 Loop (in green) and an L2 Loop (in red).

Therefore, it is rational to conclude there is a common ancestral sequence to adenoviral hexon proteins. The molecular evolutionary relationship of adenovirus hexon requires further clarification to predict the common ancestor sequence. Based on the results of genome evolution analysis, hexon sequences from 74 types were extracted as a new dataset and named T74. Sequences were aligned to the T74 dataset, and the alignment results were manually checked for errors. Alignment errors arise from erroneous positioning of single indel events and their effect (Landan and Graur, 2009). The phylogenetic tree of human adenovirus hexon based on JTT (Jones-Taylor-Thornton) distances (Jones et al., 1992) was constructed by the neighbor-joining method (Figure 1A).

The molecular evolutionary relationship of the human adenovirus hexon reflects the difference between the species and types of human adenoviruses, with 100% bootstraps of the species (serogroup) (Figure 1A). The first-level clades of subgroups had over 70% bootstrap, justifying the phylogenetic tree construction method adopted in this study. However, species F and G of the hexon protein merged into one clade with 92% bootstrap value, probably because both species (F and G) are transmitted through the gastrointestinal tract.

#### Homology Modeling of Human Adenovirus Hexon

The hexon structure models of the human adenovirus type 5 were downloaded from the RCSB database (accession number 1P30 and 3TG7). The three-dimensional structure models of the hexon of human adenovirus type 3 GB strain and type 7 Goman strain were constructed by the Swiss-Model online server, then visualized with the PyMol software. All hexon models from the three strains were similar, so the GB strain model was selected for illustration. Figure 1C shows the three-dimensional structure model of the human adenovirus hexon.

The three colors (red, green, and blue) represent the three hexon subunits. The adenovirus hexon divides into three parts: the base region (bottom), the neck region (middle), and the tower region (top). The three monomer necks of the hexon are entangled, and each tower region includes an L1 loop of a monomer neck and an L2 loop of another adjacent monomer neck. The red box in Figure 1C on the lower left shows a tower region, and the picture on the left corner shows an enlarged image of the tower region. As shown by the enlarged image, the tower region of the human adenovirus hexon contains a single L1 Loop (colored green) and another single L2 Loop (colored red) on the neck.

#### Evolutionary Trajectory of Human Adenovirus Hexon

The projection mode is the side view, and the monomer in the hexon trimer displays the ribbon view (Figure 1B). The color range defined above was adopted for coloring the monomer. Highly conserved residues are shown in gray. Variable residues (variability index above 0.6) are shown in red. The evolutionary trajectory of the human adenovirus hexon shows the non-uniform distribution of the mutations. The conservative gray and blue sites with lower variability are concentrated at the bottom, and the highly variable red and yellow spots are concentrated on the top.

The adenovirus hexon was divided into base, neck, and tower regions to clarify the evolutionary trajectory and homology index distribution of the different adenovirus hexon regions, according to the hexon homology and folding process of adenoviruses.

**FIGURE 2:**
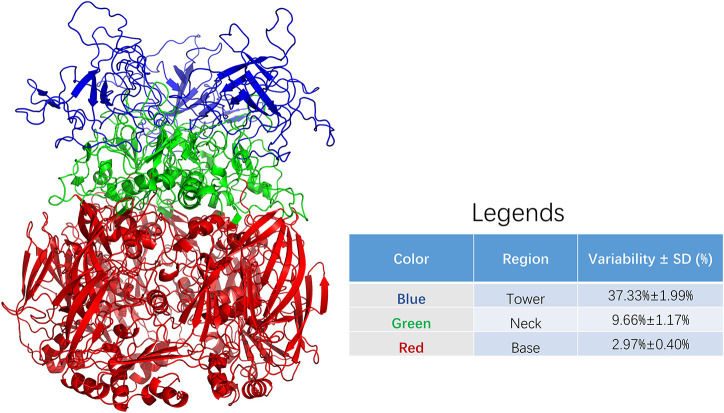
Sequence variability among regions of adenoviral hexon. The adenoviral hexon was divided into three regions including the tower (colored blue), neck (colored green) and bottom (colored red). The variability of each residue based on amino acid sequence alignment from each region was summarized in the legends. The table in legends showed statistically significant differences with *F* = 331.190 and *P* < 0.001.

Figure 2 shows the three regions of the adenovirus hexon, blue shows the tower region, red shows the base region, and green shows the neck region. The partition results showed that the hexon tower region contains 198 amino acid residues, the neck region contains 177, and the basal region contains 569 amino acid residues. The tower region, located on the top of the hexon, hosts the main neutralizing adenovirus epitopes. The secondary structure of the tower region is mainly random coils, and the region was located on the outer surface of the adenovirus nucleocapsid. The base region, situated at the bottom of the hexon, is the core structure of the adenovirus hexon. The secondary structure of the base region is a beta-sheet barrel. The neck connects the adenovirus hexon tower and basal regions and is key for entangling the two adjacent tower regions.

**FIGURE 3:**
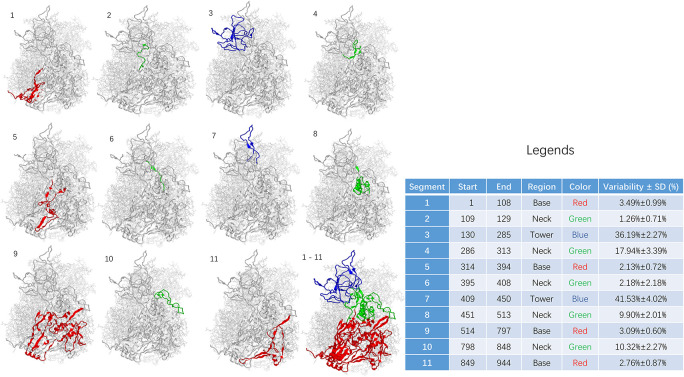
Sequence variability among segments of adenoviral hexon. The base region including segment 1, 5, 9, and 11 was shown in RED. The neck region including segment 2, 4, 6, 8, and 10 was shown in GREEN. The tower region including segment 3 and 7 was shown in BLUE. Statistics for the table: *F* = 69.119 and *P* < 0.001.

The average variability of the adenovirus hexon tower region is 37.33%, the strongest, with a 1.99% standard deviation. The base region has 2.97% variability, the least variable, with a 0.40% standard deviation. The neck region has 9.66% variability and 1.17% standard deviation. The variability between these three regions is significantly different (*P* < 0.001). Therefore, the amino acid sequence variability of the hexon and the three-dimensional structure partition are correlated, and the tower region caused the most sequence variability. The colors of the regions marked in Figure 2 correspond to Figure 3. These charts can be used to map the evolutionary trajectory of adenovirus hexon.

The sequence of the hexon monomer is divided into 11 fragments, including four fragments in the base region, five in the neck, and two fragments in the tower region based on variability and structure. From N to C terminus, the segments were listed as one segment (base), two segments (neck), three segments (tower), four segments (neck), five segments (base), six segments (neck), seven segments (tower), eight segments (neck), nine segments (base), ten segments (neck), and 11 segments (base).

The 11 fragments in the hexon monomer sequence fold into the ordered three-dimensional structure of the hexon (Figure 3). Figure 3 is divided into 12 sub-pictures from the upper left corner, and the corresponding segment number is marked in the upper left corner of each sub-picture. The first to the eleventh subgraphs corresponded to 11 segments, respectively. The twelfth subgraph in the lower right corner is the overall folded shape of all 11 segments of the monomer in the hexon trimer. The red area is the base region, namely segments 1, 5, 9, and 11. The green area is the neck, containing segments 2, 4, 6, 8, and 10; the blue area is the tower region, containing segments 3 and 7.

The sequence variability of 11 segments on the human adenovirus hexon monomer was counted and summarized in the Figure 3 legend to explore the relationship between the folding segmentation of the adenovirus hexon and the evolutionary trajectory. The distribution of variability in each segment is non-uniform (Figure 3). Overall, the tower region has the highest variability, the neck and the base regions have the lowest variability. Among the 11 segments, segment 7 of the tower area has the highest variability (41.53%). Segment 3 of the tower region has the second-highest variability (36.19%), and segment 2 of the neck region has the lowest variability (1.26%). Segment 5 of the base has the second-lowest variability (2.13%).

### Predicting the Common Ancestor of Adenoviral Hexon Proteins

#### Prediction of the Common Ancestor Sequence

Four datasets were constructed using the stratified sampling method based on the homogeneity of hexon protein sequences to evaluate the influence of dataset selection methods on constructing common ancestor sequences. The first dataset, named T74, includes the 74 hexon sequences of representative human adenovirus strains. The second dataset, named S7, comprises 23 typical strains from seven human adenoviruses species, including one strain from species G, two strains of species F, and four strains of species A to E. The third and fourth datasets were constructed according to the amino acid sequence similarity of the clustering results from the CD-HIT software. The homology criterion was 95% for the third dataset, and the adenoviral strains were divided into 47 groups. The homogeneity of hexon sequences in each group was at least 95%, the sequence similarity between different groups was less than 95%. One strain was randomly selected from each group as the representative strain, and the subset with 47 hexon sequences was named DB95. The fourth subset was selected using the 90% similarity criteria, and the adenoviral strains were divided into 13 groups. The homogeneity of hexon sequences in each group was at least 90%, the homology between different groups was less than 90%. One representative strain was selected from each group to form a subset of 13 hexon sequences named DB90.

The common ancestor sequences predicted based on different sequence datasets have significantly different in reliability (*P* < 0.001, Table 2). The highest reliability of the common ancestor sequence is in DB95N1, predicted using the DB95 dataset, with 0.994 probability and 0.989–0.999 confidence interval. The ancestor sequence T74N1 has the lowest reliability, predicted from the full-type dataset T74, with 0.968 probability and 0.964–0.973 confidence interval. In general, the ancestral sequences had over 0.9 credibility, indicating that the adenovirus hexon is highly conserved in the structure, in line with the results mentioned above on the evolutionary trace of adenoviral hexon.

**TABLE 2:** Confidential of ancestral sequence based on different datasets.

Subset	No. of seq	Ancestor	Length (aa)	Possibility (95% CI)
T74	74	T74N1	938	0.968 (0.964–0.973)
S7	23	S7N1	940	0.990 (0.985–0.994)
DB90	13	DB90N1	938	0.973 (0.969–0.978)
DB95	47	DB95N1	939	0.994 (0.989–0.999)

**F* = 27.115 and *P* < 0.001.*

Before selecting DB95 as the best dataset for constructing the common ancestor sequence of the human adenovirus hexon, the reasons for the higher reliability of this dataset were explored. Thus, the predicted value of each residue position was expanded according to the amino acid sequence by taking the position of each residue in the amino acid sequence as one dimension and the predicted probability value as the other dimension. These values were plotted as Supplementary Figure 4.

The predicted probability value of each residue position on each ancestral sequence was divided into three regional parts, including the tower, neck, and base, to analyze the influence of the three-dimensional structure partition of adenovirus hexon on the reconstruction of ancestral sequence (Table 3). There are significant differences in the prediction probability among different ancestral sequences and regions (Table 3). Overall, the DB95N1 subset has the highest prediction probability, and T74N1, the lowest. The base area has the highest overall prediction probability, followed by the neck area, and the tower area has the lowest. The DB95 dataset achieved the best prediction effect in the tower region, an important factor for considering the DB95N1 sequence as the best predictor.

**TABLE 3:** Confident probability of candidate sequences among regions.

Ancestor	Base	Neck	Tower	Average
DB90N1	0.997	0.977	0.902	0.973
DB95N1	1.000	0.998	0.973	0.994
S7N1	1.000	0.996	0.955	0.990
T74N1	0.989	0.977	0.902	0.968
Average	0.997	0.987	0.933	0.981

**F* = 194.443 and *P* < 0.001.*

As per the probability of reliability, the DB95N1 ancestor sequence constructed by the DB95 dataset was selected as the prediction result of the human adenoviruses common ancestor sequence. The predicted DB95N1 sequence was renamed HexN1. The HexN1 prediction result has 939 amino acid residues, and the HexN1 sequence in FASTA format can be found in the Supplementary Material.

The HexN1 sequence was added to the adenovirus hexon dataset, and the sequence alignment was performed using the MUSCLE software (version 3.6). Groups were defined using the MEGA software (version 10.0.1), according to adenovirus species. The homology matrix among the HexN1 and the hexon from each species of human adenoviruses was calculated as percentages (Table 4). The common ancestor sequence of human adenovirus HexN1 is evolutionarily located in the middle of the seven human adenovirus species, A to G, and is roughly equivalent to the representative strains of each adenovirus species (Table 4). The average homology between HexN1 and the seven human adenovirus species, A to G, is 86.40, 88.80, 83.90, 91.60, 90.00, 86.40, and 86.80%, respectively. Species D had the highest homogeneity (91.60%), and the lowest was in species A and F (86.40%). Meanwhile, the intraspecies homogeneity of the hexon is 82.30–88.90%. The distances between the HexN1 sequence and the adenoviruses are roughly equal and suitable for the common ancestor sequence of human adenoviral hexon protein.

**TABLE 4:** The similarity among different types of human adenovirus and HexN1.

	HexN1	HAdV-A	HAdV-B	HAdV-C	HAdV-D	HAdV-E	HAdV-F	HAdV-G
HexN1	100.00%	86.40%	88.80%	83.90%	91.60%	90.00%	86.40%	86.80%
HAdV-A	86.40%	100.00%	82.30%	82.70%	84.00%	82.80%	86.80%	87.70%
HAdV-B	88.80%	82.30%	100.00%	80.60%	85.70%	86.90%	82.50%	83.40%
HAdV-C	83.90%	82.70%	80.60%	100.00%	80.90%	81.60%	82.30%	84.00%
HAdV-D	91.60%	84.00%	85.70%	80.90%	100.00%	86.80%	83.80%	83.60%
HAdV-E	90.00%	82.80%	86.90%	81.60%	86.80%	100.00%	83.10%	83.00%
HAdV-F	86.40%	86.80%	82.50%	82.30%	83.80%	83.10%	100.00%	88.90%
HAdV-G	86.80%	87.70%	83.40%	84.00%	83.60%	83.00%	88.90%	100.00%

#### Homology Modeling of the Common Ancestor Hexon

The hexon common ancestor sequence HexN1 was submitted to the Swiss-Model online server for homology modeling, and the protein structure model was named N1M1. The Gromacs software optimized the structure of the homology modeling with the conjugate-gradient and the steepest-descent optimization methods. The side, top, and bottom views of N1M1 were visualized *via* the PyMol software and integrated into Supplementary Figure 2. The structural pattern of the hexon of the adenovirus strain is similar between the ancestral and current strains (Supplementary Figure 2).

### Serum ELISA Assay

**FIGURE 4:**
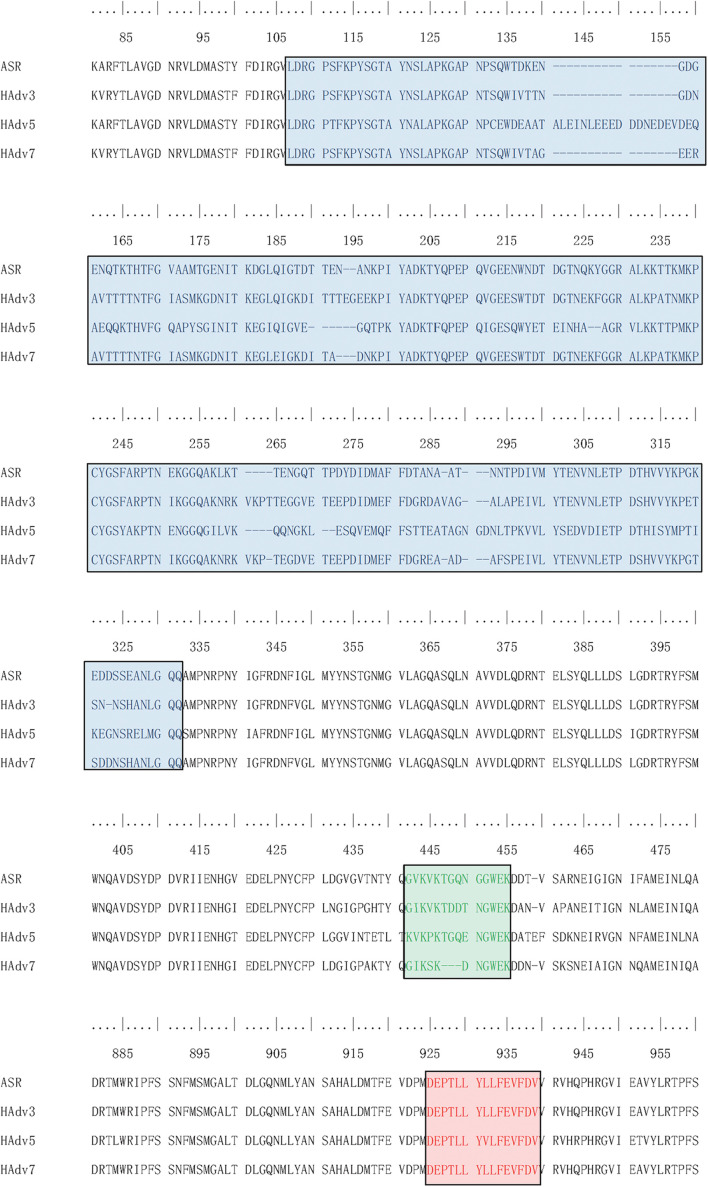
Sequence Alignment of peptides and proteins synthesized for ELISA. Sequences boxed in Blue were expressed as antigen protein segments, named as ASR, ADV3, ADV5 and ADV7; Sequences boxed in Green were synthesized as antigen peptides, named as ASR, ADV3, ADV5 and ADV7; Sequences boxed in Red were synthesized as antigen peptides, named as HexC1.

**FIGURE 5:**
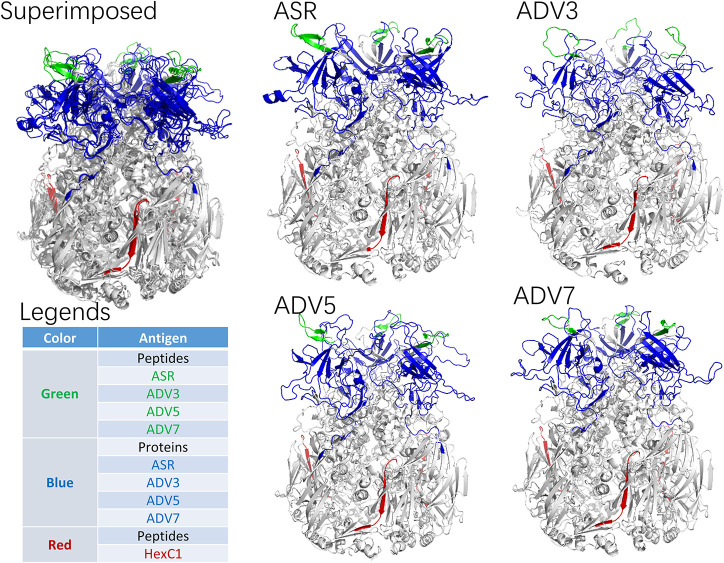
Spatial Location of synthesized antigen peptides and proteins on Hexon Trimers in this study. The ASR was the hexon model of ancestral sequences, also called N1M1 in main text. ADV3 was the hexon model of human adenovirus type 3 strain GB. The ADV5 was the hexon model of human adenovirus type 5, which was adopted from 1P30 in RCSB (PDB) database. The ADV7 was the hexon model of human adenovirus type 7 strain Gomen. The antigen polypeptides were colored with red or green, while the antigen protein segments were colored with blue.

According to the adenovirus evolutionary trajectory and the distribution of neutralizing epitopes, the linear neutralizing epitopes in the first and second loop region of the hexon neck were selected as the type-specific epitope. The epitope in the basal region of the hexon was selected as the conserved epitope. Figure 4 shows the sequence alignment of the antigens and polypeptides used in this study. The sequence alignment of the full dataset is provided as Supplementary Files. The antigen protein segments and polypeptides were located on the hexon structural models (Figure 5). From Figure 5, the antigen proteins are colored blue, while the polypeptides are colored green or red. Figures 4, 5 had the same coloring scheme. All the four protein segments (blue) are composed of the Loop 1 region, which are parts of the hexon tower region (Figure 5). The four polypeptides (green) were derived from the loop 2 region, part of the hexon tower region. The polypeptide HexC1 was conservative among the different types of adenoviruses, and was buried beneath the hexon base region.

The serological enzyme-linked immunosorbent assays (ELISA) entailed both the prokaryotic system expressed protein segments and the synthetic antigen peptides. The full-length sequences of antigen proteins are provided as Supplementary Files, and the peptide sequence contents are summarized in Table 5. Polypeptides 1–4 are the linear neutralizing epitopes located in the second loop of the hexon neck region, originally from the human adenovirus types 7, 3, 5, and the common ancestor of human adenovirus, respectively (Table 5). The fifth polypeptide is a conserved epitope in the base region of the human adenovirus hexon. The antigen proteins and polypeptides were inoculated in white rabbits to harvest positive control sera. All positive control sera titers were above 1:2,000, and the cross-reaction titers between the positive control sera did not exceed 1:50.

**TABLE 5:** Peptides used in serum ELISA assay.

ID	Name	Sequence (N to C)	Length (aa)	MW (d)	PI
1	ASR	GVKVKTGQNGGWEK	14	1,487.87	10.24
2	AD3	GIKVKTDDTNGWEK	14	1,590.92	6.35
3	AD5	KVKPKTGQENGWEK	14	1,629.03	10.07
4	AD7	GIKSKDNGWEK	11	1,261.52	9.12
5	HexC1	DEPTLLYLLFEVFDV	15	1,813.26	3.30

*MW, molecular weight; PI, Isoelectric point.*

Serum samples were collected from 337 healthy volunteers, including 161 males and 176 females. The age of volunteers ranged from 18 to 74 years, while the median age is 55. The lower quartile age is 44 years, while the upper quartile age is 64 years. The ELISA detected whether these sera could specifically react with the five antigen peptides and four protein segments above. The seropositive and negative results of serum ELISA assays are summarized in Tables 6, 7. Among the 337 healthy volunteers, 170 cases (50.45%) were positive for anti-human adenovirus 3 (Table 6). There were 124 positive cases (36.80%) for anti-human adenovirus 7 and 118 positive cases (35.01%) for anti-human adenovirus 5. Twenty-five cases (7.42%) were positive for the polypeptide from the common ancestor virus. Meanwhile, 134 cases (39.76%) were positive for the anti-adenovirus conservative region. The highest antigen, the human adenovirus antibody, had 170 positive cases (50.45%) (Table 7). The human adenovirus type 7 had 145 positive cases (43.03%), the second-highest rate. The anti-human adenovirus type 5 was third, with 123 positive cases (36.50%). The common ancestor protein had 23 positive cases (6.82%), the lowest rate.

**TABLE 6:** Human serum reaction with adenoviral antigen peptides.

Peptides	Negative (%)	Positive (%)	Total (%)
AD3	167 (49.55)	170 (50.45)	337 (100)
AD5	219 (64.99)	118 (35.01)	337 (100)
AD7	213 (63.20)	124 (36.80)	337 (100)
ASR	312 (92.58)	25 (7.42)	337 (100)
HexC1	203 (60.24)	134 (39.76)	337 (100)
Total	1,114 (66.11)	571 (33.89)	1,685 (100)

*Proportions are indicated as percentages in parentheses.*

*Chi-Square = 153.281 and *P* < 0.001.*

**TABLE 7:** Human serum reaction with adenoviral antigen proteins.

Protein	Negative (%)	Positive (%)	Total (%)
AD3	163 (48.37)	174 (51.63)	337 (100)
AD5	214 (63.50)	123 (36.50)	337 (100)
AD7	192 (56.97)	145 (43.03)	337 (100)
ASR	314 (93.18)	23 (6.82)	337 (100)
Total	883 (65.50)	465 (34.50)	1,348 (100)

*Proportions are indicated as percentages in parentheses.*

*Chi-Square = 169.441 and *P* < 0.001.*

In summary, the ancestral sequence of hexon possessed the lowest positive rates (6.82–7.42%) in serum ELISA tests, and the human adenovirus type 3 had the highest positive rates (50.45–51.63%). The human adenovirus types 7 and 5 had 35.10–43.03% positive rates. However, the conserved polypeptide, HexC1, had moderate positive rates (39.76%), lower than the highest rates of ADV3 (50.45%). The structural location could explain this varied positivity phenomenon on the hexon trimer. As shown in Figure 5, the HexC1 polypeptide was buried under the surface of the hexon trimer, probably impeding antibody binding. No significant difference among sex or age groups had been revealed in this survey (data not shown), possibly due to the demographic structure of the target population was aging.

## Discussion

In this paper, the molecular evolutionary analysis of the human adenovirus genome verified that the adenovirus hexon is the most conserved protein among the three major structural proteins on the adenoviral capsid. The phylogenetic history from the hexon amino acid sequence is consistent with the traditional classification of adenoviruses. An evolutionary trace map of the adenoviral hexon was drawn by projecting the homology and variability indexes onto the structure of the hexon protein trimer, revealing a more accurate picture that distinguishes the tower and neck regions of the adenoviral hexon amino acid sequences. Four reference datasets were designed from the evolutionary trajectory analysis, and four common ancestor sequences of human adenovirus hexon candidates were constructed. By comparing the credibility of the common ancestor sequences, the representative sequence dataset DB95 screened according to the homology index performed best. The selection criterion for this subset is that the minimum homology difference between each sequence in the dataset is 95%.

The adenoviral hexon was divided into three regions and 11 segments based on sequence variability and protein structures. At the same time, their spatial position was projected onto the structural model of hexon trimers (Figures 1–3). Before this report, there were two published partitioning methods for the adenovirus hexon. One account is purely based on sequence alignments (Ebner et al., 2005), and the other is based on protein structure (Rux et al., 2003).

Ebner et al. (2005) analyzed the variability distribution of the human adenovirus hexon nucleic acid and amino acid sequences. The hexon amino acid sequence was divided into two loop regions (variable and L regions) and three conserved regions. Ebner chose the human adenovirus species C type 2 as the reference strain to number the hexon partition table. Residues 1–136 were the C1 region, residues 137–315 were the L1 region, residues 316–418 were the C3 region, residues 419–459 were the L2 region, and residues 460–961 were the C4 region. Ebner et al. (2005) further divided the L1 region into three small segments: V1 (residues 137–221), C2 (residues 222–248), and the base segment, V2 (residues 249–315). The Ebner et al. (2005) hexon partition only considered the amino acid sequence in the hexon primary structure and the coding region variability of the corresponding nucleotide sequence, excluding the three-dimensional structure of the hexon protein.

The first three-dimensional structure of the adenovirus hexon was determined in 1986 when the resolution of the structural chemistry was low and underestimated the model (Roberts et al., 1986). John et al. divided the hexon into eight regions and 13 segments based on the three-dimensional structure of the adenovirus hexon (Rux et al., 2003). The John et al. partition also chose the adenovirus C type 2 strain as the reference for numbering the hexon amino acid sequences. The NT region of John et al. contains one segment (residues 1–55), the V1 region contains three segments (residues 56 to144 and 335–405). The DE1 region contains one segment (residues 115–334); the FG1 region contains one segment (residues 406–569), and the VC region contains two segments (residues 638–660, and 938–967). The V2 region contains three segments (residues 669–699, 730–787, and 884–937). The DE2 region contained one segment (residues 700–729), and the FG2 region contained one segment (residues 788–883).

In this paper, the two methods of sequence and structure were combined, and the evolutionary trajectory projected the variability of the adenovirus amino acid sequence onto the three-dimensional structure of the adenovirus hexon, thereby simplifying the adenovirus hexon partition and dividing it into three regions. In this report, the reference strain of the division was adjusted to the human adenovirus type 3. The adenovirus species C type 2 hexon has the first published hexon protein structure (Roberts et al., 1986), while the adenoviral species B type 3 was dominant in China (Duan et al., 2021). The evolutionary trace map in this report may improve the design or modify the adenoviral hexon proteins.

To the best of our knowledge, the reconstruction for common ancestral sequences of human adenovirus has not been reported to date. This report reasonably predicted the common ancestor sequence of adenoviral hexon. The concept of common ancestor sequence is a molecular evolution theory in biology, but the calculation methods are mathematical class average sequence algorithms. According to the theories from epidemiology and statistics, the average value will be affected by sampling errors. Therefore, when calculating the average value, ensure that the sample accurately reflects the actual population distribution to avoid bias. Data from sequence databases, including virology databases, are unfortunately biased. Many published sequence data are of little biological significance.

This paper employed two sampling schemes: the stratified method, and the sampling based on sequence differences, to construct four datasets. These datasets were named typical type, typical species, 95% homology, and 90% homology datasets, respectively. The 95% homology dataset performed the best for constructing the common ancestor sequence. This paper established the idea of constructing the common ancestor sequence using the best dataset, a technique suitable for studying the adenovirus hexon, and a reference for constructing common ancestor sequences in the evolutionary history of other viruses.

The serum positive rates for adenovirus in this study were similar to global reports. From this study, 35.01% of the volunteers were serum positive against epitopes of the human adenovirus type 5. In the United States and Europe, the serum positive rates against human adenovirus type 5 are 35.1 and 39.8%, respectively (Mast et al., 2010). This study also detected 50.45% serum positive results among volunteers against the human adenovirus type 3, with a 1:200 ratio. A recent report shows that pre-existing anti-adenovirus serum ratio above 1:200 in populations reduces the protecting effect of the adenoviral vector-based COVID-19 vaccine by over 50% (Chen et al., 2021). A recent survey in southern China shows that 41.6% of sera sampled from adults tested positive against the human adenovirus type 3, with an over 1:129 ratio (Tian et al., 2016). The serum positive rate for human adenovirus type 7 in adults from southern China is 54% (Tian et al., 2020), a rate higher than the 36.8% reported in this study.

The adenoviral strains isolated from animal hosts are important sources for novel vector development. The pre-existing immunity to these animal-originated hexons in the healthy population is 12.7% for the chimpanzee adenovirus type 68 (Zhang et al., 2013). However, when millions of people were inoculated with the chimpanzee adenovirus vectored COVID-19 vaccines, rare but life-threatening thrombotic adverse reactions were reported (Tobaiqy et al., 2021). The ancestral sequence of human adenovirus hexon differed from current strains, but it was still rationally humanized. The ancestral hexon sequence shares base and neck regions with modern strains and may not cause serious adverse effects in humans.

There also were several limitations in this report. First of all, the structure of the ancestral hexon in this study was predicted using homology modeling. It should be better to solve this protein structure using experimental methods including NMR or X-Ray diffraction. In addition, only the adult population with an aging structure had been surveyed in this report, so it would be necessary to take the same serum tests in the pediatric population in the future. Finally, the seroprevalence of rare human adenoviral types or animal adenoviruses in the Harbin city had never been carried out, though this kind of data should provide strong evidences supporting the value of ancestral sequence reconstruction. It was needed to continue with the seroprevalence survey for adenoviral immunity in healthy populations. The reference data set with 95% homology is the most appropriate for constructing the human adenovirus hexon sequence. Based on the common ancestor sequence, the homology modeling method is suitable for constructing a basic and reasonable structure of the human adenoviral hexon common ancestor. The positive sera against neutralizing epitopes on the common ancestor of adenoviral hexon were relatively rare among healthy adults. This common ancestor hexon predicted in this report has a good application prospect in the improvement of adenovirus vectors.

The reference dataset with 95% homology is the most appropriate for constructing the human adenovirus hexon sequence. The homology modeling method is suitable for constructing a basic and reasonable human adenoviral hexon common ancestor structure based on the common ancestor sequence. The positive sera against neutralizing epitopes on the common ancestor of adenoviral hexon were rare among healthy adults. The common ancestor hexon predicted in this report is a good candidate for improving adenovirus vectors.

## Data Availability Statement

The original contributions presented in the study are included in the article/Supplementary Material, further inquiries can be directed to the corresponding author/s.

## Ethics Statement

The studies involving human participants were reviewed and approved by Ethics Committee of Harbin Medical University. The patients/participants provided their written informed consent to participate in this study. The animal study was reviewed and approved by Ethics Committee of Harbin Medical University.

## Author Contributions

YW, ZZ, TD, and ZQ designed this study and prepared the manuscript. GQ, WG, and TD enrolled volunteers and collected the specimens. YW, ZZ, and TD conducted the evolutionary analysis. YW, ZZ, LS, HG, XD, FL, YG, and TD performed the biological experiments. YW and TD analyzed the data and made the figures and tables. All authors reviewed and approved the manuscript.

## Conflict of Interest

The authors declare that the research was conducted in the absence of any commercial or financial relationships that could be construed as a potential conflict of interest.

## Publisher’s Note

All claims expressed in this article are solely those of the authors and do not necessarily represent those of their affiliated organizations, or those of the publisher, the editors and the reviewers. Any product that may be evaluated in this article, or claim that may be made by its manufacturer, is not guaranteed or endorsed by the publisher.
