# Supplementary material for: Immunological Study of Reconstructed Common Ancestral Sequence of Adenovirus Hexon Protein
Source: Front Microbiol. 2021 Oct 27;12:717047. doi: 10.3389/fmicb.2021.717047 (PMC8578728; doi:10.3389/fmicb.2021.717047)
Supplement: Supplementary file 1 [file Data_Sheet_1.DOCX]

Supplementary Material

# Supplementary Data

## Datasets used in this study

The sequence dataset HAdvGnm959, Gnm353, T74, DB95, DB90 and S7 has been summarized in attached Excel sheets, including accession number in GenBank and strain information. The ancestral sequences predicted in this study was shown in section 1.2. The sequence alignment of ancestral sequence and 3 modern strains were displayed in the figure section. An extended version of sequence alignment including ancestral sequence and 74 modern strains was attached in supplementary files in FASTA format.

There were three hexon structure models from RCSB (PDB) database were used in this report, whose accession numbers were 1P30, 3TG7 and 2OBE. The structure files 1P30 and 3TG7 represented hexon protein monomer of the human adenovirus type 5. The structure file 2OBE revealed the hexon protein trimer of the chimpanzee adenovirus type 68. These structures served as templates in homology modelling of adenoviral hexon structures. All these structure files were freely available from RCSB database, so they were not included in the supplementary files. The protein models predicted in this report were available from the authors on request.

## Common Ancestral Sequence

The ancestral sequence HexN1 predicted from hexon dataset DB95 was displayed in FASTA format.

>HexN1

MATPSMMPQWAYMHIAGQDASEYLSPGLVQFARATDTYFSLGNKFRNPTVAPTHDVTTDRSQRLTLRFVPVDREDTTYSYKARFTLAVGDNRVLDMASTYFDIRGVLDRGPSFKPYSGTAYNSLAPKGAPNSSQWEQKETNGGGDKTETHTFGVAAMGGENITKKGLQIGTDTTENENKPIYADKTYQPEPQVGEENWQETENFYGGRALKKETKMKPCYGSFARPTNEKGGQAKFKTGENGQPTKDFDIDMAFFDTPGGTLTGNTEYKADIVMYTENVNLETPDTHVVYKPGKEDDSSEINLVQQSMPNRPNYIGFRDNFVGLMYYNSTGNMGVLAGQASQLNAVVDLQDRNTELSYQLLLDSLGDRTRYFSMWNSAVDSYDPDVRIIENHGVEDELPNYCFPLDGSGTNAAYQGVKVKTGQNDEWEKDTNVAARNQICKGNIYAMEINLQANLWKSFLYSNVALYLPDSYKYTPANVTLPTNTNTYEYMNGRVVAPSLVDAYINIGARWSLDPMDNVNPFNHHRNAGLRYRSMLLGNGRYVPFHIQVPQKFFAIKNLLLLPGSYTYEWNFRKDVNMILQSSLGNDLRVDGASVRFDSVNLYATFFPMAHNTASTLEAMLRNDTNDQSFNDYLSAANMLYPIPAKATNVPISIPSRNWAAFRGWSFTRLKTKETPSLGSGFDPYFVYSGSIPYLDGTFYLNHTFKKVSIMFDSSVSWPGNDRLLTPNEFEIKRSVDGEGYNVAQCNMTKDWFLVQMLSHYNIGYQGFHVPEGYKDRMYSFFRNFQPMSRQVVDEINYKDYKAVTLPFQHNNSGFTGYLAPTMRQGQPYPANFPYPLIGSTAVPSVTQKKFLCDRVMWRIPFSSNFMSMGALTDLGQNMLYANSAHALDMTFEVDPMDEPTLLYLLFEVFDVVRVHQPHRGVIEAVYLRTPFSAGNATT

# Supplementary Figures and Tables

## Supplementary Tables

**Supplementary Table 1** The species and genotypes distribution of the complete genome sequences of human adenoviruses from GenBank

| Species | Type | Seqs | Species | Type | Seqs | Species | Type | Seqs |
| --- | --- | --- | --- | --- | --- | --- | --- | --- |
| A | 12 | 4 | D | 22 | 3 | D | 70 | 1 |
| A | 18 | 1 | D | 23 | 2 | D | 71 | 1 |
| A | 31 | 3 | D | 24 | 1 | D | 72 | 1 |
| A | 61 | 1 | D | 25 | 1 | D | 73 | 1 |
| B | 3 | 57 | D | 26 | 1 | D | 74 | 1 |
| B | 7 | 270 | D | 27 | 1 | D | 75 | 1 |
| B | 11 | 10 | D | 28 | 2 | D | 81 | 1 |
| B | 14 | 50 | D | 29 | 2 | D | 82 | 1 |
| B | 16 | 2 | D | 30 | 1 | D | 83 | 1 |
| B | 21 | 38 | D | 32 | 1 | D | 84 | 1 |
| B | 34 | 3 | D | 33 | 1 | D | 85 | 1 |
| B | 35 | 4 | D | 36 | 1 | D | 86 | 1 |
| B | 50 | 1 | D | 37 | 19 | D | 87 | 1 |
| B | 55 | 66 | D | 38 | 1 | D | 88 | 1 |
| B | 66 | 1 | D | 39 | 1 | D | 91 | 1 |
| B | 68 | 1 | D | 42 | 1 | D | 92 | 1 |
| B | 76 | 1 | D | 43 | 2 | D | 93 | 1 |
| B | 77 | 1 | D | 44 | 1 | D | 94 | 1 |
| B | 78 | 1 | D | 45 | 1 | D | 95 | 1 |
| B | 79 | 1 | D | 46 | 1 | D | 96 | 1 |
| B | NA. | 15 | D | 47 | 1 | D | 97 | 1 |
| C | 1 | 13 | D | 48 | 1 | D | 98 | 1 |
| C | 2 | 17 | D | 49 | 1 | D | 99 | 1 |
| C | 5 | 9 | D | 51 | 1 | D | 100 | 1 |
| C | 6 | 7 | D | 53 | 12 | D | 101 | 1 |
| C | 57 | 1 | D | 54 | 8 | D | 102 | 1 |
| C | 104 | 1 | D | 56 | 9 | D | 103 | 1 |
| C | NA. | 38 | D | 58 | 2 | D | NA. | 9 |
| D | 8 | 66 | D | 59 | 1 | E | 4 | 73 |
| D | 9 | 2 | D | 60 | 2 | F | 40 | 8 |
| D | 10 | 5 | D | 62 | 1 | F | 41 | 27 |
| D | 13 | 1 | D | 63 | 1 | F | NA. | 2 |
| D | 15 | 10 | D | 64 | 5 | G | 52 | 1 |
| D | 17 | 3 | D | 65 | 1 | NA. | NA. | 8 |
| D | 19 | 7 | D | 67 | 1 |  |  |  |
| D | 20 | 1 | D | 69 | 1 | Total |  | 959 |

Notes: There were 959 complete genome sequences of human adenovirus (HAdv) published on GenBank till 2021 August. In these sequences, there were 7 species (from A to G) and 101 genotypes. The most abundant sequences came from HAdv species B genotype 7, and the proportion was up to 28.2% (270/959). The number 270 was underlined. NA. : Not Available. Seqs: The counts of sequences.

**Supplementary Table 2** Accession Number and Protein IDs of Sequences in Dataset for Ancestral Sequence Reconstruction

| No. | Accession | ProeinID | Species | Type | T74 | DB95 | DB90 | S7 |
| --- | --- | --- | --- | --- | --- | --- | --- | --- |
| 1 | AB330093 | BAG48789 | A | 12 | Yes | Yes | Yes | Yes |
| 2 | DQ149610 | AAZ99994 | A | 18 | Yes | No | No | Yes |
| 3 | AM749299 | CAO78638 | A | 31 | Yes | Yes | Yes | Yes |
| 4 | JF964962 | AEK79922 | A | 61 | Yes | No | No | Yes |
| 5 | AY599834 | AAW33168 | B | 3 | Yes | Yes | Yes | Yes |
| 6 | AF065065 | AAD03663 | B | 7 | Yes | No | No | Yes |
| 7 | FJ841904 | ACZ06785 | B | 11 | Yes | No | No | No |
| 8 | DQ149612 | AAZ99996 | B | 14 | Yes | Yes | No | No |
| 9 | AY601636 | AAW33444 | B | 16 | Yes | Yes | Yes | No |
| 10 | AY008279 | AAG21823 | B | 21 | Yes | Yes | No | Yes |
| 11 | AY737797 | AAW33485 | B | 34 | Yes | Yes | No | No |
| 12 | AY271307 | AAP92351 | B | 35 | Yes | Yes | Yes | No |
| 13 | DQ149643 | ABA00027 | B | 50 | Yes | Yes | No | No |
| 14 | KF908851 | AIS92536 | B | 55 | Yes | No | No | No |
| 15 | JN860676 | AET87148 | B | 66 | Yes | No | No | Yes |
| 16 | JN860678 | AET87230 | B | 68 | Yes | No | No | No |
| 17 | LC177352 | BAW32492 | B | 79 | Yes | No | No | Yes |
| 18 | AF534906 | AAQ10553 | C | 1 | Yes | Yes | No | Yes |
| 19 | AF542120 | AAO24095 | C | 2 | Yes | Yes | Yes | Yes |
| 20 | AY601635 | AAW65514 | C | 5 | Yes | Yes | Yes | Yes |
| 21 | HQ413315 | ADV03661 | C | 6 | Yes | Yes | No | Yes |
| 22 | HQ003817 | ADM46153 | C | 57 | Yes | Yes | No | Yes |
| 23 | DQ149614 | AAZ99998 | D | 8 | Yes | Yes | No | Yes |
| 24 | AB245425 | BAE66671 | D | 9 | Yes | Yes | Yes | No |
| 25 | DQ149615 | AAZ99999 | D | 10 | Yes | Yes | No | No |
| 26 | DQ149616 | ABA00000 | D | 13 | Yes | Yes | No | No |
| 27 | AB562586 | BAJ22290 | D | 15 | Yes | No | No | No |
| 28 | HQ910407 | ADY18429 | D | 17 | Yes | Yes | No | No |
| 29 | DQ149618 | ABA00002 | D | 19 | Yes | Yes | No | No |
| 30 | DQ149619 | ABA00003 | D | 20 | Yes | Yes | No | Yes |
| 31 | DQ149620 | ABA00004 | D | 22 | Yes | Yes | No | No |
| 32 | DQ149621 | ABA00005 | D | 23 | Yes | Yes | No | No |
| 33 | DQ149622 | ABA00006 | D | 24 | Yes | Yes | No | No |
| 34 | DQ149623 | ABA00007 | D | 25 | Yes | Yes | No | No |
| 35 | DQ149624 | ABA00008 | D | 26 | Yes | Yes | Yes | No |
| 36 | DQ149625 | ABA00009 | D | 27 | Yes | Yes | No | No |
| 37 | FJ824826 | ACQ91158 | D | 28 | Yes | Yes | No | Yes |
| 38 | AB562587 | BAJ22326 | D | 29 | Yes | No | No | No |
| 39 | DQ149628 | ABA00012 | D | 30 | Yes | Yes | No | No |
| 40 | DQ149629 | ABA00013 | D | 32 | Yes | Yes | No | No |
| 41 | DQ149630 | ABA00014 | D | 33 | Yes | No | No | No |
| 42 | GQ384080 | ACY04472 | D | 36 | Yes | Yes | No | No |
| 43 | DQ149632 | ABA00016 | D | 37 | Yes | No | No | No |
| 44 | DQ149633 | ABA00017 | D | 38 | Yes | Yes | No | Yes |
| 45 | DQ149634 | ABA00018 | D | 39 | Yes | No | No | No |
| 46 | DQ149635 | ABA00019 | D | 42 | Yes | Yes | No | No |
| 47 | DQ149636 | ABA00020 | D | 43 | Yes | Yes | No | No |
| 48 | DQ149637 | ABA00021 | D | 44 | Yes | Yes | No | No |
| 49 | DQ149638 | ABA00022 | D | 45 | Yes | Yes | No | No |
| 50 | DQ149639 | ABA00023 | D | 46 | Yes | Yes | No | No |
| 51 | DQ149640 | ABA00024 | D | 47 | Yes | Yes | No | No |
| 52 | EF153473 | ABO61301 | D | 48 | Yes | Yes | No | No |
| 53 | DQ149641 | ABA00025 | D | 49 | Yes | Yes | No | No |
| 54 | DQ149642 | ABA00026 | D | 51 | Yes | Yes | No | No |
| 55 | AB605243 | BAJ46350 | D | 53 | Yes | No | No | No |
| 56 | NC_012959 | YP_003038612 | D | 54 | Yes | No | No | No |
| 57 | HM770721 | ADM66117 | D | 56 | Yes | No | No | No |
| 58 | HQ883276 | ADW95419 | D | 58 | Yes | Yes | Yes | No |
| 59 | JF799911 | AEI91289 | D | 59 | Yes | No | No | No |
| 60 | HQ007053 | AEK87026 | D | 60 | Yes | No | No | No |
| 61 | JN162671 | AEL78842 | D | 62 | Yes | Yes | No | No |
| 62 | JN935766 | AEV92966 | D | 63 | Yes | No | No | No |
| 63 | EF121005 | ABN10535 | D | 64 | Yes | No | No | No |
| 64 | AP012285 | BAL41721 | D | 65 | Yes | No | No | No |
| 65 | AP012302 | BAL63187 | D | 67 | Yes | No | No | No |
| 66 | JN226748 | AFK92217 | D | 69 | Yes | No | No | No |
| 67 | KP641339 | AKI33526 | D | 70 | Yes | No | No | No |
| 68 | KF268207 | AGT76762 | D | 71 | Yes | No | No | No |
| 69 | KF268335 | AGT78001 | D | 72 | Yes | No | No | Yes |
| 70 | KX827426 | APD28385 | D | 73 | Yes | No | No | No |
| 71 | AF065064 | AAD03660 | E | 4 | Yes | Yes | No | Yes |
| 72 | KU162869 | AMQ95234 | F | 40 | Yes | Yes | Yes | Yes |
| 73 | DQ315364 | ACH90432 | F | 41 | Yes | Yes | Yes | Yes |
| 74 | DQ923122 | ABK35044 | G | 52 | Yes | Yes | Yes | Yes |

Notes: T74, DB95, DB90 and S7 were dataset names described in the main text. These datasets were all made of human adenoviral strains. Accessions and protein IDs came from the GenBank database. Yes: this item was selected for the dataset. No: this item was not selected for the dataset.

## Supplementary Figures

....|....| ....|....| ....|....| ....|....| ....|....| ....|....| ....|....| ....|....|

5 15 25 35 45 55 65 75

ASR MATPSMMPQW AYMHIAGQDA SEYLSPGLVQ FARATDTYFS LGNKFRNPTV APTHDVTTDR SQRLTLRFVP VDREDTTYSY

HAdv3 MATPSMMPQW AYMHIAGQDA SEYLSPGLVQ FARATDTYFS MGNKFRNPTV APTHDVTTDR SQRLMLRFVP VDREDNTYSY

HAdv5 MATPSMMPQW SYMHISGQDA SEYLSPGLVQ FARATETYFS LNNKFRNPTV APTHDVTTDR SQRLTLRFIP VDREDTAYSY

HAdv7 MATPSMMPQW AYMHIAGQDA SEYLSPGLVQ FARATDTYFS MGNKFRNPTV APTHDVTTDR SQRLMLRFVP VDREDNTYSY

....|....| ....|....| ....|....| ....|....| ....|....| ....|....| ....|....| ....|....|

85 95 105 115 125 135 145 155

ASR KARFTLAVGD NRVLDMASTY FDIRGVLDRG PSFKPYSGTA YNSLAPKGAP NPSQWTDKEN ---------- -------GDG

HAdv3 KVRYTLAVGD NRVLDMASTF FDIRGVLDRG PSFKPYSGTA YNSLAPKGAP NTSQWIVTTN ---------- -------GDN

HAdv5 KARFTLAVGD NRVLDMASTY FDIRGVLDRG PTFKPYSGTA YNALAPKGAP NPCEWDEAAT ALEINLEEED DDNEDEVDEQ

HAdv7 KVRYTLAVGD NRVLDMASTF FDIRGVLDRG PSFKPYSGTA YNSLAPKGAP NTSQWIVTAG ---------- -------EER

....|....| ....|....| ....|....| ....|....| ....|....| ....|....| ....|....| ....|....|

165 175 185 195 205 215 225 235

ASR ENQTKTHTFG VAAMTGENIT KDGLQIGTDT TEN--ANKPI YADKTYQPEP QVGEENWNDT DGTNQKYGGR ALKKTTKMKP

HAdv3 AVTTTTNTFG IASMKGDNIT KEGLQIGKDI TTTEGEEKPI YADKTYQPEP QVGEESWTDT DGTNEKFGGR ALKPATNMKP

HAdv5 AEQQKTHVFG QAPYSGINIT KEGIQIGVE- -----GQTPK YADKTFQPEP QIGESQWYET EINHA--AGR VLKKTTPMKP

HAdv7 AVTTTTNTFG IASMKGDNIT KEGLEIGKDI TA---DNKPI YADKTYQPEP QVGEESWTDT DGTNEKFGGR ALKPATKMKP

....|....| ....|....| ....|....| ....|....| ....|....| ....|....| ....|....| ....|....|

245 255 265 275 285 295 305 315

ASR CYGSFARPTN EKGGQAKLKT ----TENGQT TPDYDIDMAF FDTANA-AT- --NNTPDIVM YTENVNLETP DTHVVYKPGK

HAdv3 CYGSFARPTN IKGGQAKNRK VKPTTEGGVE TEEPDIDMEF FDGRDAVAG- --ALAPEIVL YTENVNLETP DSHVVYKPET

HAdv5 CYGSYAKPTN ENGGQGILVK ----QQNGKL --ESQVEMQF FSTTEATAGN GDNLTPKVVL YSEDVDIETP DTHISYMPTI

HAdv7 CYGSFARPTN IKGGQAKNRK VKP-TEGDVE TEEPDIDMEF FDGREA-AD- --AFSPEIVL YTENVNLETP DSHVVYKPGT

....|....| ....|....| ....|....| ....|....| ....|....| ....|....| ....|....| ....|....|

325 335 345 355 365 375 385 395

ASR EDDSSEANLG QQAMPNRPNY IGFRDNFIGL MYYNSTGNMG VLAGQASQLN AVVDLQDRNT ELSYQLLLDS LGDRTRYFSM

HAdv3 SN-NSHANLG QQAMPNRPNY IGFRDNFVGL MYYNSTGNMG VLAGQASQLN AVVDLQDRNT ELSYQLLLDS LGDRTRYFSM

HAdv5 KEGNSRELMG QQSMPNRPNY IAFRDNFIGL MYYNSTGNMG VLAGQASQLN AVVDLQDRNT ELSYQLLLDS IGDRTRYFSM

HAdv7 SDDNSHANLG QQAMPNRPNY IGFRDNFVGL MYYNSTGNMG VLAGQASQLN AVVDLQDRNT ELSYQLLLDS LGDRTRYFSM

....|....| ....|....| ....|....| ....|....| ....|....| ....|....| ....|....| ....|....|

405 415 425 435 445 455 465 475

ASR WNQAVDSYDP DVRIIENHGV EDELPNYCFP LDGVGVTNTY QGVKVKTGQN GGWEKDDT-V SARNEIGIGN IFAMEINLQA

HAdv3 WNQAVDSYDP DVRIIENHGI EDELPNYCFP LNGIGPGHTY QGIKVKTDDT NGWEKDAN-V APANEITIGN NLAMEINIQA

HAdv5 WNQAVDSYDP DVRIIENHGT EDELPNYCFP LGGVINTETL TKVKPKTGQE NGWEKDATEF SDKNEIRVGN NFAMEINLNA

HAdv7 WNQAVDSYDP DVRIIENHGI EDELPNYCFP LDGIGPAKTY QGIKSK---D NGWEKDDN-V SKSNEIAIGN NQAMEINIQA

....|....| ....|....| ....|....| ....|....| ....|....| ....|....| ....|....| ....|....|

485 495 505 515 525 535 545 555

ASR NLWRSFLYSN VALYLPDSYK YTPANVTLPE NTNTYEYMNG RVVAPSLVDT YINIGARWSL DPMDNVNPFN HHRNAGLRYR

HAdv3 NLWRSFLYSN VALYLPDVYK YTPPNITLPT NTNTYEYMNG RVVSPSLVDS YINIGARWSL DPMDNVNPFN HHRNAGLRYR

HAdv5 NLWRNFLYSN IALYLPDKLK YSPSNVKISD NPNTYDYMNK RVVAPGLVDC YINLGARWSL DYMDNVNPFN HHRNAGLRYR

HAdv7 NLWRSFLYSN VALYLPDVYK YTPTNITLPA NTNTYEYMNG RVVSPSLVDS YINIGARWSL DPMDNVNPFN HHRNAGLRYR

....|....| ....|....| ....|....| ....|....| ....|....| ....|....| ....|....| ....|....|

565 575 585 595 605 615 625 635

ASR SMLLGNGRYV PFHIQVPQKF FAIKNLLLLP GSYTYEWNFR KDVNMILQSS LGNDLRVDGA SIRFDSINLY ATFFPMAHNT

HAdv3 SMLLGNGRYV PFHIQVPQKF FAVKNLLLLP GSYTYEWNFR KDVNMVLQSS LGNDLRTDGA TISFTSINLY ATFFPMAHNT

HAdv5 SMLLGNGRYV PFHIQVPQKF FAIKNLLLLP GSYTYEWNFR KDVNMVLQSS LGNDLRVDGA SIKFDSICLY ATFFPMAHNT

HAdv7 SMLLGNGRYV PFHIQVPQKF FAVKNLLLLP GSYTYEWNFR KDVNMVLQSS LGNDLRTDGA TISFTSINLY ATFFPMAHNT

....|....| ....|....| ....|....| ....|....| ....|....| ....|....| ....|....| ....|....|

645 655 665 675 685 695 705 715

ASR ASTLEAMLRN DTNDQSFNDY LSAANMLYPI PANATNVPIS IPSRNWAAFR GWSFTRLKTK ETPSLGSGFD PYFVYSGSIP

HAdv3 ASTLEAMLRN DTNDQSFNDY LSAANMLYPI PANATNIPIS IPSRNWAAFR GWSFTRLKTK ETPSLGSGFD PYFVYSGSIP

HAdv5 ASTLEAMLRN DTNDQSFNDY LSAANMLYPI PANATNVPIS IPSRNWAAFR GWAFTRLKTK ETPSLGSGYD PYYTYSGSIP

HAdv7 ASTLEAMLRN DTNDQSFNDY LSAANMLYPI PANATNIPIS IPSRNWAAFR GWSFTRLKTK ETPSLGSGFD PYFVYSGSIP

....|....| ....|....| ....|....| ....|....| ....|....| ....|....| ....|....| ....|....|

725 735 745 755 765 775 785 795

ASR YLDGTFYLNH TFKKVSIMFD SSVSWPGNDR LLTPNEFEIK RSVDGEGYNV AQCNMTKDWF LVQMLSHYNI GYQGFYVPEG

HAdv3 YLDGTFYLNH TFKKVSIMFD SSVSWPGNDR LLSPNEFEIK RTVDGEGYNV AQCNMTKDWF LVQMLANYNI GYQGFYIPEG

HAdv5 YLDGTFYLNH TFKKVAITFD SSVSWPGNDR LLTPNEFEIK RSVDGEGYNV AQCNMTKDWF LVQMLANYNI GYQGFYIPES

HAdv7 YLDGTFYLNH TFKKVSIMFD SSVSWPGNDR LLSPNEFEIK RTVDGEGYNV AQCNMTKDWF LVQMLANYNI GYQGFYIPEG

....|....| ....|....| ....|....| ....|....| ....|....| ....|....| ....|....| ....|....|

805 815 825 835 845 855 865 875

ASR YKDRMYSFFR NFQPMSRQVV DEINYKDYKA VTLPFQHNNS GFVGYLAPTM RQGQPYPANY PYPLIGTTAV PSVTQKKFLC

HAdv3 YKDRMYSFFR NFQPMSRQVV DEVNYTDYKA VTLPYQHNNS GFVGYLAPTM RQGEPYPANY PYPLIGTTAV KSVTQKKFLC

HAdv5 YKDRMYSFFR NFQPMSRQVV DDTKYKDYQQ VGILHQHNNS GFVGYLAPTM REGQAYPANF PYPLIGKTAV DSITQKKFLC

HAdv7 YKDRMYSFFR NFQPMSRQVV DEVNYTDYKA VTLPYQHNNS GFVGYLAPTM RQGEPYPANY PYPLIGTTAV KSVTQKKFLC

....|....| ....|....| ....|....| ....|....| ....|....| ....|....| ....|....| ....|....|

885 895 905 915 925 935 945 955

ASR DRTMWRIPFS SNFMSMGALT DLGQNMLYAN SAHALDMTFE VDPMDEPTLL YLLFEVFDVV RVHQPHRGVI EAVYLRTPFS

HAdv3 DRTMWRIPFS SNFMSMGALT DLGQNMLYAN SAHALDMTFE VDPMDEPTLL YLLFEVFDVV RVHQPHRGVI EAVYLRTPFS

HAdv5 DRTLWRIPFS SNFMSMGALT DLGQNLLYAN SAHALDMTFE VDPMDEPTLL YVLFEVFDVV RVHRPHRGVI ETVYLRTPFS

HAdv7 DRTMWRIPFS SNFMSMGALT DLGQNMLYAN SAHALDMTFE VDPMDEPTLL YLLFEVFDVV RVHQPHRGVI EAVYLRTPFS

....|.

965

ASR AGNATT

HAdv3 AGNATT

HAdv5 AGNATT

HAdv7 AGNATT

**Supplementary Figure 1 The multiple sequence alignment of adenoviral hexon protein based on ancestral sequence and 3 modern strains.**

Notes: The sequences of expressed protein segments were shown in blue. The polypeptides hosting epitopes on Loop 2 region of hexon were colored in green. The conserved epitope in base region used as the antigen polypeptide was colored in red. An alignment of 74 strains with ASR was provided as FASTA format in the supplementary files attached.

Side View

Top View

Bottom View


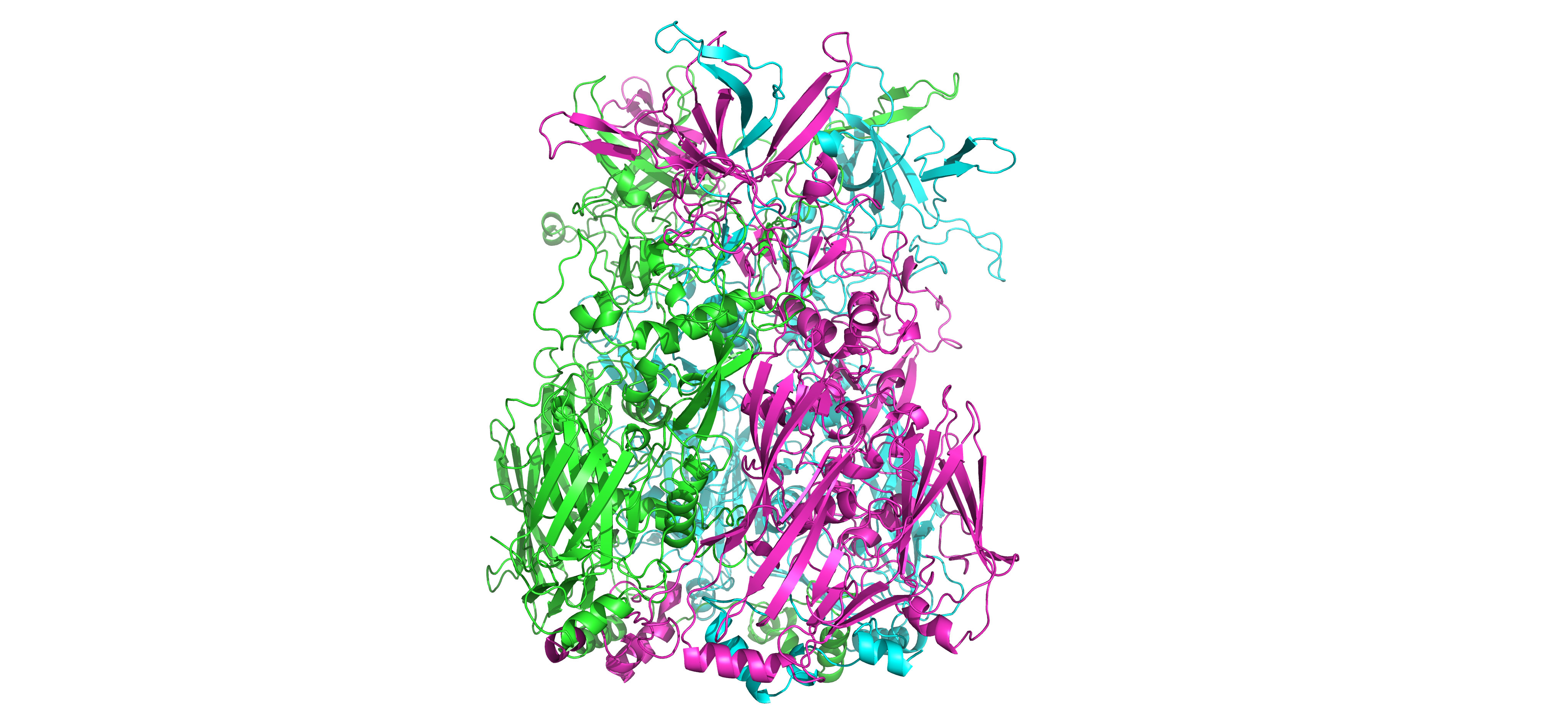

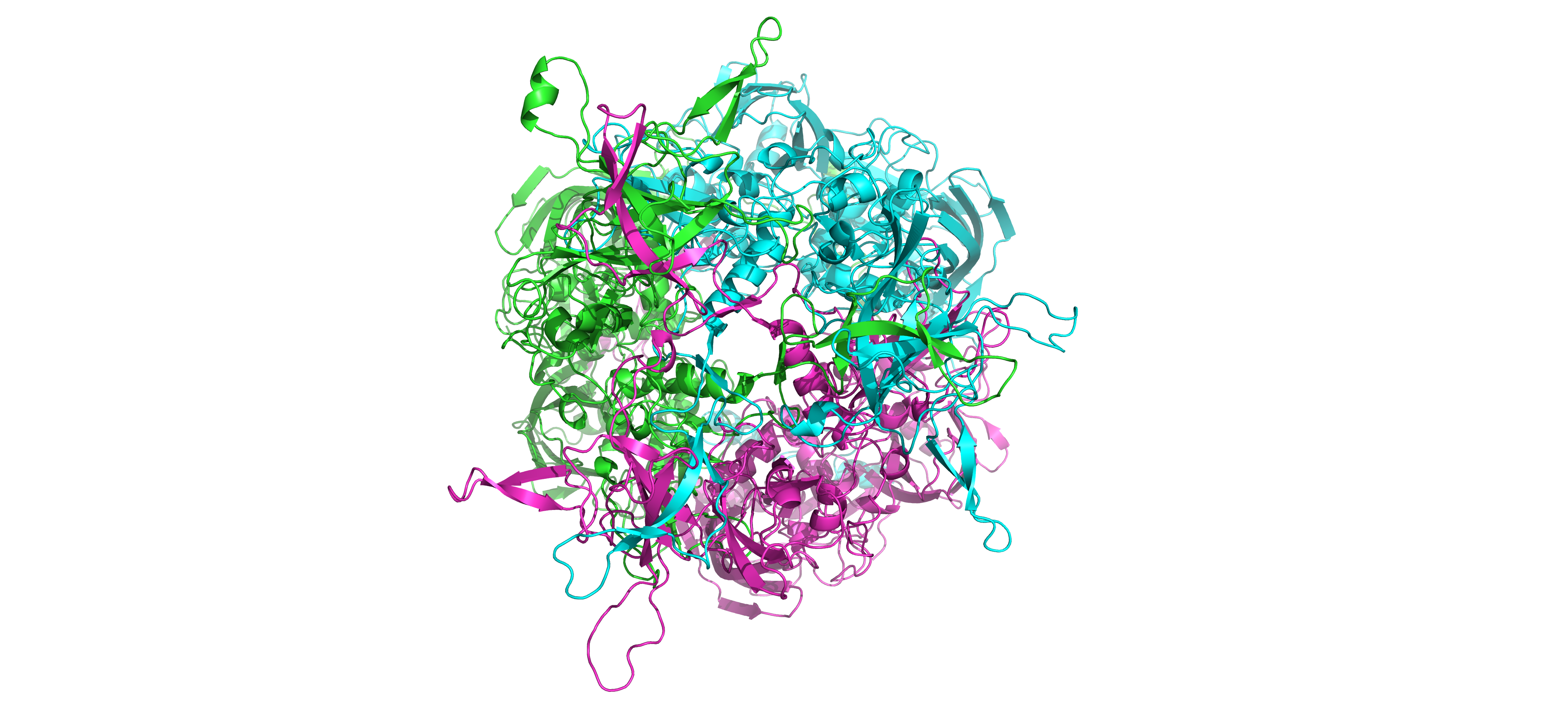

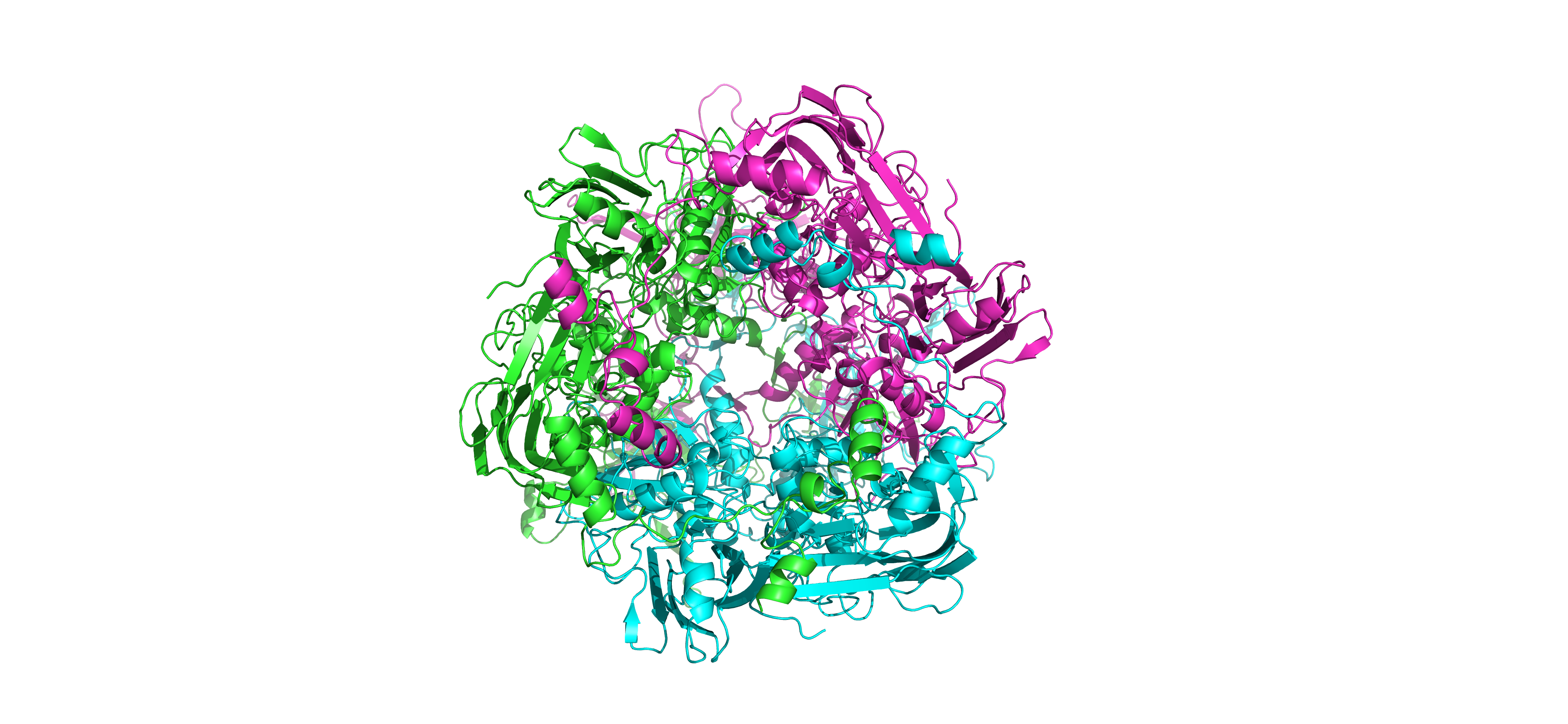


**Supplementary Figure 2 Three-dimensional structure of N1M1 protein.**


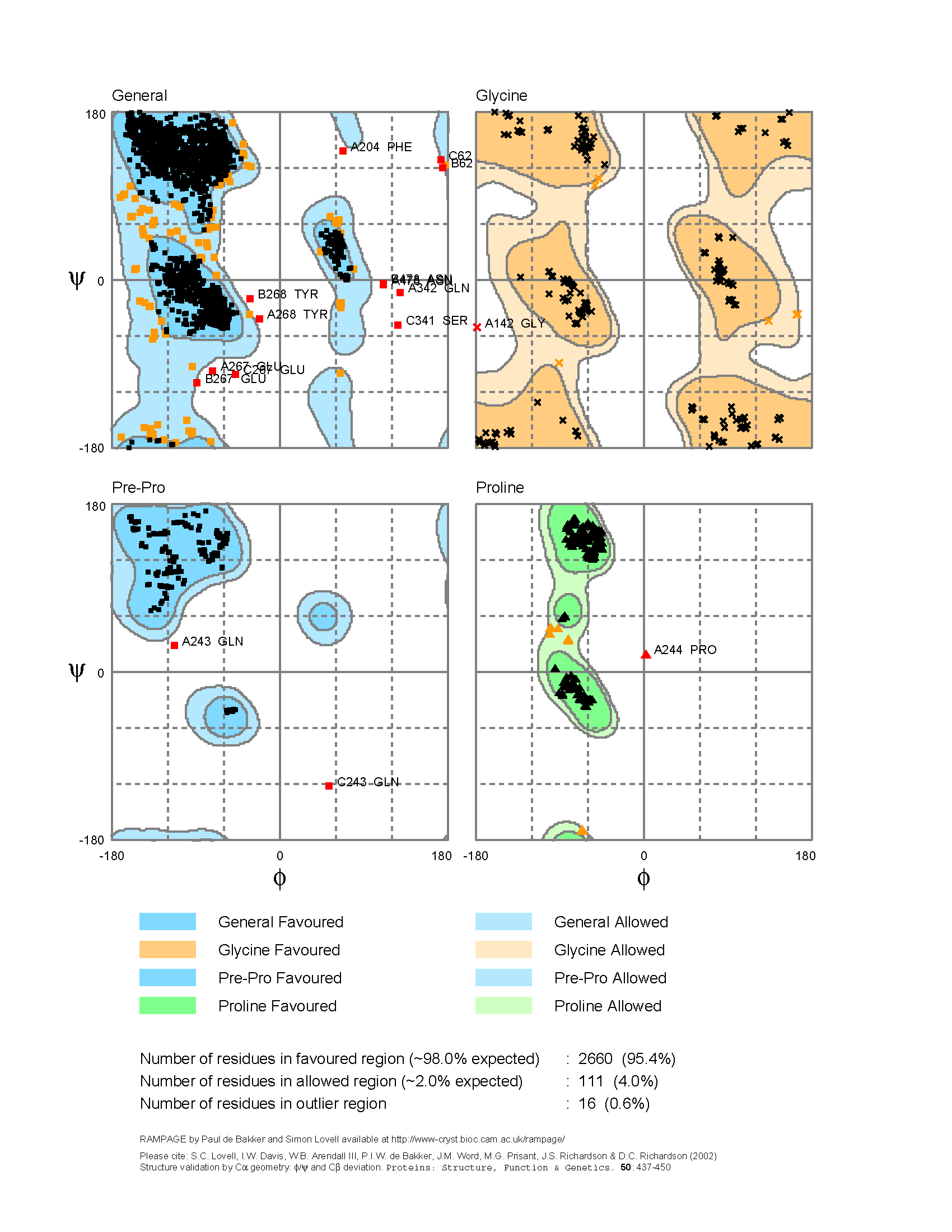


**Supplementary Figure 3 Ramachandran Plot of Ancestral Hexon Sequence’s model N1M1.**


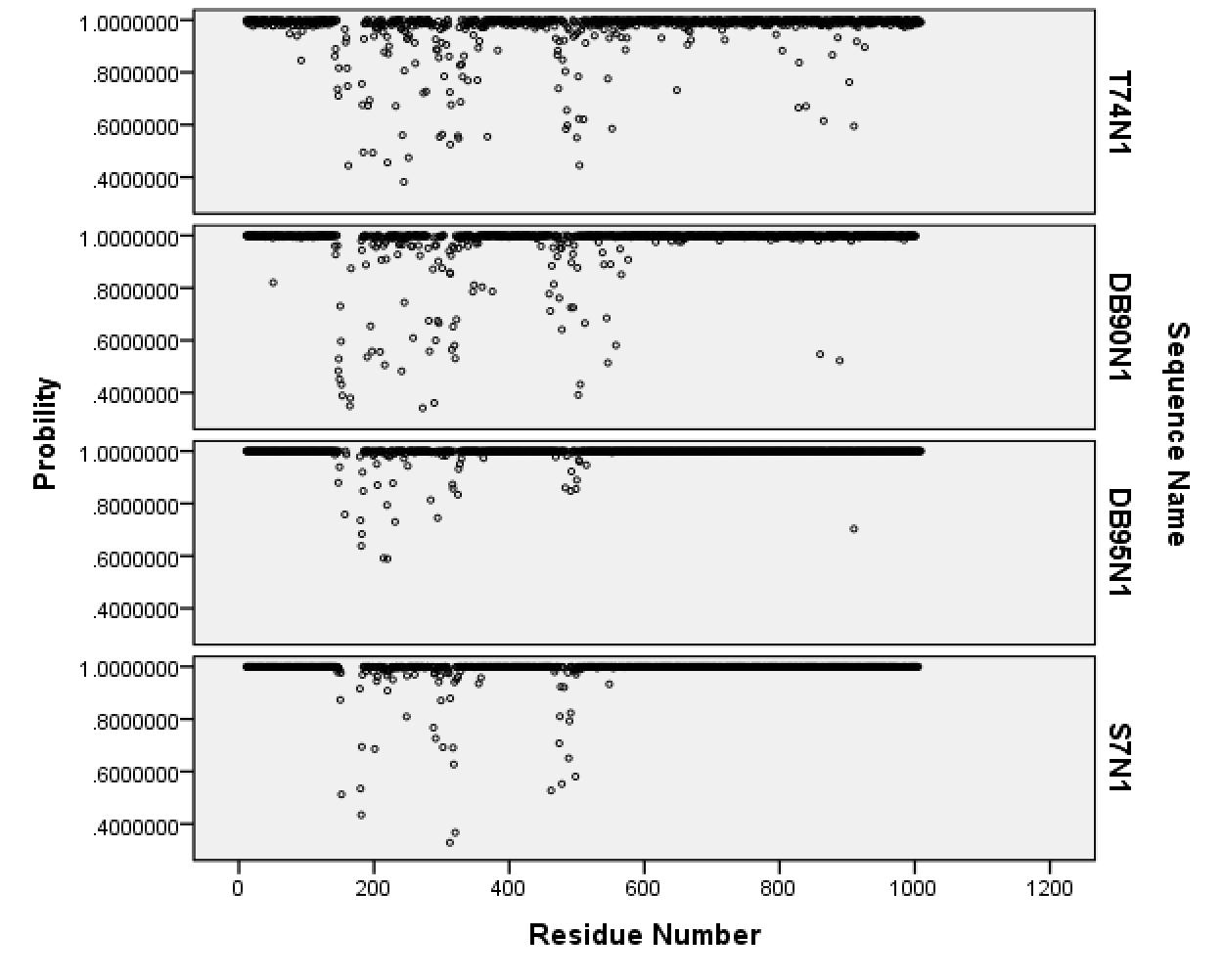


**Supplementary Figure 4** The distribution of confident probability for ancestral sequences.

The supplementary Figure 3 is a scatter plot of the probability of the four candidate common ancestor sequences from T74N1 to S7N1 using the amino acid sequence position as the abscissa and the predicted probability of each position as the ordinate as the N1 of the sequence alignment result. -11 shows that the probabilities of most regions are very close to 1, and the regions with a probability of less than 0.95 are mainly concentrated in the middle region of the linear coordinate of the hexon, that is, the region of amino acid residues 100 to 320, 440 to 520 has a large variation, These areas correspond to the tower area of the hexagonal three-dimensional structure and the adjacent part of the neck. The two-dimensional distribution characteristics found in the picture are relatively rough. In order to analyze the distribution of the common ancestor sequence prediction probability value in more detail, statistics need to be combined with the specific partition boundary positions in the three-dimensional structure model.
