# Supplementary material for: Immunological Study of Reconstructed Common Ancestral Sequence of Adenovirus Hexon Protein
Source: Front Microbiol. 2021 Oct 27;12:717047. doi: 10.3389/fmicb.2021.717047 (PMC8578728; doi:10.3389/fmicb.2021.717047)
Supplement: Supplementary file 2 [file Data_Sheet_2.DOCX]

....|....| ....|....| ....|....| ....|....| ....|....| ....|....|

5 15 25 35 45 55

HexN1 ---------- MATPSMMPQW AYMHIAGQDA SEYLSPGLVQ FARATDTYFS LGNKFRNPTV

B_3_AAW33168 ---------- MATPSMMPQW AYMHIAGQDA SEYLSPGLVQ FARATDTYFS MGNKFRNPTV

C_5_AAW65514 ---------- MATPSMMPQW SYMHISGQDA SEYLSPGLVQ FARATETYFS LNNKFRNPTV

B_7_AAD03663 ---------- MATPSMMPQW AYMHIAGQDA SEYLSPGLVQ FARATDTYFS MGNKFRNPTV

C_2_AAO24095 ---------- MATPSMMPQW SYMHISGQDA SEYLSPGLVQ FARATETYFS LNNKFRNPTV

C_1_AAQ10553 ---------- MATPSMMPQW SYMHISGQDA SEYLSPGLVQ FARATETYFS LNNKFRNPTV

C_57_ADM46153 ---------- MATPSMMPQW SYMHISGQDA SEYLSPGLVQ FARATETYFS LNNKFRNPTV

C_6_ADV03661 ---------- MATPSMMPQW SYMHISGQDA SEYLSPGLVQ FARATETYFS LNNKFRNPTV

D_37_ABA00016 ---------- MATPSMMPQW AYMHIAGQDA SEYLSPGLVQ FARATDTYFS LGNKFRNPTV

D_13_ABA00000 ---------- MATPSMMPQW AYMHIAGQDA SEYLSPGLVQ FARATDTYFS LGNKFRNPTV

D_70_AKI33526 MCLTARERAK MATPSMMPQW AYMHIAGQDA SEYLSPGLVQ FARATDTYFS LGNKFRNPTV

D_63_AEV92966 MCLTARERAK MATPSMMPQW AYMHIAGQDA SEYLSPGLVQ FARATDTYFS LGNKFRNPTV

D_72_AGT78001 MCLTARERAK MATPSMMPQW AYMHIAGQDA SEYLSPGLVQ FARATDTYFS LGNKFRNPTV

D_23_ABA00005 ---------- MATPSMMPQW AYMHIAGQDA SEYLSPGLVQ FARATDTYFS LGNKFRNPTV

D_24_ABA00006 ---------- MATPSMMPQW AYMHIAGQDA SEYLSPGLVQ FARATDTYFS LGNKFRNPTV

D_39_ABA00018 ---------- MATPSMMPQW AYMHIAGQDA SEYLSPGLVQ FARATDSYFS LGNKFRNPTV

D_43_ABA00020 ---------- MATPSMMPQW AYMHIAGQDA SEYLSPGLVQ FARATDTYFS LGNKFRNPTV

D_58_ADW95419 MCLTARERAK MATPSMMPQW AYMHIAGQDA SEYLSPGLVQ FARATDTYFS LGNKFRNPTV

D_33_ABA00014 ---------- MATPSMMPQW AYMHIAGQDA SEYLSPGLVQ FARATDTYFS LGNKFRNPTV

D_38_ABA00017 ---------- MATPSMMPQW AYMHIAGQDA SEYLSPGLVQ FARATDTYFS LGNKFRNPTV

D_36_ACY04472 ---------- MATPSMMPQW AYMHIAGQDA SEYLSPGLVQ FARATDTYFS LGNKFRNPTV

D_49_ABA00025 ---------- MATPSMMPQW AYMHIAGQDA SEYLSPGLVQ FARATDTYFS LGNKFRNPTV

D_8_AAZ99998 ---------- MATPSMMPQW AYMHIAGQDA SEYLSPGLVQ FARATDTYFS LGNKFRNPTV

D_46_ABA00023 ---------- MATPSMMPQW AYMHIAGQDA SEYLSPGLVQ FARATDTYFS LGNKFRNPTV

D_47_ABA00024 ---------- MATPSMMPQW AYMHIAGQDA SEYLSPGLVQ FARATDTYFS LGNKFRNPTV

D_44_ABA00021 ---------- MATPSMMPQW AYMHIAGQDA SEYLSPGLVQ FARATDTYFS LGNKFRNPTV

D_48_ABO61301 MCLTARERAK MATPSMMPQW AYMHIAGQDA SEYLSPGLVQ FARATDTYFS LGNKFRNPTV

D_22_ABA00004 ---------- MATPSMMPQW AYMHIAGQDA SEYLSPGLVQ FARATDTYFS LGNKFRNPTV

D_53_BAJ46350 ---------- MATPSMMPQW AYMHIAGQDA SEYLSPGLVQ FARATDTYFS LGNKFRNPTV

D_45_ABA00022 ---------- MATPSMMPQW AYMHIAGQDA SEYLSPGLVQ FARATDTYFS LGNKFRNPTV

D_26_ABA00008 ---------- MATPSMMPQW AYMHIAGQDA SEYLSPGLVQ FARATDTYFS LGNKFRNPTV

D_32_ABA00013 ---------- MATPSMMPQW AYMHIAGQDA SEYLSPGLVQ FARATDTYFS LGNKFRNPTV

D_73_APD28385 ---------- MATPSMMPQW AYMHIAGQDA SEYLSPGLVQ FARATDTYFS LGNKFRNPTV

D_67_BAL63187 ---------- MATPSMMPQW AYMHIAGQDA SEYLSPGLVQ FARATDTYFS LGNKFRNPTV

D_9_BAE66671 ---------- MATPSMMPQW AYMHIAGQDA SEYLSPGLVQ FARATDTYFS LGNKFRNPTV

D_54_YP_0030386 MCLTARERAK MATPSMMPQW AYMHIAGQDA SEYLSPGLVQ FARATDTYFS LGNKFRNPTV

D_17_ADY18429 MCLTARERAK MATPSMMPQW AYMHIAGQDA SEYLSPGLVQ FARATDTYFS LGNKFRNPTV

D_10_AAZ99999 ---------- MATPSMMPQW AYMHIAGQDA SEYLSPGLVQ FARATDTYFS LGNKFRNPTV

D_65_BAL41721 ---------- MATPSMMPQW AYMHIAGQDA SEYLSPGLVQ FARATDTYFS LGNKFRNPTV

D_62_AEL78842 ---------- MATPSMMPQW AYMHIAGQDA SEYLSPGLVQ FARATDTYFS LGNKFRNPTV

D_25_ABA00007 ---------- MATPSMMPQW AYMHIAGQDA SEYLSPGLVQ FARATDTYFS LGNKFRNPTV

D_59_AEI91289 MCLTARERAK MATPSMMPQW AYMHIAGQDA SEYLSPGLVQ FARATDTYFS LGNKFRNPTV

D_19_ABA00002 ---------- MATPSMMPQW AYMHIAGQDA SEYLSPGLVQ FARATDTYFS LGNKFRNPTV

D_64_ABN10535 MCLTARERAK MATPSMMPQW AYMHIAGQDA SEYLSPGLVQ FARATDTYFS LGNKFRNPTV

D_27_ABA00009 ---------- MATPSMMPQW AYMHIAGQDA SEYLSPGLVQ FARATDTYFS LGNKFRNPTV

D_28_ACQ91158 ---------- MATPSMMPQW AYMHIAGQDA SEYLSPGLVQ FARATDTYFS LGNKFRNPTV

D_20_ABA00003 ---------- MATPSMMPQW AYMHIAGQDA SEYLSPGLVQ FARATDTYFS LGNKFRNPTV

D_60_AEK87026 ---------- MATPSMMPQW AYMHIAGQDA SEYLSPGLVQ FARATDTYFS LGNKFRNPTV

D_71_AGT76762 MCLTARERAK MATPSMMPQW AYMHIAGQDA SEYLSPGLVQ FARATDTYFS LGNKFRNPTV

D_42_ABA00019 ---------- MATPSMMPQW AYMHIAGQDA SEYLSPGLVQ FARATDTYFS LGNKFRNPTV

D_51_ABA00026 ---------- MATPSMMPQW AYMHIAGQDA SEYLSPGLVQ FARATDTYFS LGNKFRNPTV

D_30_ABA00012 ---------- MATPSMMPQW AYMHIAGQDA SEYLSPGLVQ FARATDTYFS LGNKFRNPTV

D_29_BAJ22326 ---------- MATPSMMPQW AYMHIAGQDA SEYLSPGLVQ FARATDTYFS LGNKFRNPTV

D_56_ADM66117 MCLTARERAK MATPSMMPQW AYMHIAGQDA SEYLSPGLVQ FARATDTYFS LGNKFRNPTV

D_69_AFK92217 MCLTARERAK MATPSMMPQW AYMHIAGQDA SEYLSPGLVQ FARATDTYFS LGNKFRNPTV

D_15_BAJ22290 ---------- MATPSMMPQW AYMHIAGQDA SEYLSPGLVQ FARATDTYFS LGNKFRNPTV

B_35_AAP92351 ---------- MATPSMLPQW AYMHIAGQDA SEYLSPGLVQ FARATDTYFN LGNKFRNPTV

B_11_ACZ06785 ---------- MATPSMLPQW AYMHIAGQDA SEYLSPGLVQ FARATDTYFN LGNKFRNPTV

B_55_AIS92536 ---------- MATPSMLPQW AYMHIAGQDA SEYLSPGLVQ FARATDTYFN LGNKFRNPTV

B_21_AAG21823 ---------- MATPSMLPQW AYMHIAGQDA SEYLSPGLVQ FARATDTYFN LGNKFRNPTV

B_50_ABA00027 ---------- MATPSMLPQW AYMHIAGQDA SEYLSPGLVQ FARATDTYFN LGNKFRNPTV

B_14_AAZ99996 ---------- MATPSMLPQW AYMHIAGQDA SEYLSPGLVQ FARATDTYFN LGNKFRNPTV

B_34_AAW33485 ---------- MATPSMLPQW AYMHIAGQDA SEYLSPGLVQ FARATDTYFN LGNKFRNPTV

B_79_BAW32492 ---------- MATPSMLPQW AYMHIAGQDA SEYLSPGLVQ FARATDTYFN LGNKFRNPTV

B_68_AET87230 ---------- MATPSMMPQW AYMHIAGQDA SEYLSPGLVQ FARATDTYFS MGNKFRNPTV

B_66_AET87148 ---------- MATPSMMPQW AYMHIAGQDA SEYLSPGLVQ FARATDTYFS MGNKFRNPTV

E_4_AAD03660 ---------- MATPSMLPQW AYMHIAGQDA SEYLSPGLVQ FARATDTYFS LGNKFRNPTV

B_16_AAW33444 ---------- MATPSMMPQW AYMHIAGQDA SEYLSPGLVQ FARATDTYFS MGNKFRNPTV

A_61_AEK79922 ---------- MATPSMMPQW SYMHIAGQDA SEYLSPGLVQ FARATDTYFT LGNKFRNPTV

A_12_BAG48789 ---------- MATPSMMPQW SYMHIAGQDA SEYLSPGLVQ FARATDTYFT LGNKFRNPTV

A_18_AAZ99994 ---------- MATPSMMPQW SYMHIAGQDA SEYLSPGLVQ FARATDTYFT LGNKFRNPTV

A_31_CAO78638 ---------- MATPSMMPQW SYMHIAGQDA SEYLSPGLVQ FARATDTYFT LGNKFRNPTV

G_52_ABK35044 ---------- MATPSMMPQW SYMHIAGQDA SEYLSPGLVQ FARATDTYFS LGNKFRNPTV

F_41_ACH90432 ---------- MATPSMMPQW SYMHIAGQDA SEYLSPGLVQ FARATDTYFS LGNKFRNPTV

F_40_AMQ95234 ---------- MATPSMMPQW SYMHIAGQDA SEYLSPGLVQ FARATDTYFS LGNKFRNPTV

....|....| ....|....| ....|....| ....|....| ....|....| ....|....|

65 75 85 95 105 115

HexN1 APTHDVTTDR SQRLTLRFVP VDREDTTYSY KARFTLAVGD NRVLDMASTY FDIRGVLDRG

B_3_AAW33168 APTHDVTTDR SQRLMLRFVP VDREDNTYSY KVRYTLAVGD NRVLDMASTF FDIRGVLDRG

C_5_AAW65514 APTHDVTTDR SQRLTLRFIP VDREDTAYSY KARFTLAVGD NRVLDMASTY FDIRGVLDRG

B_7_AAD03663 APTHDVTTDR SQRLMLRFVP VDREDNTYSY KVRYTLAVGD NRVLDMASTF FDIRGVLDRG

C_2_AAO24095 APTHDVTTDR SQRLTLRFIP VDREDTAYSY KARFTLAVGD NRVLDMASTY FDIRGVLDRG

C_1_AAQ10553 APTHDVTTDR SQRLTLRFIP VDREDTAYSY KARFTLAVGD NRVLDMASTY FDIRGVLDRG

C_57_ADM46153 APTHDVTTDR SQRLTLRFIP VDREDTAYSY KARFTLAVGD NRVLDMASTY FDIRGVLDRG

C_6_ADV03661 APTHDVTTDR SQRLTLRFIP VDREDTAYSY KARFTLAVGD NRVLDMASTY FDIRGVLDRG

D_37_ABA00016 APTHDVTTDR SQRLTLRFVP VDREDTTYSY KARFTLAVGD NRVLDMASTY FDIRGVLDRG

D_13_ABA00000 APTHDVTTDR SQRLTLRFVP VDREDTTYSY KARFTLAVGD NRVLDMASTY FDIRGVLDRG

D_70_AKI33526 APTHDVTTDR SQRLTLRFVP VDREDTTYSY KARFTLAVGD NRVLDMASTY FDIRGVLDRG

D_63_AEV92966 APTHDVTTDR SQRLTLRFVP VDREDTTYSY KARFTLAVGD NRVLDMASTY FDIRGVLDRG

D_72_AGT78001 APTHDVTTDR SQRLTLRFVP VDREDTTYSY KARFTLAVGD NRVLDMASTY FDIRGVLDRG

D_23_ABA00005 APTHDVTTDR SQRLTLRFVP VDREDTTYSY KARFTLAVGD NRVLDMASTY FDIRGVLDRG

D_24_ABA00006 PPTHDVTTDR SQRLTLRFVP VDREDTRYLY KARFTLAVGD NRVLDMASTY FDIRGVLDRG

D_39_ABA00018 APTHDVTTDR SQRLTLRFVP VDREDTTYSY KARFTLAVGD NRVLDMASTY FDIRGVLDRG

D_43_ABA00020 APTHDVTTDR SQRLTLRFVP VDREDTTYSY KARFTLAVGD NRVLDMASTY FDIRGVLDRG

D_58_ADW95419 APTHDVTTDR SQRLTLRFVP VDREDTTYSY KARFTLAVGD NRVLDMASTY FDIRGVLDRG

D_33_ABA00014 APTHDVTTDR SQRLTLRFVP VDREDTTYSY KARFTLAVGD NRVLDMASTY FDIRGVLDRG

D_38_ABA00017 APTHDVTTDR SQRLTLRFVP VDREDTTYSY KARFTLAVGD NRVLDMASTY FDIRGVLDRG

D_36_ACY04472 APTHDVTTDR SQRLTLRFVP VDREDTTYSY KARFTLAVGD NRVLDMASTY FDIRGVLDRG

D_49_ABA00025 APTHDVTTDR SQRLTLRFVP VDREDTTYSY KARFTLAVGD NRVLDMASTY FDIRGVLDRG

D_8_AAZ99998 APTHDVTTDR SQRLTLRFVP VDREDTTYSY KARFTLAVGD NRVLDMASTY FDIRGVLDRG

D_46_ABA00023 APTHDVTTDR SQRLTLRFVP VDREDTTYSY KARLTLDVGD NRVLDMASTY FDIRGVLDRG

D_47_ABA00024 APTHDVTTDR SQRLTLRFVP VDREDTTYSY KARFTLAVGD NRVLDMASTY FDIRGVLDRG

D_44_ABA00021 APTHDVTTDR SQRLTLRFVP VDREDTTYSY KARFTLAVGD NRVLDMASTY FDIRGVLDRG

D_48_ABO61301 APTHDVTTDR SQRLTLRFVP VDREDTTYSY KARFTLAVGD NRVLDMASTY FDIRGVLDRG

D_22_ABA00004 APTHDVTTDR SQRLTLRFVP VDREDTTYSY KARFTLAVGD NRVLDMASTY FDIRGVLDRG

D_53_BAJ46350 APTHDVTTDR SQRLTLRFVP VDREDTTYSY KARFTLAVGD NRVLDMASTY FDIRGVLDRG

D_45_ABA00022 APTHDVTTDR SQRLTLRFVP VDREDTTYSY KARSTLDVGD NRVLDMASTY FDIRGVLDRG

D_26_ABA00008 APTHDVTTDR SQRLTLRFVP VDREATTYLY KARFTLAVGD NRVLDMASTY FDIRGVLDRG

D_32_ABA00013 APTHDVTTDR SQRLTLRFVP VDREDTTYSY KARFTLAVGD NRVLDMASTY FDIRGVLDRG

D_73_APD28385 APTHDVTTDR SQRLTLRFVP VDREDTTYSY KARFTLAVGD NRVLDMASTY FDIRGVLDRG

D_67_BAL63187 APTHDVTTDR SQRLTLRFVP VDREDTTYSY KARFTLAVGD NRVLDMASTY FDIRGVLDRG

D_9_BAE66671 APTHDVTTDR SQRLTLRFVP VDREDTTYSY KARFTLAVGD NRVLDMASTY FDIRGVLDRG

D_54_YP_0030386 APTHDVTTDR SQRLTLRFVP VDREDTTYSY KARFTLAVGD NRVLDMASTY FDIRGVLDRG

D_17_ADY18429 APTHDVTTDR SQRLTLRFVP VDREDTTYSY KARFTLAVGD NRVLDMASTY FDIRGVLDRG

D_10_AAZ99999 APTHDVTTDR SQRLTLRFVP VDREDTTYSY KARFTLAVGD NRVLDMASTY FDIRGVLDRG

D_65_BAL41721 APTHDVTTDR SQRLTLRFVP VDREDTTYSY KARFTLAVGD NRVLDMASTY FDIRGVLDRG

D_62_AEL78842 APTHDVTTDR SQRLTLRFVP VDREDTTYSY KARFTLAVGD NRVLDMASTY FDIRGVLDRG

D_25_ABA00007 APTHDVTTDR SQRLTLRFVP VDREDTTYSY KARFTLAVGD NRVLDMASTY FDIRGVLDRG

D_59_AEI91289 APTHDVTTDR SQRLTLRFVP VDREDTTYSY KARFTLAVGD NRVLDMASTY FDIRGVLDRG

D_19_ABA00002 APTHDVTTDR SQRLTLRFVP VDREDTTYSY KARFTLAVGD NRVLDMASTY FDIRGVLDRG

D_64_ABN10535 APTHDVTTDR SQRLTLRFVP VDREDTTYSY KARFTLAVGD NRVLDMASTY FDIRGVLDRG

D_27_ABA00009 APTHDVTTDR SQRLTLRFVP VDREDTTYSY KARFTLAVGD NRVLDMASTY FDIRGVLDRG

D_28_ACQ91158 APTHDVTTDR SQRLTLRFVP VDREDTTYSY KARFTLAVGD NRVLDMASTY FDIRGVLDRG

D_20_ABA00003 APTHDVTTDR SQRLTLRFVP VDREDTTYSY KARFTLAVGD NRVLDMASTY FDIRGVLDRG

D_60_AEK87026 APTHDVTTDR SQRLTLRFVP VDREDTTYSY KARFTLAVGD NRVLDMASTY FDIRGVLDRG

D_71_AGT76762 APTHDVTTDR SQRLTLRFVP VDREDTTYSY KARFTLAVGD NRVLDMASTY FDIRGVLDRG

D_42_ABA00019 APTHDVTTDR SQRLTLRFVP VDREDTTYSY KARFTLAVGD NRVLDMASTY FDIRGVLDRG

D_51_ABA00026 APTHDVTTDR SQRLTLRFVP VDREDTTYSY KARFTLAVGD NRVLDMASTY FDIRGVLDRG

D_30_ABA00012 APTHDVTTDR SQRLTLRFVP VDREDTTYSY KARFTLAVGD NRVLDMASTY FDIRGVLDRG

D_29_BAJ22326 APTHDVTTDR SQRLTLRFVP VDREDTTYSY KARFTLAVGD NRVLDMASTY FDIRGVLDRG

D_56_ADM66117 APTHDVTTDR SQRLTLRFVP VDREDTTYSY KARFTLAVGD NRVLDMASTY FDIRGVLDRG

D_69_AFK92217 APTHDVTTDR SQRLTLRFVP VDREDTTYSY KARFTLAVGD NRVLDMASTY FDIRGVLDRG

D_15_BAJ22290 APTHDVTTDR SQRLTLRFVP VDREDTTYSY KARFTLAVGD NRVLDMASTY FDIRGVLDRG

B_35_AAP92351 APTHDVTTDR SQRLMLRFVP VDREDNTYSY KVRYTLAVGD NRVLDMASTF FDIRGVLDRG

B_11_ACZ06785 APTHDVTTDR SQRLMLRFVP VDREDNTYSY KVRYTLAVGD NRVLDMASTF FDIRGVLDRG

B_55_AIS92536 APTHDVTTDR SQRLMLRFVP VDREDNTYSY KVRYTLAVGD NRVLDMASTF FDIRGVLDRG

B_21_AAG21823 APTHDVTTDR SQRLMLRFVP VDREDNTYAY KVRYTLAVGD NRVLDMASTF FDIRGVLDRG

B_50_ABA00027 APTHDVTTDR SQRLMLRFVP VDREDNTYAY KVRYTLAVGD NRVLDMASTF FDIRGVLDRG

B_14_AAZ99996 APTHDVTTDR SQRLMLRFVP VDREDNTYSY KVRYTLAVGD NRVLDMASTF FDIRGVLDRG

B_34_AAW33485 APTHDVTTDR SQRLMLRFVP VDREDNTYSY KVRYTLAVGD NRVLDMASTF FDIRGVLDRG

B_79_BAW32492 APTHDVTTDR SQRLMLRFVP VDREDNTYSY KVRYTLAVGD NRVLDMASTF FDIRGVLDRG

B_68_AET87230 APTHDVTTDR SQRLMLRFVP VDREDNTYSY KVRYTLAVGD NRVLDMASTF FDIRGVLDRG

B_66_AET87148 APTHDVTTDR SQRLMLRFVP VDREDNTYSY KVRYTLAVGD NRVLDMASTF FDIRGVLDRG

E_4_AAD03660 APTHDVTTDR SQRLTLRFVP VDREDNTYSY KVRYTLAVGD NRVLDMASTY FDIRGVLDRG

B_16_AAW33444 APTHDVTTDR SQRLMLRFVP VDREDNTYSY KVRYTLAVGD NRVLDMASTF FDIRGVLDRG

A_61_AEK79922 APTHDVTTDR SQRLTLRFVP VDREDTAYSY KARFTLAVGD NRVLDMASSY FDIRGVLDRG

A_12_BAG48789 APTHDVTTDR SQRLTLRFVP VDREDTTYSY KARFTLAVGD NRVLDMASSY FDIRGVLDRG

A_18_AAZ99994 APTHDVTTDR SQRLTLRFVP VDREDTAYSY KARFTLAVGD NRVLDMASSY FDIRGVLDRG

A_31_CAO78638 APTHDVTTDR SQRLTLRFVP VDREDTAYSY KARFTLAVGD NRVLDMASSY FDIRGVLDRG

G_52_ABK35044 APTHDVTTDR SQRLTLRFVP VDREDTAYSY KVRYTLAVGD NRVLDMASTY FDIRGVLDRG

F_41_ACH90432 APTHDVTTDR SQRLTLRFVP VDREDTAYSY KVRFTLAVGD NRVLDMASTY FDIRGVLDRG

F_40_AMQ95234 APTHDVTTDR SQRLTLRFVP VDREETAYSY KVRFTLAVGD NRVLDMASTY FDIRGVLDRG

....|....| ....|....| ....|....| ....|....| ....|....| ....|....|

125 135 145 155 165 175

HexN1 PSFKPYSGTA YNSLAPKGAP NSSQWEQKE- ------TNGG G--------- ----------

B_3_AAW33168 PSFKPYSGTA YNSLAPKGAP NTSQWIVTT- --------NG D--------- --------NA

C_5_AAW65514 PTFKPYSGTA YNALAPKGAP NPCEWDEAAT ALEINLEEED DDNEDEVDE- --------QA

B_7_AAD03663 PSFKPYSGTA YNSLAPKGAP NTSQWIVTA- --------GE E--------- --------RA

C_2_AAO24095 PTFKPYSGTA YNALAPKGAP NSCEWEQTED SGRAVAEDEE EEDEDEEEEE EEQNAR--DQ

C_1_AAQ10553 PTFKPYSGTA YNALAPKGAP NSCEWEQEEP TQEMAEELED EEEAEEEEAE EEAEAPQADQ

C_57_ADM46153 PTFKPYSGTA YNALAPKGAP NSCEWDEDDT QVQVAAEDDQ DDDEEEEQLP Q-------QR

C_6_ADV03661 PTFKPYSGTA YNALAPKGAP NSCEWEQNET AQVDAQELDE EENEANEAQA RE------QE

D_37_ABA00016 PSFKPYSGTA YNSLAPKGAP NPSQWTTKE- ------KQNG GT-------- --------GA

D_13_ABA00000 PSFKPYSGTA YNSLAPKGAP NPSQWTTKE- ------KQTG VN-------- --------AG

D_70_AKI33526 PSFKPYSGTA YNSLAPKGAP NPSQWITKE- ------KQTG VN-------- --------AG

D_63_AEV92966 PSFKPYSGTA YNSLAPKGAP NPSQWTTKE- ------KQTG VN-------- --------AG

D_72_AGT78001 PSFKPYSGTA YNSLAPKGAP NPSQWTTKE- ------KQTG VN-------- --------AG

D_23_ABA00005 PSFKPYSGTA YNSLAPKGAP NPSQWEQKKT GV-------- ---------- --------GP

D_24_ABA00006 PSFKPYSGTA YNSLAPKGAP NSSQWEQAKA T--------- ---------- --------NA

D_39_ABA00018 PSFKPYSGTA YNSLAPKGAP NPSQWIAKE- ------KQNG PP-------- --------QT

D_43_ABA00020 PSFKPYSGTA YNSLAPKGAP NSSQWETKE- ------KQNG GS-------- --------GA

D_58_ADW95419 PSFKPYSGTA YNSLAPKGAP NSSQWEQKK- ------ANGG A--------- ----------

D_33_ABA00014 PSFKPYSGTA YNSLAPKGAP NSSQWEQKK- ------ATGG A--------- ----------

D_38_ABA00017 PSFKPYSGTA YNSLAPKGAP NSSQWDAKE- ------KNGG A--------- ----------

D_36_ACY04472 PSFKPYSGTA YNSLAPKGAP NSSQWTDKE- ------RQNG G--------- --------QP

D_49_ABA00025 PSFKPYSGTA YNSLAPKGAP NSSQWDAKE- ------NNGQ G--------- ----------

D_8_AAZ99998 PSFKPYSGTA YNSLAPKGAP NPSQWEQAK- ------TGAG V--------- --------DQ

D_46_ABA00023 PSFKPYSGTA YNSLAPKGAP NSSQWEQKKA NG-------- ---------- --------GP

D_47_ABA00024 PSFKPYSGTA YNSLAPKGAP NPSQWDAQE- ------NNAG D--------- ----------

D_44_ABA00021 PSFKPYSGTA YNSLAPKGAP NPSQWEQKK- --------NG G--------- --------GA

D_48_ABO61301 PSFKPYSGTA YNSLAPKGAP NPSQWEEKK- ------NGGG S--------- --------DA

D_22_ABA00004 PSFKPYSGTA YNSLAPKGAP NSSQWAQKKT G--------- ---------- --------ED

D_53_BAJ46350 PSFKPYSGTA YNSLAPKGAP NSSQWAQKKT G--------- ---------- --------ED

D_45_ABA00022 PSFKPYSGTA YNSLAPKSAP NPSQWDAKE- ------KEGV A--------- --------QT

D_26_ABA00008 PSFKPYSGTA YNSLAPKGAP NPSQWETKEK QG----TTGG V--------- --------QQ

D_32_ABA00013 PSFKPYSGTA YNSLAPKGAP NSSQWAAKDA NT-------- ---------- --------AD

D_73_APD28385 PSFKPYSGTA YNSLAPKGAP NSSQWVAKDT NA-------- ---------- --------TD

D_67_BAL63187 PSFKPYSGTA YNSLAPKGAP NSSQWLAKDT NA-------- ---------- --------GD

D_9_BAE66671 PSFKPYSGTA YNSLAPKGAP NSSQWITKDT NA-------- ---------- --------GN

D_54_YP_0030386 PSFKPYSGTA YNSLAPKGAP NSSQWITKQT NA-------- ---------- --------GN

D_17_ADY18429 PSFKPYSGTA YNSLAPKGAP NPSQWVAKEN ---------- ---------- --------GQ

D_10_AAZ99999 PSFKPYSGTA YNSLAPKSAP NPSQWTANE- ------KQTG G--------- ---------Q

D_65_BAL41721 PSFKPYSGTA YNSLAPKSAP NPSQWTANE- ------KQTG G--------- ---------Q

D_62_AEL78842 PSFKPYSGTA YNSLAPKGAP NSSQWDAEE- ------KKDT Q---G----- --------NE

D_25_ABA00007 PSFKPYSGTA YNSLAPKGAP NSSQWEEKKS G--------- ---------- --------AR

D_59_AEI91289 PSFKPYSGTA YNSLAPKGAP NSSQWEEKKN G--------- ---------- --------AG

D_19_ABA00002 PSFKPYSGTA YNSLAPKGAP NSSQWDAQE- ------KNGQ GG-------- --------ND

D_64_ABN10535 PSFKPYSGTA YNSLAPKGAP NSSQWDAQE- ------KNGQ GG-------- --------ND

D_27_ABA00009 PSFKPYSGTA YNSLAPKGAP NSSQWTAK-- ---------- ---------- --------EN

D_28_ACQ91158 PSFKPYSGTA YNSLAPKGAP NSSQWDAQE- ------KSGQ G--------- --------SD

D_20_ABA00003 PSFKPYSGTA YNSLAPKGAP NSSQWLAKDT NA-------- ---------- --------AG

D_60_AEK87026 PSFKPYSGTA YNSLAPKGAP NSSQWEQKK- ------TTGG G--------- ----------

D_71_AGT76762 PSFKPYSGTA YNSLAPKGAP NSSQWEQKK- ------TTGG G--------- ----------

D_42_ABA00019 PSFKPYSGTA YNSLAPKGAP NSSQWADKE- ------RVNG G--------- --------GN

D_51_ABA00026 PSFKPYSGTA YNSLAPKGAP NSSQWEQKK- ------TTGG G--------- ----------

D_30_ABA00012 PSFKPYSGTA YNSLAPKGAP NSSQWEQKKA N--------- ---------- --------AG

D_29_BAJ22326 PSFKPYSGTA YNSLAPKGAP NSSQWEQKKA N--------- ---------- --------AG

D_56_ADM66117 PSFKPYSGTA YNSLAPKGAP NSSQWEQKKA N--------- ---------- --------AG

D_69_AFK92217 PSFKPYSGTA YNSLAPKGAP NSSQWEQKKA N--------- ---------- --------AG

D_15_BAJ22290 PSFKPYSGTA YNSLAPKGAP NSSQWEQKKA N--------- ---------- --------AG

B_35_AAP92351 PSFKPYSGTA YNSLAPKGAP NASQWIAKGV PTAAAAGNGE EE-HE----- --------TE

B_11_ACZ06785 PSFKPYSGTA YNSLAPKGAP NTSQWIAEGV ------KNGE ERVTE----- --------EE

B_55_AIS92536 PSFKPYSGTA YNSLAPKGAP NTSQWIAEGV ------KNGE ERVTE----- --------EE

B_21_AAG21823 PSFKPYSGTA YNSLAPKGAP NTSQWIAEGV KK----EDGG SD-EE----- --------EE

B_50_ABA00027 PSFKPYSGTA YNSLAPKGAP NTSQWLNKG- ------DEED G--------- --------ED

B_14_AAZ99996 PSFKPYSGTA YNSLAPKGAP NASQWLDKGV ETTEERQNED G--------- --------EN

B_34_AAW33485 PSFKPYSGTA YNSLAPKGAP NASQWLDKGV TSTGLVDDGN TD-DG----- --------EE

B_79_BAW32492 PSFKPYSGTA YNSLAPKGAP NASQWLDKGV TSTGLVDDGN DD-DG----- --------EE

B_68_AET87230 PSFKPYSGTA YNSLAPKGAP NTSQWIVTTN ---------- ---AD----- --------QT

B_66_AET87148 PSFKPYSGTA YNSLAPKGAP NTSQWIVTT- ---------- ---------- --------GE

E_4_AAD03660 PSFKPYSGTA YNSLAPKGAP NTCQW----- ---------- ---------- --------KD

B_16_AAW33444 PSFKPYSGTA YNSLAPKGAP NTCQW----- ---------- ---------- --------KD

A_61_AEK79922 PSFKPYSGTA YNSLAPKGAP NASQW----- ---------- ---------- ---------S

A_12_BAG48789 PSFKPYSGTA YNSLAPKGAP NASQW----- ---------- ---------- ---------S

A_18_AAZ99994 PSFKPYSGTA YNSLAPKGAP NASQWL---- ---------- ---------- --------TT

A_31_CAO78638 PSFKPYSGTA YNSLAPKGAP NASQWLT--- ---------- ---------- --------TN

G_52_ABK35044 PSFKPYSGTA YNSLAPKGAP NSVEW----- ---------- ---------- ---------P

F_41_ACH90432 PSFKPYSGTA YNSLAPKTAP NPCEW----- ---------- ---------- ---------K

F_40_AMQ95234 PSFKPYSGTA YNSLAPKGAP NPSQW----- ---------- ---------- ---------T

....|....| ....|....| ....|....| ....|....| ....|....| ....|....|

185 195 205 215 225 235

HexN1 DKTE--THTF GVAAM---GG E-NITKK-GL QIGTDTT--E NENKP-IYAD KTYQPEPQVG

B_3_AAW33168 VTTT--TNTF GIASM---KG D-NITKE-GL QIGKDITTTE GEEKP-IYAD KTYQPEPQVG

C_5_AAW65514 EQQK--THVF GQAPY---SG I-NITKE-GI QIGVEGQ--- ---TP-KYAD KTFQPEPQIG

B_7_AAD03663 VTTT--TNTF GIASM---KG D-NITKE-GL EIGKDIT--- ADNKP-IYAD KTYQPEPQVG

C_2_AAO24095 ATKK--THVY AQAPL---SG E-TITKS-GL QIGSNNA--E TQAKP-VYAD PSYQPEPQIG

C_1_AAQ10553 KVKK--THVY AQAPL---AG E-KITAN-GL QIVSDTQ--- TEGNP-VFAD PTYQPEPQVG

C_57_ADM46153 NGKK--THVY AQAPF---AG E-AINKN-GL QIGTNGA--A TEGNKEIYAD KTYQPEPQIG

C_6_ADV03661 QAKK--THVY AQAPL---SG I-KITKE-GL QIGTADATVA GAGKE-IFAD KTFQPEPQVG

D_37_ABA00016 EKDV--TKTF GLAAM---GG S-NISKD-GL QIGTDKT--A NAEKP-IYAD KTFQPEPQVG

D_13_ABA00000 DKEV--TKTF GIAAM---GG S-NISEN-GL QIGTDTT--A DGTKP-IYAD KTFQPEPQVG

D_70_AKI33526 DKDV--TKTF GIAAM---GG S-NISKD-GL QIGTDTT--A AAAKP-IYAD KTFQPEPQVG

D_63_AEV92966 DKEV--TKTF GLAAM---GG S-NISKD-GL QIGTDTT--P DAVKP-IYAD KTYQPEPQVG

D_72_AGT78001 DKEV--TKTF GLAAM---GG S-NISKD-GL QIGTDTT--A DAVKP-IYAD KTYQPEPQVG

D_23_ABA00005 EAME--KHTF GMAAM---AG E-AITNK-GL QIGVDTT--D GKQDP-IYAN QLYQPEPQVG

D_24_ABA00006 GQKE--THTF GVAAM---GG E-DITVK-GL QIGTDET--K EDGEDEIFAD QTFQPEPQVG

D_39_ABA00018 EKNV--TKTF GVAAM---GG L-DITNE-GL QIGVEEI--N DVEEE-VFAD KTFQPEPQVG

D_43_ABA00020 QIEKNVTKTF GVAAM---GG L-DITDE-GL QIGVEEI--N NVEEE-VFAD KIFQPEPQVG

D_58_ADW95419 DEME--THTF GVAAM---GG K-NITDK-GL QIGTDET--K EDDEDEIYAD KTFQPEPQVG

D_33_ABA00014 DAKE--THTF GVAAM---GG L-NITDK-GL QIGIDED--N VDGDDEIYAD KTFQPEPQVG

D_38_ABA00017 ETTK--TYTY GVAAM---GG L-DITDK-GL QIGTDET--K EDNNE-IFAD KTFQPEPQVG

D_36_ACY04472 PTTKDVTKTF GVAAR---GG L-HITDK-GL QIGEDEN--N EDGEEEIYAD KTFQPEPQVG

D_49_ABA00025 -EAK--THTY GVAAM---GG Y-NITKD-GL QIGIDENKEE DEEGREIFAV KSYQPEPQVG

D_8_AAZ99998 NQKE--TRTY GVAAT---GG Y-NITKE-GL QIGIDET--K EDPNNKIYAD KTFQPEPQIG

D_46_ABA00023 NEME--THTF GVAAM---GG E-NITKD-GL QIGTETT--A ENQNKEIFAD KTFQPEPQVG

D_47_ABA00024 --AK--THTY GVASM---PG I-DITDK-GL QIGIDANKDE DEGNE-IFAD KTFQPEPQVG

D_44_ABA00021 DQME--TRTY GVAAM---GG I-DIDKN-GL QIGVEQT--A DNGQKEIYAD KLFQPEPQIG

D_48_ABO61301 NQMQ--THTF GVAAM---GG I-EITAK-GL QIGIDATKEE DNGKE-IYAD KTFQPEPQIG

D_22_ABA00004 NQTE--TRTF GVAAM---GG I-LIDKN-GL QIGTDET--K PNNKE-VYAD KTFQPEPQIG

D_53_BAJ46350 NQTE--TRTF GVAAM---GG I-LIDKN-GL QIGTDET--K PDNKE-IYAD KTFQPEPQKG

D_45_ABA00022 EKNV--LKTF GVAAT---GG F-NITDQ-GL LLGTEET--A ENVKKDIYAE KTFQPEPQVG

D_26_ABA00008 EKDV--TKTF GVAAT---GG I-NITNQ-GL LLGTDET--A ENGKKDIYAD KTFQPEPQVG

D_32_ABA00013 QTVK--THTH GVAAM---GG T-DITAK-GL QIGVDTT--E NNAGP-IYAN EIYQPEPQIG

D_73_APD28385 QALK--THTH GVAAM---GG T-DITAK-GL QIGVDTT--E NKNEP-IYAN EIYQPEPQIG

D_67_BAL63187 QALK--THTH GVAAM---GG T-DITAK-GL QIGVDTT--E NKNEP-IYAN EIYQPEPQVG

D_9_BAE66671 ETTK--THTH GVAAM---GG A-DITIK-GL QIGVDRT--E NKNEP-IYAN EIYQPEPQVG

D_54_YP_0030386 ETTK--THTY GVAAM---GG A-DITIK-GL QIGVDKT--E NKNEP-IYAN EIYQPEPQVG

D_17_ADY18429 GTDK--THTY GSAAM---GG S-NITIE-GL VIGTDEK--A EDGKKDIFAN KLYQPEPQVG

D_10_AAZ99999 PKSV--TQTF GSAPM---GG S-NITIE-GL VIGTKEE--E GNATEEIFAD KTFQPEPQVG

D_65_BAL41721 PKSV--TQTF GSAPM---GG S-NITIE-GL VIGTKEE--E GNATEEIFAD KTFQPEPQVG

D_62_AEL78842 MVTK--THTY GVAPM---AG T-NITKK-GL LLGTDET--A EAGKKDIYAD ETYQPEPQVG

D_25_ABA00007 NQTE--THTF GVAPM---GG T-NITIS-GL QIGTEEE--D GNPTKEIFAD KTFQPEPQIG

D_59_AEI91289 NQTE--THTY GVAPM---GG T-NITIN-GL QIGTEEE--D GNPTKEIFAD KTFQPEPQIG

D_19_ABA00002 MVTK--THTF GVAAM---GG T-NITNQ-GL LIGTEET--A HNPPKEIFAD KLFQPEPQVG

D_64_ABN10535 MVTK--THTF GVAAM---GG T-NITNQ-GL LIGTEET--A DNPPKEIFAD KLFQPEPQVG

D_27_ABA00009 QKDV--TKTF GVAPM---GG I-NISKD-GL QIGVEET--V DKQEKEIYAD KSFQPEPPVG

D_28_ACQ91158 MVTK--THTF GVAAM---GG E-NITKN-GL QIGTEIT--A DNQKKEIFAN KTYQPEPQVG

D_20_ABA00003 QPDK--THTY GVAAM---GG E-DITEK-GL QIGIDET--K EENNK-IFAN EIYQPEPHVG

D_60_AEK87026 NDME--THTY GVAAM---GG E-DITEK-GL QIGIDET--K EENNK-IFAD KTFQPEPQVG

D_71_AGT76762 NDME--THTY GVAAM---GG E-DITEK-GL QIGIDET--K EENNK-IFAD KTFQPEPQVG

D_42_ABA00019 TKDV--TKTF GVAAM---GG E-DITEK-GL KIGTDPT--- -ANEP-IFAD KNFQPEPQVG

D_51_ABA00026 NDME--THTF GVAAM---GG E-NITKD-GL QIGTDTT--A DADKP-IYAD KTFQPEPQVG

D_30_ABA00012 DQKE--THTY GVAPM---GG E-NITIS-GL QIGTDTT--N GKQDP-IYAN KLYQPEPQVG

D_29_BAJ22326 DQKE--THTY GVAPM---GG E-NITIS-GL QIGTDTT--N GKQDP-IYAN KLYQPEPQVG

D_56_ADM66117 EQKE--THTY GVAPM---GG E-NITIS-GL QIGTDTT--N GKQDP-IYAN KLYQPEPQVG

D_69_AFK92217 DQKE--THTY GVAPM---GG E-NITIS-GL QIGTDTT--N GKQDP-IYAN KLYQPEPQVG

D_15_BAJ22290 DQKE--THTY GVAPM---GG E-NITIS-GL QIGTDTT--N GKQDP-IYAN KLYQPEPQVG

B_35_AAP92351 EKTA--TYTF ANAPV---KA EAQITKE-GL PIGLEISA-E NESKP-IYAD KLYQPEPQVG

B_11_ACZ06785 NNTT--TYTF GNAPV---KA EAEITKE-GL PIGLKVS--D EESKP-IYAD KTYQPEPQLG

B_55_AIS92536 NNTT--TYTF GNAPV---KA EAEITKE-GL PIGLKVS--D EESKP-IYAD KTYQPEPQLG

B_21_AAG21823 KNLT--TYTF GNAPVKAEGG --DITKDKGL PIGSEIT--D GEAKP-IYAD KLYQPEPQVG

B_50_ABA00027 DQQA--TYTF GNAPV---KA EAEITKE-GL PIGLEVPS-E GGPKP-IYAD KLYQPEPQVG

B_14_AAZ99996 DEKA--TYTF GNAPV---KA DADITKD-GL PIGLEVPA-E GDPKP-IYAN KLYQPEPQVG

B_34_AAW33485 AKKA--TYTF GNAPV---KA EAEITKD-GL PVGLEVS--T EGPKP-IYAD KLYQPEPQVG

B_79_BAW32492 AKKA--TYTF GNAPV---KA EAEITKD-GL PVGLEVS--T EGPKP-IYAD KLYQPEPQVG

B_68_AET87230 TTTT--TNTF GIAAM---KG D-NITKE-GL QIGKDITTTE GEEKP-IYAD KTYQPEPQVG

B_66_AET87148 DNAT--TYTF GIAST---KG D-NITKE-GL EIGKDIT--- ADNKP-IYAD KTYQPEPQVG

E_4_AAD03660 ANSK--MHTF GVAAMPGVTG K-KIEAD-GL PIRIDST--S GTDTV-IYAD KTFQPEPQVG

B_16_AAW33444 SDSK--MHTF GVAAMPGVTG K-KIEAD-GL PIGIDST--S GTDTV-IYAD KTFQPEPQVG

A_61_AEK79922 DNNK--VNTF GQAPY---LS D-SITAD-GI KVGTDTA--- QAGAA-VYAD KKYQPEPQVG

A_12_BAG48789 DNAK--LNTF AQAPY---LS D-TITAADGI KVGTDTA--- QAGAA-VYAN KTYQPEPQVG

A_18_AAZ99994 NDNK--SHTF AQAPY---IG S-SITKD-GI QVGTNTA--- NPPQP-VYAD KTYQPEPQVG

A_31_CAO78638 NGNK--THTF AQAPY---IG D-SISKD-GI QVGTNTA--- NPQQA-VYAD KTYQPEPQVG

G_52_ABK35044 DNTK--TKVR AQAPF---VS D-EITKD-GI KVGTDTA--- -TTQQSIYAD KTYQPEPQVG

F_41_ACH90432 DNNK--IKVR GQAPF---IG T-NINKDNGI QIGTDTT--- --NQP-IYAD KTYQPEPQVG

F_40_AMQ95234 NQNK--TNSF GQAPY---IG Q-KITNQ-GV QVGLDSN--- --NRD-VFAD KTYQPEPQVG

....|....| ....|....| ....|....| ....|....| ....|....| ....|....|

245 255 265 275 285 295

HexN1 EENWQETE-- -NFYGGRALK KETKMKPCYG SFARPTNEKG GQAK-FKT-- --GE-NGQPT

B_3_AAW33168 EESWTDTDGT NEKFGGRALK PATNMKPCYG SFARPTNIKG GQAK-NRKVK PTTE-GGVET

C_5_AAW65514 ESQWYETE-- INHAAGRVLK KTTPMKPCYG SYAKPTNENG GQGI-LVK-- -----QQNGK

B_7_AAD03663 EESWTDTDGT NEKFGGRALK PATKMKPCYG SFARPTNIKG GQAKNRKV-- --KPTEGDVE

C_2_AAO24095 ESQWNEAD-- ANAAGGRVLK KTTPMKPCYG SYARPTNPFG GQSV-LVP-- -----DEKGV

C_1_AAQ10553 ESQWNEAE-- ATASGGRVLK KTTPMKPCYG SYARPTNKNG GQGI-LVA-- -----NNQGA

C_57_ADM46153 ESQWNEAE-- SSVAGGRVLK KTTPMKPCYG SYARPTNSNG GQGV-MVE-- -----QN-GK

C_6_ADV03661 ESQWNEAD-- ATAAGGRVLK KTTPMKPCYG SYARPTNSNG GQGV-MVE-- -----QN-GK

D_37_ABA00016 EENWQDND-- -EYYGGRALK KDTKMKPCYG SFAKPTNKEG GQAK-LKE-- --TP-NGTDP

D_13_ABA00000 EENWQDND-- -EYYGGRALK KDTKMKPCYG SFAKPTNKEG GQAK-LKE-- --TP-NGADP

D_70_AKI33526 EENWQDND-- -EYYGGRALK KDTKMKPCYG SFAKPTNKEG GQAK-LKE-- --TP-NGADP

D_63_AEV92966 EENWQDND-- -EYYGGRALK KDTKMKPCYG SFAKPTNKEG GQAK-LKE-- --TP-NGADP

D_72_AGT78001 EENWQDND-- -EYYGGRALK KDTKMKPCYG SFAKPTNKEG GQAK-LKE-- --TP-NGADP

D_23_ABA00005 EDSWNDDV-- APSYGGRALK KETKMKPCYG SFARPTNEKG GQAK-LRD-- -----PEKSQ

D_24_ABA00006 EQNWQETF-- -VFYGGRALK KETKMKPCYG SYARPTNEKG GQAK-FTL-- --DE-KGQPT

D_39_ABA00018 EENWHETF-- -NFYGGRTLK KETKMKPCYG SFARPTNIKG GQAK-LKT-- --GE-NVDPT

D_43_ABA00020 EENWQETF-- -NFYGGRALK KDTKMKPCYG SFARPTNEKG GQAK-LKT-- --GE-NVDPT

D_58_ADW95419 EENWKETF-- -VYYGGRAIK KDTKMKPCYG SYARPTNEKG GQAK-FLN-- --GE-NGQPS

D_33_ABA00014 EENWKESF-- -NFYGGRAIK KDTKMKPCYG SFARPTNVKG GQAK-LKT-- --GE-DIDPN

D_38_ABA00017 EETWQENE-- -VFYGGRALK KETKMKPCYG SFARPTNEKG GQAK-FLN-- --DD-QGLPS

D_36_ACY04472 EENWQDTD-- -VFYGGRALK KETKMKPCYG SFARPTNEKG GQAK-FLN-- --GE-NGQPS

D_49_ABA00025 EENWQNTE-- -NFYGGRALK KETKMKPCYG SFARPTNDKG GQAV-FKT-- --GE-NGKPT

D_8_AAZ99998 ENNWQDTN-- -VFYGGRALK KETKMKPCYG SFARPTNKKG GQAK-VLT-- --TE-DGQPT

D_46_ABA00023 EENWQETF-- -NFYGGRALK KETKMKPCYG SFARPMNEKG GQAK-FLT-- --KE-NGELT

D_47_ABA00024 EENWQESE-- -NFYGGRALK KETKMKPCYG SFARPTNEKG GQAK-FKT-- --PDKEGEQP

D_44_ABA00021 EENWHETF-- -VYYGGRALK KDTKMKPCYG SFARPTNEKG GQAK-PKT-- --GE-GVDPT

D_48_ABO61301 EENWQDSD-- -NYYGGRAIK KETKMKPCYG SFARPTNEKG GQAK-FKT-- --PEKEGEEP

D_22_ABA00004 EENWQDGD-- -VFYGGRTIK KETKMKPCYG SFARPTNEKG GQAK-FKT-- --NA-EGQPT

D_53_BAJ46350 EENWQDGD-- -VFYGGRTIK KETKMKPCYG SFARPTNEKG GQAK-FKT-- --NA-EGQPT

D_45_ABA00022 EENWQESE-- -AFYGGRAIK KDTKMKPCYG SFARPTNEKG GQAK-FKT-- --L--DGQVT

D_26_ABA00008 EENWQENE-- -AFYGGRALK KDTKMKPCYG SFARPTNEKG GQAK-FKP-- --VN-EGEQP

D_32_ABA00013 EENLQDVE-- -NYYGGRALK KETKMKPCYG SFARPTNEKG GQAK-FIT-- --GE-DGQPT

D_73_APD28385 EENLQDVE-- -NFYGGRTLK KETKMKPCYG SFARPTNEKG GQAK-FLT-- --DG-DGQLT

D_67_BAL63187 EENLQDVE-- -NFYGGRALK KETKMKPCYG SFARPTNEKG GQAK-FLT-- --DG-DGQLT

D_9_BAE66671 EENLQDVE-- -NYYGGRALK KETKMKPCYG SFARPTNEKG GQAVKFLT-- --DG-DGQLT

D_54_YP_0030386 EENLQDVE-- -NYYGGRALK KETKMKPCYG SFARPTNEKG GQAV-FKT-- --GN-DGQPT

D_17_ADY18429 EENWQESE-- -AFYGGRALK KDTKMKPCYG SFARPTNEKG GQAK-FKP-- --VE-EGQQP

D_10_AAZ99999 EENWQETE-- -AFYGGRALK KDTKMKPCYG SFARPTNEKG GQAK-LKL-- --NA-QGQPT

D_65_BAL41721 EENWQETE-- -AFYGGRALK KDTKMKPCYG SFARPTNEKG GQAK-LKL-- --ND-QGQPT

D_62_AEL78842 EENWQENE-- -AFYGGRALK KETKMKPCYG SFARPTNEKG GQAK-FKP-- --VE-EGQQP

D_25_ABA00007 EENWQDTE-- -NFYGGRALK KDTKMKPCYG SFARPTNEKG GQAK-LKL-- --DA-QGQST

D_59_AEI91289 EENWQDTE-- -NFYGGRALK KDTKMKPCYG SFARPTNEKG GQAK-LKL-- --DA-QGQPT

D_19_ABA00002 EENWQDTN-- -AFYGGRALK KETKMKPCYG SYARPTNTSG GQAK-LKT-- --GD-NIDPT

D_64_ABN10535 EENWQDSN-- -AFYGGRALK KETKMKPCYG SYARPTNTSG GQAK-LKT-- --GD-NIDPT

D_27_ABA00009 KENWQESE-- -AFYGGRALK KDTKMKPCYG SFARPTNEKG GQAK-FKA-- --PAVEGEQP

D_28_ACQ91158 EENWQENE-- -VFYGGRALK KETKMKPCYG SFARPTNENG GQAK-FKT-- --PA-EGQEP

D_20_ABA00003 EENWQETF-- -VFYGGRALK KDTKMKPCFG SFARPYYEKG GQAK-FVL-- --DQ-EGKPT

D_60_AEK87026 EENWQETF-- -VFYGGRALK KDTKMKPCYG SFARPTNEKG GQAK-FVL-- --DQ-EGKPT

D_71_AGT76762 EENWQETF-- -VFYGGRALK KDTKMKPCYG SFARPTNEKG GQAK-FVL-- --DQ-EGKPT

D_42_ABA00019 EENWQETF-- -VFYGGRALK KETKMKPCYG SFARPTNEKG GQAK-FII-- --GD-NGQPT

D_51_ABA00026 EENWQETF-- -NFYGGRALK KDTKMKPCYG SFARPTNEKG GQAK-LKN-- --GP-DGKPT

D_30_ABA00012 EENWQETE-- -AFYGGRALK KETKMKPCYG SFARPTNEKG GQAK-LRD-- -----PEKSQ

D_29_BAJ22326 EENWQETE-- -AFYGGRALK KETKMKPCYG SFARPTNEKG GQAK-LRD-- -----PEKSQ

D_56_ADM66117 EENWQETE-- -AFYGGRALK KETKMKPCYG SFARPTNEKG GQAK-LRD-- -----PEKSQ

D_69_AFK92217 EENWQETE-- -AFYGGRALK KETKMKPCYG SFARPTNEKG GQAK-LRD-- -----PEKSQ

D_15_BAJ22290 EENWQETE-- -AFYGGRALK KETKMKPCYG SFARPTNEKG GQAK-LRD-- -----PEKSQ

B_35_AAP92351 DETWTDLDGK TEEYGGRALK PTTNMKPCYG SYAKPTNLKG GQAK-PKN-- --SE-PSSEK

B_11_ACZ06785 DETWTDLDGK TEKYGGRALK PDTKMKPCYG SFAKPTTVKG GQAK-PKT-- --TE-QPNQK

B_55_AIS92536 DETWTDLDGK TEKYGGRALK PDTKMKPCYG SFAKPTTVKG GQAK-PKT-- --TE-QPNQK

B_21_AAG21823 DETWTDTDGT TEKYGGRALK PETKMKPCYG SFAKPTNVKG GQAK-QKT-- --TEQPQNQQ

B_50_ABA00027 EESWTDTDGT DEKYGGRALK PETKMKPCYG SFAKPTNVKG GQAK-VKK-- -----EEEGK

B_14_AAZ99996 QESWTDTDGT EEKYGGRVLK PDTKMKPCYG SFAKPTNVKG GQAK-VKT-- -----EEAGN

B_34_AAW33485 DETWTDLDGK TEEYGGRVLK PETKMKPCYG SFAKPTNIKG GQAK-VKP-- --KEDDGTNN

B_79_BAW32492 DETWTDLDGK TEEYGGRVLK PETKMKPCYG SFAKPTNIKG GQAK-VKP-- --KEDDGTNN

B_68_AET87230 EESWTDTDGT NEKFGGRALK PATNMKPCYG SFARPTNIKG GQAK-NRKVK PTTE-GGVET

B_66_AET87148 EESWTDIDGT NEKFGGRALK PATKMKPCYG SFARPTNIKG GQAKNRKV-- --TPTEGDVE

E_4_AAD03660 NDSWVDTNDA EEKYGGRALK DTTNMNPCYG SFAKPTNKEG GQAN-LKD-- --SE-TAATT

B_16_AAW33444 NASWVDANGT EEKYGGRALK DTTKMKPCYG SFAKPTNKEG GQAN-LKD-- --SE-TAATT

A_61_AEK79922 ASEWNTSI-T NVKAGGRALK QTTAMQPCYG SYARPTNEKG GQTK------ ----------

A_12_BAG48789 PSEWNTSI-E NVKAGGRALK QTTAMQPCYG SYARPTNEHG GQSK------ ----------

A_18_AAZ99994 ESQWNAPLPN NAKAAGRVLK NTTPMYPCYG SYARATNENG GQSK------ ----------

A_31_CAO78638 ESQWNASVSN NAKAAGRVLK STTPMYPCYG SYARATNEKG GQSK------ ----------

G_52_ABK35044 ETQWNSDVGT NDKVAGRVLK KTTPMYPCYG SYARPTNEKG GQAK------ ----------

F_41_ACH90432 QTQWNSEVGA AQKVAGRVLK DTTPMLPCYG SYAKPTNEKG GQAS-LIT-- -----NGTDQ

F_40_AMQ95234 QTQWNINP-- MQNAAGRILK QTTPMQPCYG SYARPTNEKG GQAKLVKN-- -----DDNQT

....|....| ....|....| ....|....| ....|....| ....|....| ....|....|

305 315 325 335 345 355

HexN1 -KDFDIDMAF FDTPGGTLTG N-----TEYK ADIVMYTENV NLETPDTHVV YKPGKEDD--

B_3_AAW33168 -EEPDIDMEF FDGRDAVA-- ------GALA PEIVLYTENV NLETPDSHVV YKPETSN---

C_5_AAW65514 -LESQVEMQF FSTTEAAAGN G-----DNLT PKVVLYSEDV DIETPDTHIS YMPTIKEG--

B_7_AAD03663 TEEPDIDMEF FDGREAA--- ------DAFS PEIVLYTENV NLETPDSHVV YKPGTSDD--

C_2_AAO24095 -PLPKVDLQF FSNTTSLNDR QG----NATK PKVVLYSEDV NMETPDTHLS YKPGKGDE--

C_1_AAQ10553 -LESKVEMQF FAPSGTAMNE R-----NAVQ PSIVLYSEDV NMETPDTHIS YKPSKTDE--

C_57_ADM46153 -LESQVEMQF FSTSVNAMNE A-----NAIQ PKLVLYSEDV NMETPDTHLS YKPGKSDD--

C_6_ADV03661 -LESQVEMQF FSTSTNATNE V-----NNIQ PTVVLYSEDV NMETPDTHLS YKPKMGDK--

D_37_ABA00016 --QYDVDMAF FDSS------ ------TINI PDVVLYTENV DLETPDTHVV YKPGKEDD--

D_13_ABA00000 --QYDVDMAF FDST------ ------TINI PDVVLYTENV DLETPDTHVV YKPGKEDD--

D_70_AKI33526 --QYDVDMAF FDST------ ------TINI PDVVLYTENV DLETPDTHVV YKPGKEDD--

D_63_AEV92966 --QYDVDMAF FDST------ ------TINI PDVVLYTENV DLETPDTHVV YKPGKEDE--

D_72_AGT78001 --QYDVDMAF FDST------ ------TINI PDVVLYTENV DLETPDTHVV YKPGKEDE--

D_23_ABA00005 -EDFDIDMAF FDSN------ ------TINT PDVVLYTENV NLETPDSHVV YKAGTSDE--

D_24_ABA00006 -KIPDITMDF FDSPQDDTSG ------VTNK PDIVMYAENV NLEAPDTHVV YKPGKDDS--

D_39_ABA00018 -KDFDIDMAF FDLKQTDTGT ------TQNQ PDIVMYTENV NLETPDTHVV YKPGKEDA--

D_43_ABA00020 -KDFDIDMAF FDLKQTDTGT ------TQNQ PDIVMYTENV NLETPDTHVV YKPGKEDA--

D_58_ADW95419 -KEQDITMAF FDLRQADAGG ------NKNQ ADVVMYAENI NLETPDTHVV YKPGKEDA--

D_33_ABA00014 -KDFDIDMAF FDLKQADTGG ------NNNQ ADVVMYTENI HLETPDTHVV YKPGKEDA--

D_38_ABA00017 -KEQDITMAF FDSPQADTSG ------VDNK PDMVMYTENV YLETPDTHVV FKPGKEDD--

D_36_ACY04472 -KDQDITLAF FDLKQNDTGT ------TQNQ PDVVMYTENV YLETPDTHVV YKPGKEDT--

D_49_ABA00025 -EELDIDLAF FDLRQNDTGG ------NNNQ PDMIMYAENV NLETPDTHVV YKPGTSDD--

D_8_AAZ99998 -ENFDIDLAF FDIPQAGGN- ------DNLD PDMILYAENV NLETPDTHVV YKPGKDDA--

D_46_ABA00023 -EDQDIDLNF FDINNPDTGG ------VANQ PDIIMYAENV NLETPDTHVV YKSGKEDD--

D_47_ABA00024 -KEYDIDMNF FDIPNTGTGG NGTN--VNNK PDIVLYAENM NLETPDTHVV YKPGKEDD--

D_44_ABA00021 -KDFDIDLAF FDIPQNGVQ- ------DNHD PDMIMYAENV NLETPDTHIV YKPGKEDE--

D_48_ABO61301 -KELDIDLNF FDIPSTGTGG NGTN--VNFK PDMIMYAENV NLETPDTHIV YKPGKEDA--

D_22_ABA00004 -EELDIDLNF FDINGGAGD- ------NEFN PDMVMYAENM NLETPDTHVV YKPGTSDD--

D_53_BAJ46350 -EELDIDLNF FDINGGAGD- ------NEFN PDMVMYAENM NLETPDTHVV YKPGTSDD--

D_45_ABA00022 -KDPDIDFAY FDVPGGKAPT GSSLP-EEYK ADIILYTENV NLETPDTHIV YKPGKEDD--

D_26_ABA00008 -KDLDIDFAY FDVPGGSPPA GGSG--EEYK ADIILYTENV NLETPDTHVV YKPGTSDN--

D_32_ABA00013 -KNHDITMNF FDTPGGTIGQ A-----DELE ADIVMYAENV HLETPDTHVV YKPGTSDE--

D_73_APD28385 -KNHDITMNF FDTPGGTVGQ D-----TELE ADIVMYAENV HLETPDTHVV YKPGTSDE--

D_67_BAL63187 -KNHDITMNF FDTPGGTVGQ D-----TELE ADIVMYAENV HLETPDTHVV YKPGTSDE--

D_9_BAE66671 -KNHDITMNF FDTPGDTNAE D-----TELE ADIVMYTENV NMETPDTHVV YKPGPLED--

D_54_YP_0030386 -TEHDITMAF FDTPGDTNAE D-----TELE ADIVMYTENV NLETPDTHVV YKPGPLED--

D_17_ADY18429 -KDYDIDLAF FDTPGGTITG GTD---EEYK ADIVLYTENV NLETPDTHVV YKPGKEDD--

D_10_AAZ99999 -KDYDIDLAF FDTPGGTPPT GSGQQ-EEYK ADIIMYTENV NLETPDTHVV YKPGKEDE--

D_65_BAL41721 -KDYDIDLAF FDTPGGTPPT GSGQQ-EEYK ADIVMYTENV NLETPDTHVV YKPGKEDE--

D_62_AEL78842 -KDLDITLAF FDTPGGTLNG SGT---EEYK ADIVMYTENV NLETPDTHVV YKPGTSDD--

D_25_ABA00007 -KDYDIDLAF FDSPGGNTTA GGQ---EELK ADIVMYTENA YLETPDTHVV YKPGTSDD--

D_59_AEI91289 -KDYDIDLAF FDSPGGNTAT GGQ---EELK ADIVMYTENA YLETPDTHVV YKPGTSDD--

D_19_ABA00002 -KDFDIDLAF FDTPGGNPPA GGSGT-EEYK ADIVMYTENV NLETPDTHVV YKPGKEDE--

D_64_ABN10535 -KDFDIDLAF FDTPGGNPPA GGSGT-EEYK ADIVMYTENV NLETPDTHVV YKPAKEDE--

D_27_ABA00009 -KELDIDFAF FDTDSGD--- ------TEYK ADIVMYAENV NLETPDTHVV YKPGKEDD--

D_28_ACQ91158 -KELDIDLAF FDTDGGTAD- ------TEYK ADIVMYAENV NLETPDTHVV YKPGKEDD--

D_20_ABA00003 -KNHDITMAF FDTPGGQLNG K-----DELK ADIVMYTENV NLETPDTHVV YKPGKEDD--

D_60_AEK87026 -KNHDITMAF FDTPGGQLNG K-----DELK ADIVMYTENV NLETPDTHVV YKPGTSDD--

D_71_AGT76762 -KNHDITMAF FDTPGGQLNG K-----DELK ADIVMYTENV NLETPDTHVV YKPGTSDD--

D_42_ABA00019 -ENHDITMAF FDTPGGTITG GTGGPQDELK ADIVMYTENI NLETPDTHVV YKPGKEDD--

D_51_ABA00026 -KEFDIDLAF FDTPGGKLPG NDQK--EEYK ADIVMYTENA YLETPDTHVV YKPGKDDA--

D_30_ABA00012 -EDFDIDLAF FDTPGGTLTG GG----TEYK ADIVMCTENV NLETPDTHVV YKPGKDDD--

D_29_BAJ22326 -EDFDIDLAF FDTPGGTLTG GG----TEYK ADIVMCTENV NLETPDTHVV YKPGKDDD--

D_56_ADM66117 -EDFDIDLAF FDTPGGTLTG GG----TEYK ADIVMCTENV NLETPDTHVV YKPGKDDD--

D_69_AFK92217 -EDFDIDLAF FDTPGGTLTG GG----TEYK ADIVMCTENV NLETPDTHVV YKPGKDDD--

D_15_BAJ22290 -EDFDIDLAF FDTPGGTLTG GG----TEYK ADIVMCTENV NLETPDTHVV YKPGKDDD--

B_35_AAP92351 -IEYDIDMEF FDNSSQR--- ------TNFS PKIVMYAENV GLETPDTHVV YKPGTEDT--

B_11_ACZ06785 -VEYDIDMEF FDAASQK--- ------TNLS PKIVMYAENV NLETPDTHVV YKPESEDT--

B_55_AIS92536 -VEYDIDMEF FDAASQK--- ------TNLS PKIVMYAENV NLETPDTHVV YKPESEDT--

B_21_AAG21823 -VEYDIDMNF FDEASQK--- ------ANFS PKIVMYAENV DLETPDTHVV YKPGTSEE--

B_50_ABA00027 -VEYDIDMNF FDLRSQM--- ------TGLK PKIVMYAENV DLETPDTHVV YKPGASDA--

B_14_AAZ99996 -IEYDIDMNF FDLRSQK--- ------QGLK PKIVMYAENV DLESPDTHVV YKPEVSDA--

B_34_AAW33485 -IEYDIDMNF FDLRSQR--- ------SELK PKIVMYAENV DLECPDTHVV YKPGVSDA--

B_79_BAW32492 -IEYDIDMNF FDLRSQR--- ------SELK PKIVMYAENV DLESPDTHVV YKPGVSDA--

B_68_AET87230 -EEPDIDMEF FDGRDAVA-- ------GALA PEIVLYTENV NLETPDSHVV YKPGTSD---

B_66_AET87148 AEEPDIDMEF FDGREAA--- ------DAFS PEIVLYTENV NLETPDSHVV YKPGTSDG--

E_4_AAD03660 -PNYDIDLAF FDGKNIV--- ------ANYD PDIVMYTENV DLQTPDTHIV YKPGKEDT--

B_16_AAW33444 -PNYDIDLAF FDNKNIA--- ------ANYD PDIVMYTENV DLQTPDTHIV YKPGTEDT--

A_61_AEK79922 --DNNVELRF FDTAN----- ------NAAT PQVVFYTEDV NLEMPDTHLV FKPAVPNGTI

A_12_BAG48789 --DDNIELKF FDSANN---- ------AANT AQVVFYTEDV NLEMPDTHLV FKPTVTNGTI

A_18_AAZ99994 -NNEGVEMQF FASAAD---- ------NQND PSVVLYSEDV NLEAPDTHIV FKPAVAADTV

A_31_CAO78638 -NNDGVEMRF FASSAND--- ------NQND PTVVMYSEDV NLEAPDTHIV YKPAVTADTI

G_52_ABK35044 -ADTQVDMQF FATTA----- ------ANTT PKAVLYAEDV NLEGPDTHLV FKPTVTEGTT

F_41_ACH90432 TLTSDVNLQF FALPS----- ------TPNE PKAVLYAENV SIEAPDTHLV YKPDVAQGTI

F_40_AMQ95234 -TTTNVGLNF FTTATET--- ------ANFS PKVVLYSEDV NLEAPDTHLV FKPDVNGT--

....|....| ....|....| ....|....| ....|....| ....|....| ....|....|

365 375 385 395 405 415

HexN1 SSEINLVQQS MPNRPNYIGF RDNFVGLMYY NSTGNMGVLA GQASQLNAVV DLQDRNTELS

B_3_AAW33168 NSHANLGQQA MPNRPNYIGF RDNFVGLMYY NSTGNMGVLA GQASQLNAVV DLQDRNTELS

C_5_AAW65514 NSRELMGQQS MPNRPNYIAF RDNFIGLMYY NSTGNMGVLA GQASQLNAVV DLQDRNTELS

B_7_AAD03663 NSHANLGQQA MPNRPNYIGF RDNFVGLMYY NSTGNMGVLA GQASQLNAVV DLQDRNTELS

C_2_AAO24095 NSKAMLGQQS MPNRPNYIAF RDNFIGLMYY NSTGNMGVLA GQASQLNAVV DLQDRNTELS

C_1_AAQ10553 NSKAMLGQQA MPNRPNYIAF RDNFIGLMYY NSTGNMGVLA GQASQLNAVV DLQDRNTELS

C_57_ADM46153 NSKAMLGQQS MPNRPNYIAF RDNFIGLMYY NSTGNMGVLA GQASQLNAVV DLQDRNTELS

C_6_ADV03661 NAKVMLGQQA MPNRPNYIAF RDNFIGLMYY NSTGNMGVLA GQASQLNAVV DLQDRNTELS

D_37_ABA00016 SSEANLTQQS MPNRPNYIGF RDNFVGLLYY NSTGNMGVLA GQASQLNAVV DLQDRNTELS

D_13_ABA00000 SSEVNLTQQS MPNRPNYIGF RDNFVGLMYY NSTGNMGVLA GQASQLNAVV DLQDRNTELS

D_70_AKI33526 SSEANLTQQS MPNRPNYIGF RDNFVGLLYY NSTGNMGVLA GQASQLNAVV DLQDRNTELS

D_63_AEV92966 SSEANLTQQS MPNRPNYIGF RDNFVGLMYY NSTGNMGVLA GQASQLNAVV DLQDRNTELS

D_72_AGT78001 SSEANLTQQS MPNRPNYIGF RDNFVGLMYY NSTGNMGVLA GQASQLNAVV DLQDRNTELS

D_23_ABA00005 SSEINLGQQS MPNRPNYIGF RDNFVGLMYY NSTGNMGVLA GQASQLNAVV DLQDRNTELS

D_24_ABA00006 SSSANLTQQA MPNRPNYIGF RDNFVGLMYY NSTGNMGVLA GQASQLNAVV DLQDRNTELS

D_39_ABA00018 SSEINLTQQS MANRPNYIGF RDNFVGLMYY NSTGNMGVLA GQASQLNAVV DLQDRNTELS

D_43_ABA00020 SSEINLTQQS MANRPNYIGF RDNFVGLMYY NSTGNMGVLA GQASQLNAVV DLQDRNTELS

D_58_ADW95419 SSEINLTQQS MANRPNYIGF RDNFVGLMYY NSTGNMGVLA GQASQLNAVV DLQDRNTELS

D_33_ABA00014 SSEINLTQQS MANRPNYIGF RDNFVGLMYY NSTGNMGVLA GQASQLNAVV DLQDRNTELS

D_38_ABA00017 SSEVNLTQQS MPNRPNYIGF RDNFVGLMYY NSTGNMGVLA GQASQLNAVV DLQDRNTELS

D_36_ACY04472 SSAANLTQQS MPNRPNYIGF RDNFVGLMYY NSTGNMGVLA GQASQLNAVV DLQDRNTELS

D_49_ABA00025 SSEINLCQQS MPNRPNYIGF RDNFVGLMYY NSTGNMGVLA GQASQLNAVV DLQDRNTELS

D_8_AAZ99998 SSAANLTQQS MPNRPNYIGF RDNFVGLMYY NSTGNMGVLA GQASQLNAVV DLQDRNTELS

D_46_ABA00023 SSEANLLQQS MPNRPNYIGF RDNFVGLMYY NSTGNMGVLA GQASQLNAVV DLQDRNTELS

D_47_ABA00024 SSEVNLTQQS MPNRPNYIGF RDNFVGLMYY NSTGNMGVLA GQASQLNAVV DLQDRNTELS

D_44_ABA00021 SSEANLVQQS MPNRPNYIGF RDNFVGLMYY NSTGNMGVLA GQASQLNAVV DLQDRNTELS

D_48_ABO61301 SSESNLTQQS MPNRPNYIGF RDNFVGLMYY NSTGNMGVLA GQASQLNAVV DLQDRNTELS

D_22_ABA00004 SSEANLAQQS MPNRPNYIGF RDNFVGLMYY NSTGNMGVLA GQASQLNAVV DLQDRNTELS

D_53_BAJ46350 SSEANLAQQS MPNRPNYIGF RDNFVGLMYY NSTGNMGVLA GQASQLNAVV DLQDRNTELS

D_45_ABA00022 NSEINLTQQS MPNRPNYIGF RDNFVGLMYY NSTGNMGVLA GQASQLNAVV DLQDRNTELS

D_26_ABA00008 SSEINLVQQS MPNRPNYIGF RDNFVGLMYY NSTGNMGVLA GQASQLNAVV DLQDRNTELS

D_32_ABA00013 SSEANLVQQS MPNRPNYIGF RDNFVGLMYY NSTGNMGVLA GQASQLNAVV DLQDRNTELS

D_73_APD28385 SSEINLTQQS MPNRPNYIGF RDNFVGLMYY NSTGNMGVLA GQASQLNAVV DLQDRNTELS

D_67_BAL63187 SSEANLVQQS MPNRPNYIGF RDNFVGLMYY NSTGNMGVLA GQASQLNAVV DLQDRNTELS

D_9_BAE66671 SSEINLTQQS MPNRPNYIGF RDNFVGLMYY NSTGNMGVLA GQASQLNAVV DLQDRNTELS

D_54_YP_0030386 SSEINLTQQS MPNRPNYIGF RDNFVGLMYY NSTGNMGVLA GQASQLNAVV DLQDRNTELS

D_17_ADY18429 SSEVNLTQQS MPNRPNYIGF RDNFVGLMYY NSTGNMGVLA GQASQLNAVV DLQDRNTELS

D_10_AAZ99999 SSETNLTQQS MPNRPNYIGF RDNFVGLMYY NSTGNMGVLA GQASQLNAVV DLQDRNTELS

D_65_BAL41721 SSEINLTQQS MPNRPNYIGF RDNFVGLMYY NSTGNMGVLA GQASQLNAVV DLQDRNTELS

D_62_AEL78842 SSEINLVQQS MPNRPNYIGF RDNFVGLMYY NSTGNMGVLA GQASQLNAVV DLQDRNTELS

D_25_ABA00007 SSAANLVQQS MPNRPNYIGF RDNFVGLMYY NSTGNMGVLA GQASQLNAVV DLQDRNTELS

D_59_AEI91289 SSAANLVQQS MPNRPNYIGF RDNFVGLMYY NSTGNMGVLA GQASQLNAVV DLQDRNTELS

D_19_ABA00002 SSEANLVQQS MPNRPNYIGF RDNFVGLMYY NSTGNMGVLA GQASQLNAVV DLQDRNTELS

D_64_ABN10535 SSQANLVQQS MPNRPNYIGF RDNFVGLMYY NSTGNMGVLA GQASQLNAVV DLQDRNTELS

D_27_ABA00009 SSEANLVQQS MPNRPNYIGF RDNFVGLMYY NSTGNMGVLA GQASQLNAVV DLQDRNTELS

D_28_ACQ91158 SSEINLVQQS MPNRPNYIGF RDNFVGLMYY NSTGNMGVLA GQASQLNAVV DLQDRNTELS

D_20_ABA00003 SSEINLVQQS MPNRPNYIGF RDNFVGLMYY NSTGNMGVLA GQASQLNAVV DLQDRNTELS

D_60_AEK87026 SSEINLVQQS MPNRPNYIGF RDNFVGLMYY NSTGNMGVLA GQASQLNAVV DLQDRNTELS

D_71_AGT76762 SSEINLVQQS MPNRPNYIGF RDNFVGLMYY NSTGNMGVLA GQASQLNAVV DLQDRNTELS

D_42_ABA00019 SSEINLVQQS MPNRPNYIGF RDNFVGLMYY NSTGNMGVLA GQASQLNAVV DLQDRNTELS

D_51_ABA00026 SSEANLVQQS MPNRPNYIGF RDNFVGLMYY NSTGNMGVLA GQASQLNAVV DLQDRNTELS

D_30_ABA00012 SSEINLVQQS MPNRPNYIGF RDNFVGLMYY NSTGNMGVLA GQASQLNAVV DLQDRNTELS

D_29_BAJ22326 SSEINLVQQS MPNRPNYIGF RDNFVGLMYY NSTGNMGVLA GQASQLNAVV DLQDRNTELS

D_56_ADM66117 SSEINLVQQS MPNRPNYIGF RDNFVGLMYY NSTGNMGVLA GQASQLNAVV DLQDRNTELS

D_69_AFK92217 SSEINLVQQS MPNRPNYIGF RDNFVGLMYY NSTGNMGVLA GQASQLNAVV DLQDRNTELS

D_15_BAJ22290 SSEINLVQQS MPNRPNYIGF RDNFVGLMYY NSTGNMGVLA GQASQLNAVV DLQDRNTELS

B_35_AAP92351 SSEANLGQQS MPNRPNYIGF RDNFIGLMYY NSTGNMGVLA GQASQLNAVV DLQDRNTELS

B_11_ACZ06785 SSEANLGQQS MPNRPNYIGF RDNFIGLMYY NSTGNMGVLA GQASQLNAVV DLQDRNTELS

B_55_AIS92536 SSEANLGQQS MPNRPNYIGF RDNFIGLMYY NSTGNMGVLA GQASQLNAVV DLQDRNTELS

B_21_AAG21823 SSHANLGQQS MPNRPNYIGF RDNFIGLMYY NSTGNMGVLA GQASQLNAVV DLQDRNTELS

B_50_ABA00027 SSHANLGQQS MPNRPNYIGF RDNFIGLMYY NSTGNMGVLA GQASQLNAVV DLQDRNTELS

B_14_AAZ99996 SSNANLGQQS MPNRPNYIGF RDNFIGLMYY NSTGNMGVLA GQASQLNAVV DLQDRNTELS

B_34_AAW33485 SSETNLGQQS MPNRPNYIGF RDNFIGLMYY NSTGNMGVLA GQASQLNAVV DLQDRNTELS

B_79_BAW32492 SSETNLGQQS MPNRPNYIGF RDNFIGLMYY NSTGNMGVLA GQASQLNAVV DLQDRNTELS

B_68_AET87230 NSHANLGQQA MPNRPNYIGF RDNFVGLMYY NSTGNMGVLA GQASQLNAVV DLQDRNTELS

B_66_AET87148 NSHANLGQQA MPNRPNYIGF RDNFVGLMYY NSTGNMGVLA GQASQLNAVV DLQDRNTELS

E_4_AAD03660 SSESNLGQQA MPNRPNYIGF RDNFIGLMYY NSTGNMGVLA GQASQLNAVV DLQDRNTELS

B_16_AAW33444 SSESNLGQQA MPNRPNYIGF RDNFIGLMYY NSTGNMGVLA GQASQLNAVV DLQDRNTELS

A_61_AEK79922 ASESLLGQQA APNRANYIAF RDNFIGLMYY NSTGNMGVLA GQASQLNAVV DLQDRNTELS

A_12_BAG48789 ASESLLGQQA APNRANYIAF RDNFIGLMYY NSTGNMGVLA GQASQLNAVV DLQDRNTELS

A_18_AAZ99994 SSELLLGQQA APNRPNYIGF RDNFIGLMYY NSTGNMGVLA GQASQLNAVV DLQDRNTELS

A_31_CAO78638 SSELLLGQQA APNRPNYIAF RDNFIGLMYY NSTGNMGVLA GQASQLNAVV DLQDRNTELS

G_52_ABK35044 SAEALLAQQA APNRPNYIAF RDNFIGLMYY NSTGNMGVLA GQASQLNAVV DLQDRNTELS

F_41_ACH90432 SSADLLTQQA APNRPNYIGF RDNFIGLMYY NSTGNMGVLA GQASQLNAVV DLQDRNTELS

F_40_AMQ95234 SAELLLGQQA APNRPNYIGF RDNFIGLMYY NSTGNMGVLA GQASQLNAVV DLQDRNTELS

....|....| ....|....| ....|....| ....|....| ....|....| ....|....|

425 435 445 455 465 475

HexN1 YQLLLDSLGD RTRYFSMWNS AVDSYDPDVR IIENHGVEDE LPNYCFPLDG SGTNAA-YQG

B_3_AAW33168 YQLLLDSLGD RTRYFSMWNQ AVDSYDPDVR IIENHGIEDE LPNYCFPLNG IGPGHT-YQG

C_5_AAW65514 YQLLLDSIGD RTRYFSMWNQ AVDSYDPDVR IIENHGTEDE LPNYCFPLGG VINTET-LTK

B_7_AAD03663 YQLLLDSLGD RTRYFSMWNQ AVDSYDPDVR IIENHGIEDE LPNYCFPLDG IGPAKT-YQG

C_2_AAO24095 YQLLLDSIGD RTRYFSMWNQ AVDSYDPDVR IIENHGTEDE LPNYCFPLGG IGVTDT-YQA

C_1_AAQ10553 YQLLLDSIGD RTRYFSMWNQ AVDSYDPDVR IIENHGTEDE LPNYCFPLGG IGVTDT-YQG

C_57_ADM46153 YQLLLDSIGD RTRYFSMWNQ AVDSYDPDVR IIENHGTEDE LPNYCFPLGG IGVTDT-YQA

C_6_ADV03661 YQLLLDSIGD RTRYFSMWNQ AVDSYDPDVR IIENHGTEDE LPNYCFPLGG IGITDT-FQA

D_37_ABA00016 YQLLLDSLGD RTRYFSMWNS AVDSYDPDVR IIENHGVEDE LPNYCFPLDG VQTNSA-YQG

D_13_ABA00000 YQLLLDSLGD RTRYFSMWNS AVDSYDPDVR IIENHGVEDE LPNYCFPLDG IQTNSA-YQG

D_70_AKI33526 YQLLLDSLGD RTRYFSMWNS AVDSYDPDVR IIENHGVEDE LPNYCFPLDG VQTNSA-YQG

D_63_AEV92966 YQLLLDSLGD RTRYFSMWNS AVDSYDPDVR IIENHGVEDE LPNYCFPLDG VQTNSA-YQG

D_72_AGT78001 YQLLLDSLGD RTRYFSMWNS AVDSYDPDVR IIENHGVEDE LPNYCFPLDG VQTNSA-YQG

D_23_ABA00005 YQLLLDSLGD RTRYFSMWNS AVDSYDPDVR IIENHGVEDE LPNYCFPLDG VATNTV-YQG

D_24_ABA00006 YQLLLDSLGD RTRYFSMWNS AVDSYDPDVR IIENHGVEDE LPNYCFPLNG SGSNST-YKG

D_39_ABA00018 YQLLLDSLGD RTRYFSMWNS AVDSYDPDVR IIENHGVEDE LPNYCFPLDG MGSNAA-YQG

D_43_ABA00020 YQLLLDSLGD RTRYFSMWNS AVDSYDPDVR IIENHGVEDE LPNYCFPLDG MGSNAA-YQG

D_58_ADW95419 YQLLLDSLGD RTRYFSMWNS AVDSYDPDVR IIENHGVEDE LPNYCFPLDG CGSSTA-FQG

D_33_ABA00014 YQLLLDSLGD RTRYFSMWNS AVDSYDPDVR IIENHGVEDE LPNYCFPLDG SGSSTA-FQG

D_38_ABA00017 YQLLLDSLGD RTRYFSMWNS AVDSYDPDVR IIENHGVEDE LPNYCFPLDG SGSSST-YQG

D_36_ACY04472 YQLLLDSLGD RTRYFSMWNS AVDSYDPDVR IIENHGVEDE LPNYCFPLDG SGSNTA-YQG

D_49_ABA00025 YQLLLDSLGD RTRYFSMWNS AVDSYDPDVR IIENHGVEDE LPNYCFPLDG SGSSTA-YQG

D_8_AAZ99998 YQLLLDSLGD RTRYFSMWNS AVDSYDPDVR IIENHGVEDE LPNYCFPLDG TGTNAT-YQG

D_46_ABA00023 YQLLLDSLGD RTRYFSMWNS AVDSYDPDVR IIENHGVEDE LPNYCFPLNG MGSNAA-YQG

D_47_ABA00024 YQLLLDSLGD RTRYFSMWNS AVDSYDPDVR IIENHGVEDE LPNYCFPLDG TGTASA-YQG

D_44_ABA00021 YQLLLDSLGD RTRYFSMWNS AVDSYDPDVR IIENHGVEDE LPNYCFPLDG AGTNAV-YQG

D_48_ABO61301 YQLLLDSLGD RTRYFSMWNS AVDSYDPDVR IIENHGVEDE LPNYCFPLDG AGTNAV-YQG

D_22_ABA00004 YQLLLDSLGD RTRYFSMWNS AVDSYDPDVR IIENHGVEDE LPNYCFPLDG TGTNST-YQG

D_53_BAJ46350 YQLLLDSLGD RTRYFSMWNS AVDSYDPDVR IIENHGVEDE LPNYCFPLDG TGTNST-YQG

D_45_ABA00022 YQLLLDSLGD RTRYFSMWNS AVDSYDPDVR IIENHGVEDE LPNYCFPLNG TGTNST-YQG

D_26_ABA00008 YQLLLDSLGD RTRYFSMWNS AVDSYDPDVR IIENHGVEDE LPNYCFPLNG TGTNST-YQG

D_32_ABA00013 YQLLLDSLGD RTRYFSMWNS AVDSYDPDVR IIENHGVEDE LPNYCFPLDG AGTNAT-YQG

D_73_APD28385 YQLLLDSLGD RTRYFSMWNS AVDSYDPDVR IIENHGVEDE LPNYCFPLDG AGTNAT-YQG

D_67_BAL63187 YQLLLDSLGD RTRYFSMWNS AVDSYDPDVR IIENHGVEDE LPNYCFPLDG AGTNAT-YQG

D_9_BAE66671 YQLLLDSLGD RTRYFSMWNS AVDSYDPDVR IIENHGVEDE LPNYCFPLDG AGTNAT-YQG

D_54_YP_0030386 YQLLLDSLGD RTRYFSMWNS AVDSYDPDVR IIENHGVEDE LPNYCFPLDG AGTNAT-YQG

D_17_ADY18429 YQLLLDSLGD RTRYFSMWNS AVDSYDPDVR IIENHGVEDE LPNYCFPLNG TGTNST-YLG

D_10_AAZ99999 YQLLLDSLGD RTRYFSMWNS AVDSYDPDVR IIENHGVEDE LPNYCFPLNG TGTNST-YQG

D_65_BAL41721 YQLLLDSLGD RTRYFSMWNS AVDSYDPDVR IIENHGVEDE LPNYCFPLNG TGTNST-YQG

D_62_AEL78842 YQLLLDSLGD RTRYFSMWNS AVDSYDPDVR IIENHGVEDE LPNYCFPLDG SGTNST-YQG

D_25_ABA00007 YQLLLDSLGD RTRYFSMWNS AVDSYDPDVR IIENHGVEDE LPNYCFPLDG SGTNSA-YQG

D_59_AEI91289 YQLLLDSLGD RTRYFSMWNS AVDSYDPDVR IIENHGVEDE LPNYCFPLDG SGTNAA-YQG

D_19_ABA00002 YQLLLDSLGD RTRYFSMWNS AVDSYDPDVR IIENHGVEDE LPNYCFPLDG SGTNAA-YQG

D_64_ABN10535 YQLLLDSLGD RTRYFSMWNS AVDSYDPDVR IIENHGVEDE LPNYCFPLDG SGTNAA-YQG

D_27_ABA00009 YQLLLDSLGD RTRYFSMWNS AVDSYDPDVR IIENHGVEDE LPNYCFPLDG SGTNST-YQG

D_28_ACQ91158 YQLLLDSLGD RTRYFSMWNS AVDSYDPDVR IIENHGVEDE LPNYCFPLDG LGTNAT-YQG

D_20_ABA00003 YQLLLDSLGD RTRYFSMWNS AVDSYDPDVR IIENHGVEDE LPNYCFPLDG AGTNAV-YQG

D_60_AEK87026 YQLLLDSLGD RTRYFSMWNS AVDSYDPDVR IIENHGVEDE LPNYCFPLDG AGTNAV-YQG

D_71_AGT76762 YQLLLDSLGD RTRYFSMWNS AVDSYDPDVR IIENHGVEDE LPNYCFPLDG AGTNAV-YQG

D_42_ABA00019 YQLLLDSLGD RTRYFSMWNS AVDSYDPDVR IIENHGVEDE LPNYCFPLDG SGTNSA-FQG

D_51_ABA00026 YQLLLDSLGD RTRYFSMWNS AVDSYDPDVR IIENHGVEDE LPNYCFPLDG SGTNAA-YQG

D_30_ABA00012 YQLLLDSLGD RTRYFSMWNS AVDSYDPDVR IIENHGVEDE LPNYCFPLDG SGTNAA-YEG

D_29_BAJ22326 YQLLLDSLGD RTRYFSMWNS AVDSYDPDVR IIENHGVEDE LPNYCFPLDG SGTNAA-YEG

D_56_ADM66117 YQLLLDSLGD RTRYFSMWNS AVDSYDPDVR IIENHGVEDE LPNYCFPLDG SGTNAA-YEG

D_69_AFK92217 YQLLLDSLGD RTRYFSMWNS AVDSYDPDVR IIENHGVEDE LPNYCFPLDG SGTNAA-YEG

D_15_BAJ22290 YQLLLDSLGD RTRYFSMWNS AVDSYDPDVR IIENHGVEDE LPNYCFPLDG SGTNAA-YEG

B_35_AAP92351 YQLLLDSLGD RTRYFSMWNQ AVDSYDPDVR VIENHGVEDE LPNYCFPLDG IGVPTTSYKS

B_11_ACZ06785 YQLLLDSLGD RTRYFSMWNQ AVDSYDPDVR VIENHGVEDE LPNYCFPLNG IGVPTTSYKS

B_55_AIS92536 YQLLLDSLGD RTRYFSMWNQ AVDSYDPDVR VIENHGVEDE LPNYCFPLNG IGVPTTSYKS

B_21_AAG21823 YQLLLDSLGD RTRYFSMWNQ AVDSYDPDVR IIENHGVEDE LPNYCFPLDG VGVPISSYKI

B_50_ABA00027 YQLLLDSLGD RTRYFSMWNQ AVDSYDPDVR VIENHGVEDE LPNYCFPLDG VGPRIDSYKG

B_14_AAZ99996 YQLLLDSLGD RTRYFSMWNQ AVDSYDPDVR VIENHGVEDE LPNYCFPLDG IGPRTDSYKE

B_34_AAW33485 YQLLLDSLGD RTRYFSMWNQ AVDSYDPDVR VIENHGVEDE LPNYCFPLDG VGPRTDSYKE

B_79_BAW32492 YQLLLDSLGD RTRYFSMWNQ AVDSYDPDVR VIENHGVEDE LPNYCFPLDG VGPQTDSYKE

B_68_AET87230 YQLLLDSLGD RTRYFSMWNQ AVDSYDPDVR IIENHGIEDE LPNYCFPLDG IGPGHT-YQG

B_66_AET87148 YQLLLDSLGD RSRYFSMWNQ AVDSYDPDVR IIENHGVEDE LPNYCFPLDG IGPGNK-YQG

E_4_AAD03660 YQLLLDSLGD RTRYFSMWNQ AVDSYDPDVR IIENHGVEDE LPNYCFPLNG VGLTDT-YQG

B_16_AAW33444 YQLLLDSLGD RTRYFSMWNQ AVDSYDPDVR IIENHGVEDE LPNYCFPLNG VGFTDT-YQG

A_61_AEK79922 YQLMLDALGD RARYFSLWNS AVDSYDPDVR IIENHGVEDE LPNYCFPLSA VGDIKS-YKG

A_12_BAG48789 YQLMLDALGD RTRYFSLWNS AVDSYDPDVR VIENHGVEDE LPNYCFPLSA VGEIKN-YKG

A_18_AAZ99994 YQLMLDALGD RSRYFSMWNS AVDSYDPDVR IIENHGVEDE LPNYCFPLGA VGIVNS-YKG

A_31_CAO78638 YQLMLDALGD RSRYFSMWNS AVDSYDPDVR IIENHGVEDE LPNYCFPLGA VGVVNS-YKG

G_52_ABK35044 YQLMLDALGD RSRYFSMWNQ AVDSYDPDVR IVENHGVEDE LPNYCFPLGG MAVTDT-YSP

F_41_ACH90432 YQLMLDALGD RSRYFSMWNQ AVDSYDPDVR IIENHGVEDE LPNYCFPLGG SAATDT-YSG

F_40_AMQ95234 YQLMLDALGD RSRYFSMWNQ AVDSYDPDVR IIENHGVEDE LPNYCFPLNG QGISNS-YQG

....|....| ....|....| ....|....| ....|....| ....|....| ....|....|

485 495 505 515 525 535

HexN1 VKVKTGQNDE -----WEKDT -NVAARN-QI CKGNIYAMEI NLQANLWKSF LYSNVALYLP

B_3_AAW33168 IKVKTDDTNG -----WEKDA -NVAPAN-EI TIGNNLAMEI NIQANLWRSF LYSNVALYLP

C_5_AAW65514 VKPKTGQENG -----WEKDA TEFSDKN-EI RVGNNFAMEI NLNANLWRNF LYSNIALYLP

B_7_AAD03663 IKSKDNG--- -----WEKDD -NVSKSN-EI AIGNNQAMEI NIQANLWRSF LYSNVALYLP

C_2_AAO24095 IKANGNGSGD NGDITWTKDE -TFATRN-EI GVGNNFAMEI NLNANLWRNF LYSNIALYLP

C_1_AAQ10553 IKSNGNGNPQ N----WTKND -DFAARN-EI GVGNNFALEI NLNANLWRNF LYSNIALYLP

C_57_ADM46153 IKATNGNGGA TT---WAQDN -TFAERN-EI GVGNNFAMEI NLNANLWRNF LYSNIALYLP

C_6_ADV03661 VKTTAANGDQ GNTT-WQKDS -TFAERN-EI GVGNNFAMEI NLNANLWRNF LYSNIALYLP

D_37_ABA00016 VKLKPDQTGG GVNGDWVKDD -DISAHN-QI GKGNIFAMEI NLQANLWKSF LYSNVALYLP

D_13_ABA00000 VKLKDNPTGG GANGDWVKDD -TISAHN-QI GKGNIFAMEI NLQANLWKSF LYSNVALYLP

D_70_AKI33526 VKLKANPAGG GANGDWEKDD -TISVHN-QI GKGNIFAMEI NLQANLWKSF LYSNVALYLP

D_63_AEV92966 VKLKANQAGG GANGDWEKDD -TISAHN-QI GKGNIFAMEI NLQANLWKSF LYSNVALYLP

D_72_AGT78001 VKLKANQAGG GANGDWEKDD -TISAHN-QI GKGNIFAMEI NLQANLWKSF LYSNVALYLP

D_23_ABA00005 VKLQTGQTDK -----WQKDD -DVSTQN-RI GKGSVFAMEI NLQANLWKSF LYSNVALYLP

D_24_ABA00006 VKAGTGNN-- -----WDDDE -NVARQN-QI GTGNLFAMEI NLQANLWKSF LYSNVALYLP

D_39_ABA00018 VKPKTGNG-- -----WDPDT -DVAAQN-QI AKGNIYAMEI NLQANLWKSF LYSNVALYLP

D_43_ABA00020 VKPKTGNG-- -----WDPNT -DVAAQN-QI AKGNIYAMEI NLQANLWKSF LYSNVALYLP

D_58_ADW95419 VKVTNPATST NNNTQWGVND -EVATHN-QI ARGNLYAMEI NLQANLWKSF LYSNVALYLP

D_33_ABA00014 VKVKTPAGTG QNTV-WEVNN -DAATHN-QI ARGNLYAMEI NLQANLWKSF LYSNVALYLP

D_38_ABA00017 VKYENGAGGN GT---WKVDD -TVTRQN-QI AKGNLYAMEI NLQANLWKSF LYSNVALYLP

D_36_ACY04472 VKYENGAGNG S----WKVDG -EVASQN-QI AKGNLYAMEI NLQANLWKSF LYSNVALYLP

D_49_ABA00025 VEPDTTVAGT NDK--WKVNA -KVAQHN-QI AKGNLFAMEI NLQANLWKSF LYSNVALYLP

D_8_AAZ99998 VEPDNAQGQN DK---WKKDE -KVAAQN-QI CKGNIYAMEI NLQANLWKSF LYSNVALYLP

D_46_ABA00023 VKPKDNGG-- -----WDPNT -NAARQN-RI AMGNVYAMEI NLQANLWKSF LYSNVALYLP

D_47_ABA00024 VKVKNGTVAG KVE--WDPDT -KVASKN-RI CKGSIFAMEI NLQANLWKSF LYSNVALYLP

D_44_ABA00021 VKEQLNQNDK -----WEVDN -AISNQN-RI CKGNVYAMEI NLQANLWKSF LYSNVALYLP

D_48_ABO61301 VKVKTTNNTE -----WEKDT -AVSEHN-QI CKGNVYAMEI NLQANLWKSF LYSNVALYLP

D_22_ABA00004 VKETAAQNG- -----WEKDP -NVAAQN-QI CKGNIYAMEI NLQANLWKSF LYSNVALYLP

D_53_BAJ46350 VKETAAQNG- -----WEKDP -NVAAQN-QI CKGNIYAMEI NLQANLWKSF LYSNVALYLP

D_45_ABA00022 VKITGNNDGD LETE-WERDE -AISRQN-QI CKGNVYAMEI NLQANLWKSF LYSNVALYLP

D_26_ABA00008 VKITNGNDGA EESE-WEKDD -AISRQN-QI CKGNVYAMEI NLQANLWKSF LYSNVALYLP

D_32_ABA00013 VKVKDGEDGN ENAD-WVKDP -NLASRN-QI CKGNIFAMEI NLQANLWKSF LYSNVALYLP

D_73_APD28385 VKVKNGEDGD VNAD-WEKDP -NLASRN-QI CKGNIFAMEI NLHANLWKSF LYSNVALYLP

D_67_BAL63187 VKVKNGQDGD VNAD-WEKDP -NLASRN-QI CKGNIFAMEI NLQANLWKSF LYSNVALYLP

D_9_BAE66671 VKVKNGQDGD NNAE-WEKDN -AVADRN-QI CKGNIVAMEI TLQANLWKSF LYSNVALDLP

D_54_YP_0030386 VKVKNGQDGD NNAE-WEKDN -AVADRN-QI CKGNIVAMEI NLQANLWKSF LYSNVALYLP

D_17_ADY18429 VKVKPDQDGD VESE-WDKDD -TIARQN-QI AKGNVFAMEI NLQANLWKSF LYSNVALYLP

D_10_AAZ99999 VKVKTGQDGA EETE-WDKDE -TVARQN-QI AKGNVYAMEI NLQANLWKSF LYSNVALYLP

D_65_BAL41721 VKVKTGQDGA EETE-WEKDE -TVARQN-QI AKGNVYAMEI NLQANLWKSF LYSNVALYLP

D_62_AEL78842 VKVTTNEGAL ESE--WGKDE -SVARQN-QI CKGNIYAMEI NLQANLWKSF LYSNVALYLP

D_25_ABA00007 VKVKNGEDGD IESE-WEKDT -NVAARN-QL CKGNIFAMEI NLQANLWKSF LYSNVALYLP

D_59_AEI91289 VKVKNGEDGD IESE-WEKDT -NVAARN-QL CKGNIFAMEI NLQANLWKSF LYSNVALYLP

D_19_ABA00002 VKVQDGEDGD KETE-WEKDT -KVADRN-QL CKGNIFAMEI NLQANLWKSF LYSNVALYLP

D_64_ABN10535 VKVQDGEDGD KETE-WEKDT -KVADRN-QL CKGNIFAMEI NLQANLWKSF LYSNVALYLP

D_27_ABA00009 VKITDGNGAL QNG--WAKDD -AISRQN-QI CKGNIYAMEI NLQANLWKSF LYSNVALYLP

D_28_ACQ91158 VKVSTGDGAT QSG--WAKDD -TMARQN-QI CRGNIYAMEI NLQANLWKSF LYSNVALYLP

D_20_ABA00003 VKITDGNDGD VNDD-WEKDT -AVSERN-QI CKGNIYAMEI NLQANLWKSF LYSNVGLYLP

D_60_AEK87026 VKITDGNDGD VNDD-WEKDT -AVSERN-QI CKGNIYAMEI NLQANLWKSF LYSNVALYLP

D_71_AGT76762 VKITDGNDGD VNDD-WEKDT -AVSERN-QI CKGNIYAMEI NLQANLWKSF LYSNVALYLP

D_42_ABA00019 VKIKQNQDGD VNDD-WEKDD -KVSTQN-QI CKGNIYAMEI NLQANLWKSF LYSNVALYLP

D_51_ABA00026 VKVTEGEDGA EESE-WELEN -KLAARN-QI CKGNIFAMEI NLQANLWRSF LYSNVALYLP

D_30_ABA00012 VKVKTGQDGD QETE-WEKDT -NVADRN-QI CKGNIYAMEI NLQANLWKSF LYSNVALYLP

D_29_BAJ22326 VKVKNGQDGD QESE-WEKDT -NVADRN-QI CKGNIYAMEI NLQANLWKSF LYSNVALYLP

D_56_ADM66117 VKVKNGQDGD QESE-WEKDT -NVADRN-QI CKGNIYAMEI NLQANLWKSF LYSNVALYLP

D_69_AFK92217 VKVKNGEDGD QESE-WEKDT -NVADRN-QI CKGNIYAMEI NLQANLWKSF LYSNVALYLP

D_15_BAJ22290 VKVKNGEDGD QESE-WEKDT -NVADRN-QI CKGNIYAMEI NLQANLWKSF LYSNVALYLP

B_35_AAP92351 IVPNGEDNNN -----W-KEP -EVNGTS-EI GQGNLFAMEI NLQANLWRSF LYSNVALYLP

B_11_ACZ06785 IVSNGDNAPN -----W-KEP -EVNGTS-EI RQGNLSAMEI NLQANLWRSF LYSNVALYLP

B_55_AIS92536 IVSNGDNAPN -----W-KEP -EVNGTS-EI RQGNLSAMEI NLQANLWRSF LYSNVALYLP

B_21_AAG21823 IEP-NGQGAD -----W-KEP -DINGTS-EI GQGNLFAMEI NLQANLWRSF LYSNVALYLP

B_50_ABA00027 IETNGDETTT -----W-KDL -EPKGIS-EI AKGNPFAMEI NLQANLWRSF LYSNVALYLP

B_14_AAZ99996 IQLNGDQA-- -----W-KDV -NPNGIS-EL VKGNPFAMEI NLQANLWRSF LYSNVALYLP

B_34_AAW33485 IKPNGDQST- -----WTNVD --PTGSS-EL AKGNPFAMEI NLQANLWRSF LYSNVALYLP

B_79_BAW32492 IKPNGDQST- -----WT-NV -DPNGSS-QL AKGNPFAMEI NLQANLWRSF LYSNVALYLP

B_68_AET87230 IKVKTDDTNG -----WEKDA -NVATAN-EI AIGNNLAMEI NIQANLWRNF LYSNVALYLP

B_66_AET87148 IKPRDTA--- -----WEKDT -KVSTAN-EI AIGNNLAMEI NIQANLWRSF LYSNVALYLP

E_4_AAD03660 VKVKTDAGSE K----WDKDD TTVSTAN-EI HVGNPFAMEI NIQANLWRNF LYANVALYLP

B_16_AAW33444 VKVKTDAVAG TSGTQWDKDD TTVSTAN-EI HGGNPFAMEI NIQANLWRSF LYSNVALYLP

A_61_AEK79922 IKQNNGGGGN -----WAADD -TVGDKN-DI GIGNIAAMEI NLQANLWRSF LYSNVGLYLP

A_12_BAG48789 IKPDNGGGGG -----WTADN -TVSEAN-HI GIGNIAAMEI NLQANLWRSF LYSNVGLYLP

A_18_AAZ99994 VKYDANA--- -----WAKDN -NKADNN-NL ATGNIFAMEI NLQANLWRSF LYSNVGLYLP

A_31_CAO78638 VKQNDGNG-- -----WVKDD -SVADTN-DI AKGNIFAMEI NLQANLWRSF LYSNVGLYLP

G_52_ABK35044 IKSNNGSG-- -----WQVDN DTFADRGVEI GSGNMFAMEI NLQANLWRSF LYSNIGLYLP

F_41_ACH90432 IKANGQT--- -----WTADD -NYADRGAEI ESGNIFAMEI NLAANLWRSF LYSNVALYLP

F_40_AMQ95234 VKTDNGTN-- -----WSQNN TDVSSNN-EI SIGNVFAMEI NLAANLWRSF LYSNVALYLP

....|....| ....|....| ....|....| ....|....| ....|....| ....|....|

545 555 565 575 585 595

HexN1 DSYKYTPANV TLPTNTNTYE YMNGRVVAPS LVDAYINIGA RWSLDPMDNV NPFNHHRNAG

B_3_AAW33168 DVYKYTPPNI TLPTNTNTYE YMNGRVVSPS LVDSYINIGA RWSLDPMDNV NPFNHHRNAG

C_5_AAW65514 DKLKYSPSNV KISDNPNTYD YMNKRVVAPG LVDCYINLGA RWSLDYMDNV NPFNHHRNAG

B_7_AAD03663 DVYKYTPTNI TLPANTNTYE YMNGRVVSPS LVDSYINIGA RWSLDPMDNV NPFNHHRNAG

C_2_AAO24095 DKLKYNPTNV EISDNPNTYD YMNKRVVAPG LVDCYINLGA RWSLDYMDNV NPFNHHRNAG

C_1_AAQ10553 DKLKYTPTNV EISPNPNSYD YMNKRVVAPG LVDCYINLGA RWSLDYMDNV NPFNHHRNAG

C_57_ADM46153 DKLKYNPTNV EISDNPNTYD YMNKRVVAPG LVDCYINLGA RWSLDYMDNV NPFNHHRNAG

C_6_ADV03661 DKLKYNPTNV EISDNPNTYD YMNKRVVAPG LVDCYINLGA RWSLDYMDNV NPFNHPRHAG

D_37_ABA00016 DSYKYTPANV TLPANTNTYE YMNGRVVAPS LVDAYINIGA RWSLDPMDNV NPFNHHRNAG

D_13_ABA00000 DSYKYTPANV TLPANTNTYE YMNGRVVAPS LVDAYINIGA RWSLDPMDNV NPFNHHRNAG

D_70_AKI33526 DSYKYTPANV TLPTNTNTYD YMNGRVVAPS LVDAYINIGA RWSLDPMDNV NPFNHHRNAG

D_63_AEV92966 DSYKYTPANV TLPTNTNTYE YMNGRVVAPS LVDAYINIGA RWSLDPMDNV NPFNHHRNAG

D_72_AGT78001 DSFKYTPANV TLPTNTNTYE YMNGRVVAPS LVDAYINIGA RWSLDPMDNV NPFNHHRNAG

D_23_ABA00005 DSFKYTPANV TLPTNTNTYE YMNGRVVAPS LVDAYVNIGA RWSLDPMDNV NPFNHHRNAG

D_24_ABA00006 DSYKYTPANV TLPTNTNTYD YMNGRVVAPS LVDAYINIGA RWSLDPMDNV NPFNHHRNAG

D_39_ABA00018 DSFKYTPANV TLPTNTNTYE YMNGRVVAPS LVDAYINIGA RWSLDPMDNV NPFNHHRNAG

D_43_ABA00020 DSYKYTPANV TLPANTNTYE YMNGRVVAPS LVDAYINIGA RWSLDPMDNV NPFNHHRNAG

D_58_ADW95419 DSYKYTPANI TLPTNTNTYE YMNGRVVAPS LVDAYINIGA RWSLDPMDNV NPFNHHRNAG

D_33_ABA00014 DSYKYTPANV TLPTNTNTYE YMNGRVVAPS LVDAYINIGA RWSLDPMDNV NPFNHHRNAG

D_38_ABA00017 DSYKYTPANV TLPTNTNTYD YINGRVVAPS LVDAYINIGA RWSLDPMNNV NPFNHHRNAG

D_36_ACY04472 DSYKYTPANI TLPTNTNTYE YMNGRVVAPS LVDAYVNIGA RWSLDPMDNV NPFNHHRNAG

D_49_ABA00025 DSYKYTPANV KLPTNTNTYD YMNGRVVAPS LVDAYINIGA RWSLDPMDNV NPFNHHRNAG

D_8_AAZ99998 DSFKYTPANV TLPTNTNTYE YMNGRVAAPS LVDAYVNIGA RWSLDPMDNV NPFNHHRNAG

D_46_ABA00023 DSYKYTPANV TLPANTNTYD YMNGRVVAPS LVDAYVNIGA RWSLDPMDNV NPFNHHRNAG

D_47_ABA00024 DSYKYTPANV TLPANTNTYE YMNGRVVAPS LVDAYVNIGA RWSLDPMDNV NPFNHHRNAG

D_44_ABA00021 DSYKYTPANV TLPTNTNTYE YMNGRVVAPS LVDAYINIGA RWSLDPMDNV NPFNHHRNAG

D_48_ABO61301 DSYKYTPANV TLPTNTNTYE YMNGRVVAPS LVDAYINIGA RWSLDPMDNV NPFNHHRNAG

D_22_ABA00004 DSFKYTPANV TLPTNTNTYE YMNGRVVAPS LVDAYVNIGA RWSLDPMDNV NPFNHHRNAG

D_53_BAJ46350 DSYKYTPANV TLPANTNTYE YMNGRVVAPS LVDAYINIGA RWSLDPMDNV NPFNHHRNAG

D_45_ABA00022 DSYKYTPANV TLPANTNTYE YMNGRVVAPS LVDAYINIGA RWSLDPMDNV NPFNHHRNAG

D_26_ABA00008 DSYKYTPANV KLPANTNTYE YMNGRVVAPS LVDAYINIGA RWSLDPMDNV NPFNHPRNAG

D_32_ABA00013 DSYKYTPANI TLPTNTNTYE YMNGRVVAPS LVDAYINIGA RWSLDPMDNV NPFNHHRNAG

D_73_APD28385 DSYKYTPANV TLPANTNTYE YMNGRVVAPS LVDAYINIGA RWSLDPMDNV NPFNHHRNAG

D_67_BAL63187 DSYKYTPANI TLPTNTNTYE YMNGRVVAPS LVDAYVNIGA RWSLDPMDNV NPFNHHRNAG

D_9_BAE66671 DSYNDTPANI TLPTNTNTYE YMNGHVVAPS LVDAYINIGA RWSLDPMDNV NPFNHHRNAG

D_54_YP_0030386 DSFKYTPANV TLPTNTNTYE YMNGRVVAPS LVDAYVNIGA RWSLDPMDNV NPFNHHRNAG

D_17_ADY18429 DSYKYTPANV TLPANTNTYE YMNGRVVAPS LVDAYINIGA RWSLDPMDNV NPFNHHRNAG

D_10_AAZ99999 DSYKYTPANV TLPANTNTYE YMNGRVVAPW LVDAYINMGA RWSLDPMDNV NPFNHHRNAG

D_65_BAL41721 DSYKYTPANV TLPANTNTYE YMNGRVVAPS LVDAYINIGA RWSLDPMDNV NPFNHHRNAG

D_62_AEL78842 DSYKYTPANV KLPANTNTYE YMNGRVVAPS LVDAYINIGA RWSLDPMDNV NPFNHHRNAG

D_25_ABA00007 DSYKYTPANV KLPTNTNTYE YMNGRVVAPS LVDAYVNIGA RWSLDPMDNV NPFNHHRNAG

D_59_AEI91289 DSYKYTPANV KLPTNTNTYE YMNGRVVAPS LVDAYINIGA RWSLDPMDNV NPFNHHRNAG

D_19_ABA00002 DSYKYTPANV TLPANTNTYE YMNGRVVAPS LVDAYVNIGA RWSLDPMDNV NPFNHHRNAG

D_64_ABN10535 DSYKYTPANI TLPANTNTYE YMNGRVVAPS LVDAYVNIGA RWSLDPMDNV NPFNHHRNAG

D_27_ABA00009 DSFKYTPANV TLPTNTNTYE YMNGRVVAPS LVDAYINIGA RWALDPMDNV NPFNHHRNAG

D_28_ACQ91158 DSYKYTPANV TLPANTNTYE YMNGRVVAPS LVDAYINIGA RWSLDPMDNV NPFNHHRNAG

D_20_ABA00003 DSYKYTPANV TLPANTNTYE YMNGRVVAPS LVDAYINIGA RWSLDPMDNV NPFNHHRNAG

D_60_AEK87026 DSYKYTPANV KLPANTNTYE YMNGRVVAPS LVDAYINIGA RWSLDPMDNV NPFNHHRNAG

D_71_AGT76762 DSYKYTPANV KLPTNTNTYE YMNGRVVAPS LVDAYVNIGA RWSLDPMDNV NPFNHHRNAG

D_42_ABA00019 DSYKYTPANV TLPTNTNTYE YMNGRVVAPS LVDAYINIGA RWSLDPMDNV NPFNHHRNAG

D_51_ABA00026 DSYKYTPANV TLPANTNTYE YMNGRVVAPS LVDAYINIGA RWSLDPMDNV NPFNHHRNAG

D_30_ABA00012 DSYKYTPANV TLPTNTNTYE YMNGRVVAPS LVDAYINIGA RWSLDPMDNV NPFNHHRNAG

D_29_BAJ22326 DSYKYTPANV TLPTNTNTYE YMNGRVVAPS LVDAYINIGA RWSLDPMDNV NPFNHHRNAG

D_56_ADM66117 DSYKYTPANV TLPTNTNTYE YMNGRVVAPS LVDAYINIGA RWSLDPMDNV NPFNHHRNAG

D_69_AFK92217 DSYKYTPANV TLPANTNTYE YMNGRVVAPS LVDAYINIGA RWSLDPMDNV NPFNHHRNAG

D_15_BAJ22290 DSYKYTPANV TLPANTNTYE YMNGRVVAPS LVDAYINIGA RWSLDPMDNV NPFNHHRNAG

B_35_AAP92351 DSYKYTPSNV TLPENKNTYD YMNGRVVPPS LVDTYVNIGA RWSLDAMDNV NPFNHHRNAG

B_11_ACZ06785 DSYKYTPSNV TLPENKNTYD YMNGRVVPPS LVDTYVNIGA RWSLDAMDNV NPFNHHRNAG

B_55_AIS92536 DSYKYTPSNV TLPENKNTYD YMNGRVVPPS LVDTYVNIGA RWSLDAMDNV NPFNHHRNAG

B_21_AAG21823 DSYKYTPANV TLPTNTNTYD YMNGRVVPPS LVDTYVNIGA RWSLDAMDNV NPFNHHRNAG

B_50_ABA00027 DSYKYTPANV TLPTNTNTYD YMNGRVVPPS LVDTYVNIGA RWSLDAMDNV NPFNHHRNAG

B_14_AAZ99996 DSYKYTPSNV TLPENKNTYD YMNGRVVPPS LVDTYVNIGA RWSLDAMDNV NPFNHHRNAG

B_34_AAW33485 DSYKYTPSNV TLPENKNTYD YMNGRVVPPS LVDTYVNIGA RWSLDAMDNV NPFNHHRNAG

B_79_BAW32492 DSYKYTPSNV TLPENKNTYD YMNGRVVPPS LVDTYVNIGA RWSLDAMDNV NPFNHHRNAG

B_68_AET87230 DVYKYTPPNI TLPANTNTYE YMNGRVVSPS LVDSYINIGA RWSLDPMDNV NPFNHHRNAG

B_66_AET87148 DVYKYTPTNI TLPANTNTYE YMNGRVVSPS LVDSYINIGA RWSLDPMDNV NPFNHHRNAG

E_4_AAD03660 DKYKYTPANI TLPTNTNTYE YMNGRVVAPS LVDAYINIGA RWSLDPMDNV NPFNHHRNAG

B_16_AAW33444 DSYKYTPSNV TLPENKNTYD YMNGRVVPPS LVDTYVNIGA RWSLDAMDNV NPFNHHRNAG

A_61_AEK79922 DDLKYTPGNI KLPENKNTYE YMNGRVTVPG LVDTYVNIGA RWSPDVMDNI NPFNHHRNAG

A_12_BAG48789 DDLKYTPGNI KLPDNKNTYE YMNGRVTAPG LVDTYVNIGA RWSPDVMDNV NPFNHHRNAG

A_18_AAZ99994 DSLKYTPGNI KLPDNKNTYE YMNGRVTAPG LVDTYVNIGA RWSPDVMDNI NPFNHHRNAG

A_31_CAO78638 DDLKYTPGNI KLPENKNTYE YMNGRVTVPG LVDTYVNIGA RWSPDVMDNI NPFNHHRNAG

G_52_ABK35044 DSLKLTPDNI TLPENKNTYQ YMNGRVTPPG LVDTYVNVGA RWSPDVMDSI NPFNHHRNAG

F_41_ACH90432 DSYKITPDNI TLPENKNTYA YMNGRVAVPS ALDTYVNIGA RWSPDPMDNV NPFNHHRNAG

F_40_AMQ95234 DSYKITPDNI TLPDNKNTYA YMNGRVAVPS ALDTYVNIGA RWSPDPMDNV NPFNHHRNAG

....|....| ....|....| ....|....| ....|....| ....|....| ....|....|

605 615 625 635 645 655

HexN1 LRYRSMLLGN GRYVPFHIQV PQKFFAIKNL LLLPGSYTYE WNFRKDVNMI LQSSLGNDLR

B_3_AAW33168 LRYRSMLLGN GRYVPFHIQV PQKFFAVKNL LLLPGSYTYE WNFRKDVNMV LQSSLGNDLR

C_5_AAW65514 LRYRSMLLGN GRYVPFHIQV PQKFFAIKNL LLLPGSYTYE WNFRKDVNMV LQSSLGNDLR

B_7_AAD03663 LRYRSMLLGN GRYVPFHIQV PQKFFAVKNL LLLPGSYTYE WNFRKDVNMV LQSSLGNDLR

C_2_AAO24095 LRYRSMLLGN GRYVPFHIQV PQKFFAIKNL LLLPGSYTYE WNFRKDVNMV LQSSLGNDLR

C_1_AAQ10553 LRYRSMLLGN GRYVPFHIQV PQKFFAIKNL LLLPGSYTYE WNFRKDVNMV LQSSLGNDLR

C_57_ADM46153 LRYRSMLLGN GRYVPFHIQV PQKFFAIKNL LLLPGSYTYE WNFRKDVNMV LQSSLGNDLR

C_6_ADV03661 LRYRSMLLGN GRYVPFHIQV PQKFFAIKNL LLLPGSYTYE WNFRKDVNMV LQSSLGNDLR

D_37_ABA00016 LRYRSMLLGN GRYVPFHIQV PQKFFAIKNL LLLPGSYTYE WNFRKDVNMI LQSSLGNDLR

D_13_ABA00000 LRYRSMLLGN GRYVPFHIQV PQKFFAIKNL LLLPGSYTYE WNFRKDVNMI LQSSLGNDLR

D_70_AKI33526 LRYRSMLLGN GRYVPFHIQV PQKFFAIKNL LLLPGSYTYE WNFRKDVNMI LQSSLGNDLR

D_63_AEV92966 LRYRSMLLGN GRYVPFHIQV PQKFFAIKNL LLLPGSYTYE WNFRKDVNMI LQSSLGNDLR

D_72_AGT78001 LRYRSMLLGN GRYVPFHIQV PQKFFAIKNL LLLPGSYTYE WNFRKDVNMI LQSSLGNDLR

D_23_ABA00005 LRYRSMLLGN GRYVPFHIQV PQKFFAIKNL LLLPGSYTYE WNFRKDVNMI LQSSLGNDLR

D_24_ABA00006 LRYRSMLLGN GRYVPFHIQV PQKFFAIKNL LLLPGSYTYE WNFRKDVNMI LQSSLGNDLR

D_39_ABA00018 LRYRSMLLGN GRYVPFHIQV PQKFFAIKNL LLLPGSYTYE WNFRKDVNMI LQSSLGNDLR

D_43_ABA00020 LRYRSMLLGN GRYVPFHIQV PQKFFAIKNL LLLPGSYTYE WNFRKDVNMI LQSSLGNDLR

D_58_ADW95419 LRYRSMLLGN GRYVPFHIQV PQKFFAIKNL LLLPGSYTYE WNFRKDVNMI LQSSLGNDLR

D_33_ABA00014 LRYRSMLLGN GRYVPFHIQV PQKFFAIKNL LLLPGSYTYE WNFRKDVNMI LQSSLGNDLR

D_38_ABA00017 LRYRSMLLGN GRYVPFHIQV PQKFFAIKNL LLLPGSYTYE WNFRKDVNMI LQSSLGNDLR

D_36_ACY04472 LRYRSMLLGN GRYVPFHIQV PQKFFAIKNL LLLPGSYTYE WNFRKDVNMI LQSSLGNDLR

D_49_ABA00025 LRYRSMLLGN GRYVPFHIQV PQKFFAIKNL LLLPGSYTYE WNFRKDVNMI LQSSLGNDLR

D_8_AAZ99998 LRYRSMLLGN GRYVPFHIQV PQKFFAIKNL LLLPGSYTYE WNFRKDVNMI LQSSLGNDLR

D_46_ABA00023 LRYRSMLLGN GRYVPFHIQV PQKFFAIKNL LLLPGSYTYE WNFRKDVNMI LQSSLGNDLR

D_47_ABA00024 LRYRSMLLGN GRYVPFHIQV PQKFFAIKNL LLLPGSYTYE WNFRKDVNMI LQSSLGNDLR

D_44_ABA00021 LRYRSMLLGN GRYVPFHIQV PQKFFAIKNL LLLPGSYTYE WNFRKDVNMI LQSSLGNDLR

D_48_ABO61301 LRYRSMLLGN GRYVPFHIQV PQKFFAIKNL LLLPGSYTYE WNFRKDVNMI LQSSLGNDLR

D_22_ABA00004 LRYRSMLLGN GRYVPFHIQV PQKFFAIKNL LLLPGSYTYE WNFRKDVNMI LQSSLGNDLR

D_53_BAJ46350 LRYRSMLLGN GRYVPFHIQV PQKFFAIKNL LLLPGSYTYE WNFRKDVNMI LQSSLGNDLR

D_45_ABA00022 LRYRSMLLGN GRYVPFHIQV PQKFFAIKNL LLLPGSYTYE WNFRKDVNMI LQSSLGNDLR

D_26_ABA00008 LRYRSMLLGN GRYVPFHIQV PQKFFAIKNL LLLPGSYTYE WNFRKDVNMI LQSSLGNDLR

D_32_ABA00013 LRYRSMLLGN GRYVPFHIQV PQKFFAIKNL LLLPGSYTYE WNFRKDVNMI LQSSLGNDLR

D_73_APD28385 LRYRSMLLGN GRYVPFHIQV PQKFFAIKNL LLLPGSYTYE WNFRKDVNMI LQSSLGNDLR

D_67_BAL63187 LRYRSMLLGN GRYVPFHIQV PQKFFAIKNL LLLPGSYTYE WNFRKDVNMI LQSSLGNDLR

D_9_BAE66671 LAYRSMLLGN GRYVPFHIQV PQKFFAIKNL LLLPGSYTYE WNFRKDVNMI LQSSLGNDLR

D_54_YP_0030386 LRYRSMLLGN GRYVPFHIQV PQKFFAIKNL LLLPGSYTYE WNFRKDVNMI LQSSLGNDLR

D_17_ADY18429 LRYRSMLLGN GRYVPFHIQV PQKFFAIKNL LLLPGSYTYE WNFRKDVNMI LQSSLGNDLR

D_10_AAZ99999 LRYRSMLLGN GRYVPFHIQV PQKFFAIKNL LLLPGSYTYE WNFRKDVNMI LQSSLGNDLR

D_65_BAL41721 LRYRSMLLGN GRYVPFHIQV PQKFFAIKNL LLLPGSYTYE WNFRKDVNMI LQSSLGNDLR

D_62_AEL78842 LRYRSMLLGN GRYVPFHIQV PQKFFAIKNL LLLPGSYTYE WNFRKDVNMI LQSSLGNDLR

D_25_ABA00007 LRYRSMLLGN GRYVPFHIQV PQKFFAIKNL LLLPGSYTYE WNFRKDVNMI LQSSLGNDLR

D_59_AEI91289 LRYRSMLLGN GRYVPFHIQV PQKFFAIKNL LLLPGSYTYE WNFRKDVNMI LQSSLGNDLR

D_19_ABA00002 LRYRSMLLGN GRYVPFHIQV PQKFFAIKNL LLLPGSYTYE WNFRKDVNMI LQSSLGNDLR

D_64_ABN10535 LRYRSMLLGN GRYVPFHIQV PQKFFAIKNL LLLPGSYTYE WNFRKDVNMI LQSSLGNDLR

D_27_ABA00009 LRYRSMLLGN GRYVPFHIQV PQKFFAIKNL LLLPGSYTYE WNFRKDVNMI LQSSLGNDLR

D_28_ACQ91158 LRYRSMLLGN GRYVPFHIQV PQKFFAIKNL LLLPGSYTYE WNFRKDVNMI LQSSLGNDLR

D_20_ABA00003 LRYRSMLLGN GRYVPFHIQV PQKFFAIKNL LLLPGSYTYE WNFRKDVNMI LQSSLGNDLR

D_60_AEK87026 LRYRSMLLGN GRYVPFHIQV PQKFFAIKNL LLLPGSYTYE WNFRKDVNMI LQSSLGNDLR

D_71_AGT76762 LRYRSMLLGN GRYVPFHIQV PQKFFAIKNL LLLPGSYTYE WNFRKDVNMI LQSSLGNDLR

D_42_ABA00019 LRYRSMLLGN GRYVPFHIQV PQKFFAIKNL LLLPGSYTYE WNFRKDVNMI LQSSLGNDLR

D_51_ABA00026 LRYRSMLLGN GRYVPFHIQV PQKFFAIKNL LLLPGSYTYE WNFRKDVNMI LQSSLGNDLR

D_30_ABA00012 LRYRSMLLGN GRYVPFHIQV PQKFFAIKNL LLLPGSYTYE WNFRKDVNMI LQSSLGNDLR

D_29_BAJ22326 LRYRSMLLGN GRYVPFHIQV PQKFFAIKNL LLLPGSYTYE WNFRKDVNMI LQSSLGNDLR

D_56_ADM66117 LRYRSMLLGN GRYVPFHIQV PQKFFAIKNL LLLPGSYTYE WNFRKDVNMI LQSSLGNDLR

D_69_AFK92217 LRYRSMLLGN GRYVPFHIQV PQKFFAIKNL LLLPGSYTYE WNFRKDVNMI LQSSLGNDLR

D_15_BAJ22290 LRYRSMLLGN GRYVPFHIQV PQKFFAIKNL LLLPGSYTYE WNFRKDVNMI LQSSLGNDLR

B_35_AAP92351 LRYRSMLLGN GRYVPFHIQV PQKFFAVKNL LLLPGSYTYE WNFRKDVNMV LQSSLGNDLR

B_11_ACZ06785 LRYRSMLLGN GRYVPFHIQV PQKFFAVKNL LLLPGSYTYE WNFRKDVNMV LQSSLGNDLR

B_55_AIS92536 LRYRSMLLGN GRYVPFHIQV PQKFFAVKNL LLLPGSYTYE WNFRKDVNMV LQSSLGNDLR

B_21_AAG21823 LRYRSMLLGN GRYVPFHIQV PQKFFAVKNL LLLPGSYTYE WNFRKDVNMV LQSSLGNDLR

B_50_ABA00027 LRYRSMLLGN GRYVPFHIQV PQKFFAVKNL LLLPGSYTYE WNFRKDVNMV LQSSLGNDLR

B_14_AAZ99996 LRYRSMLLGN GRYVPFHIQV PQKFFAVKNL LLLPGSYTYE WNFRKDVNMV LQSSLGNDLR

B_34_AAW33485 LRYRSMLLGN GRYVPFHIQV PQKFFAVKNL LLLPGSYTYE WNFRKDVNMV LQSSLGNDLR

B_79_BAW32492 LRYRSMLLGN GRYVPFHIQV PQKFFAVKNL LLLPGSYTYE WNFRKDVNMV LQSSLGNDLR

B_68_AET87230 LRYRSMLLGN GRYVPFHIQV PQKFFAVKNL LLLPGSYTYE WNFRKDVNMV LQSSLGNDLR

B_66_AET87148 LRYRSMLLGN GRYVPFHIQV PQKFFAVKNL LLLPGSYTYE WNFRKDVNMV LQSSLGNDLR

E_4_AAD03660 LRYRSMLLGN GRYVPFHIQV PQKFFAIKNL LLLPGSYTYE WNFRKDVNMI LQSSLGNDLR

B_16_AAW33444 LRYRSMLLGN GRYVPFHIQV PQKFFAVKNL LLLPGSYTYE WNFRKDVNMV LQSSLGNDLR

A_61_AEK79922 LRYRSMLLGN GRFVPFHIQV PQKFFAIKNL LLLPGSYTYE WNFRKDVNMI LQSTLGNDLR

A_12_BAG48789 LRYRSMLLGN GRFVPFHIQV PQKFFAIRNL LLLPGSYTYE WNFRKDVNMI LQSTLGNDLR

A_18_AAZ99994 LRYRSMLLGN GRFVPFHIQV PQKFFAIRNL LLLPGSYTYE WNFRKDVNMI LQSTLGNDLR

A_31_CAO78638 LRYRSMLLGN GRFVPFHIQV PQKFFAIKNL LLLPGSYTYE WNFRKDVNMI LQSTLGNDLR

G_52_ABK35044 LRYRSMLLGN GRYVPFHIQV PQKFFAIKNL LLLPGSYTYE WNFRKDVNMI LQSSLGNDLR

F_41_ACH90432 LRYRSMLLGN GRYVPFHIQV PQKFFAIKNL LLLPGSYTYE WNFRKDVNMI LQSSLGNDLR

F_40_AMQ95234 LRYRSMLLGN GRYVPFHIQV PQKFFAIKNL LLLPGSYTYE WNFRKDVNMI LQSSLGNDLR

....|....| ....|....| ....|....| ....|....| ....|....| ....|....|

665 675 685 695 705 715

HexN1 VDGASVRFDS VNLYATFFPM AHNTASTLEA MLRNDTNDQS FNDYLSAANM LYPIPAKATN

B_3_AAW33168 TDGATISFTS INLYATFFPM AHNTASTLEA MLRNDTNDQS FNDYLSAANM LYPIPANATN

C_5_AAW65514 VDGASIKFDS ICLYATFFPM AHNTASTLEA MLRNDTNDQS FNDYLSAANM LYPIPANATN

B_7_AAD03663 TDGATISFTS INLYATFFPM AHNTASTLEA MLRNDTNDQS FNDYLSAANM LYPIPANATN

C_2_AAO24095 VDGASIKFDS ICLYATFFPM AHNTASTLEA MLRNDTNDQS FNDYLSTANM LYPIPANATN

C_1_AAQ10553 VDGASIKFDS ICLYATFFPM AHNTASTLEA MLRNDTNDQS FNDYLSAANM LYPIPANATN

C_57_ADM46153 VDGASIKFDS ICLYATFFPM AHNTASTLEA MLRNDTNDQS FNDYLSAANM LYPIPANATN

C_6_ADV03661 VDGASIKFDS ICLYATFFPM AHNTASTLEA MLRNDTNDQS FNDYLSAANM LYPIPANATN

D_37_ABA00016 VDGASVRFDS VNLYATFFPM AHNTASTLEA MLRNDTNDQS FNDYLSAANM LYPIPAKATN

D_13_ABA00000 VDGASVRFDS VNLYATFFPM AHNTASTLEA MLRNDTNDQS FNDYLSAGNM LYPIPAKATN

D_70_AKI33526 VDGASVRFDS VNLYATFFPM AHNTASTLEA MLRNDTNDQS FNDYLSAANM LYPIPAKATN

D_63_AEV92966 VDGASVRFDS VNLYATFFPM AHNTASTLEA MLRNDTNDQS FNDYLSAANM LYPIPAKATN

D_72_AGT78001 VDGASVRFDS VNLYATFFPM AHNTASTLEA MLRNDTNDQS FNDYLSAANM LYPIPAKATN

D_23_ABA00005 VDGASVRFDS VNLYATFFPM AHNTASTLEA MLRNDTNDQS FNDYLSAANM LYPIPAKATN

D_24_ABA00006 VDGASVRFDS VNLYATFFPM AHNTASTLEA MLRNDTNDQS FNDYLSAANM LYPIPAKATN

D_39_ABA00018 VDGASVRFDS VNLYATFFPM AHNTASTLEA MLRNDTNDQS FNDYLSAANM LYPIPAKATN

D_43_ABA00020 VDGASVRFDS VNLYATFFPM AHNTASTLEA MLRNDTNDQS FNDYLSAANM LYPIPAKATN

D_58_ADW95419 VDGASVRFDS VNLYATFFPM AHNTASTLEA MLRNDTNDQS FNDYLSAANM LYPIPAKATN

D_33_ABA00014 VDGASVRFDS VNLYATFFPM AHNTASTLEA MLRNDTNDQS FNDYLSAANM LYPIPAKATN

D_38_ABA00017 VDGASVRFDS VNLYATFFPM AHNTASTLEA MLRNDTNDQS FNDYLSAANM LYPIPAKATN

D_36_ACY04472 VDGASVRFDS VNLYATFFPM AHNTASTLEA MLRNDTNDQS FNDYLSAANM LYPIPAKATN

D_49_ABA00025 VDGASVRFDS VNLYATFFPM AHNTASTLEA MLRNDTNDQS FNDYLSAANM LYPIPAKATN

D_8_AAZ99998 VDGASVRFDS VNLYATFFPM AHNTASTLEA MLRNDTNDQS FNDYLSAANM LYPIPAKATN

D_46_ABA00023 VDGASVRFDS VNLYATFFPM AHNTASTLEA MLRNDTNDQS FNDYLSGANM LYPIPAKATN

D_47_ABA00024 VDGASVRFDS VNLYATFFPM AHNTASTLEA MLRNDTNDQS FNDYLSAANM LYPIPAKATN

D_44_ABA00021 VDGASVRFDS VNLYATFFPM AHNTASTLEA MLRNDTNDQS FNDYLSAANM LYPIPAKATN

D_48_ABO61301 VDGASVRFDS VNLYATFFPM AHNTASTLEA MLRNDTNDQS FNDYLSAANM LYPIPAKATN

D_22_ABA00004 VDGASVRFDS VNLYATFFPM AHNTASTLEA MLRNDTNDQS FNDYLSAANM LYPIPAKATN

D_53_BAJ46350 VDGASVRFDS VNLYATFFPM AHNTASTLEA MLRNDTNDQS FNDYLSAANM LYPIPAKATN

D_45_ABA00022 VDGASVRFDS VNLYATFFPM AHNTASTLEA MLRNDTNDQS FNDYLSAANM LYPIPAKATN

D_26_ABA00008 VDGASVRFDS VNLYATFFPM AHNTASTLEA MLRNDTHDQS FNDYLSAANM LYPIPAKATN

D_32_ABA00013 VDGASVRFDS VNLYATFFPM AHNTASTLEA MLRNDTNDQS FNDYLSAANM LYPIPAKATN

D_73_APD28385 VDGASVRFDS VNLYATFFPM AHNTASTLEA MLRNDTNDQS FNDYLSAANM LYPIPAKATN

D_67_BAL63187 VDGASVRFDS VNLYATFFPM AHNTASTLEA MLRNDTNDQS FNDYLSAANM LYPIPAKATN

D_9_BAE66671 VDGASVRFDS VNLYATFFPM AHNTASTLEA MLRNDTNDQS FNDYLSAANM LYPIPAKATN

D_54_YP_0030386 VDGASVRFDS VNLYATFFPM AHNTASTLEA MLRNDTNDQS FNDYLSAANM LYPIPAKATN

D_17_ADY18429 VDGASVRFDS VNLYATFFPM AHNTASTLEA MLRNDTNDQS FNDYLSAANM LYPIPAKATN

D_10_AAZ99999 VDGASVRFDS VNLYATFFPM AHNTASTLEA MLRNDTNDQS FNDYLSAANM LYPIPAKATN

D_65_BAL41721 VDGASVRFDS VNLYATFFPM AHNTASTLEA MLRNDTNDQS FNDYLSAANM LYPIPAKATN

D_62_AEL78842 VDGASVRFDS VNLYATFFPM AHNTASTLEA MLRNDTNDQS FNDYLSAANM LYPIPAKATN

D_25_ABA00007 VDGASVRFDS INLYATFFPM AHNTASTLEA MLRNDTNDQS FNDYLSAANM LYPIPAKATN

D_59_AEI91289 VDGASVRFDS VNLYATFFPM AHNTASTLEA MLRNDTNDQS FNDYLSAANM LYPIPAKATN

D_19_ABA00002 VDGASVRFDS VNLYATFFPM AHNTASTLEA MLRNDTNDQS FNDYLSAANM LYPIPAKATN

D_64_ABN10535 VDGASVRFDS VNLYATFFPM AHNTASTLEA MLRNDTNDQS FNDYLSAANM LYPIPAKATN

D_27_ABA00009 VDGASVRFDS VNLYATFFPM AHNTASTLEA MLRNDTNDQS FNDYLSAANM LYPIPAKATN

D_28_ACQ91158 VDGASVRFDS VNLYATFFPM AHNTASTLEA MLRNDTNDQS FNDYLSAANM LYPIPAKATN

D_20_ABA00003 VDGASVRFDS VNLYATFFPM AHNTASTLEA MLRNDTNDQS FNDYLSAANM LYPIPAKATN

D_60_AEK87026 VDGASVRFDS VNLYATFFPM AHNTASTLEA MLRNDTNDQS FNDYLSAANM LYPIPAKATN

D_71_AGT76762 VDGASVRFDS VNLYATFFPM AHNTASTLEA MLRNDTNDQS FNDYLSAANM LYPIPAKATN

D_42_ABA00019 VDGASVRFDS VNLYATFFPM AHNTASTLEA MLRNDTNDQS FNDYLSAANM LYPIPAKATN

D_51_ABA00026 VDGASVRFDS VNLYATFFPM AHNTASTLEA MLRNDTNDQS FNDYLSAANM LYPIPAKATN

D_30_ABA00012 VDGASVRFDS VNLYATFFPM AHNTASTLEA MLRNDTNDQS FNDYLSAANM LYPIPAKATN

D_29_BAJ22326 VDGASVRFDS VNLYATFFPM AHNTASTLEA MLRNDTNDQS FNDYLSAANM LYPIPAKATN

D_56_ADM66117 VDGASVRFDS VNLYATFFPM AHNTASTLEA MLRNDTNDQS FNDYLSAANM LYPIPAKATN

D_69_AFK92217 VDGASVRFDS VNLYATFFPM AHNTASTLEA MLRNDTNDQS FNDYLSAANM LYPIPAKATN

D_15_BAJ22290 VDGASVRFDS VNLYATFFPM AHNTASTLEA MLRNDTNDQS FNDYLSAANM LYPIPAKATN

B_35_AAP92351 VDGASISFTS INLYATFFPM AHNTASTLEA MLRNDTNDQS FNDYLSAANM LYPIPANATN

B_11_ACZ06785 VDGASISFTS INLYATFFPM AHNTASTLEA MLRNDTNDQS FNDYLSAANM LYPIPANATN

B_55_AIS92536 VDGASISFTS INLYATFFPM AHNTASTLEA MLRNDTNDQS FNDYLSAANM LYPIPANATN

B_21_AAG21823 VDGASISFTS INLYATFFPM AHNTASTLEA MLRNDTNDQS FNDYLSAANM LYPIPANATN

B_50_ABA00027 VDGASISFTS INLYATFFPM AHNTASTLEA MLRNDTNDQS FNDYLSAANM LYPIPANATN

B_14_AAZ99996 VDGASISFTS INLYATFFPM AHNTASTLEA MLRNDTNDQS FNDYLSAANM LYPIPANATN

B_34_AAW33485 VDGASISFTS INLYATFFPM AHNTASTLEA MLRNDTNDQS FNDYLSAANM LYPIPANATN

B_79_BAW32492 VDGASISFTS INLYATFFPM AHNTASTLEA MLRNDTNDQS FNDYLSAANM LYPIPANATN

B_68_AET87230 TDGATISFTS INLYATFFPM AHNTASTLEA MLRNDTNDQS FNDYLSAANM LYPIPANATN

B_66_AET87148 TDGATISFTS INLYATFFPM AHNTASTLEA MLRNDTNDQS FNDYLSAANM LYPIPANATN

E_4_AAD03660 TDGASITFTS INLYATFFPM AHNTASTLEA MLRNDTNDQS FNDYLSAANM LYPIPANATN

B_16_AAW33444 VDGASISFTS INLYATFFPM AHNTASTLEA MLRNDTNDQS FNDYLSAANM LYPIPANATN

A_61_AEK79922 VDGASIRFDN IALYANFFPM AHNTASTLEA MLRNDTNDQS FNDYLCAANM LYPIPANATS

A_12_BAG48789 VDGASVRFDN IALYANFFPM AHNTASTLEA MLRNDTNDQS FNDYLCAANM LYPIPANATS

A_18_AAZ99994 VDGASIRFDN IALYANFFPM AHNTASTLEA MLRNDTNDQS FNDYLCAANM LYPIPANATS

A_31_CAO78638 VDGASIRFDN IALYANFFPM AHNTASTLEA MLRNDTNDQS FNDYLCAANM LYPIPANATS

G_52_ABK35044 VDGASIRFDS INLYANFFPM AHNTASTLEA MLRNDTNDQS FNDYLCAANM LYPIPANATS

F_41_ACH90432 VDGASVRFDS INLYANFFPM AHNTASTLEA MLRNDTNDQS FNDYLCAANM LYPIPSNATS

F_40_AMQ95234 VDGASVRFDS INLYANFFPM AHNTASTLEA MLRNDTNDQS FNDYLCAANM LYPIPANATS

....|....| ....|....| ....|....| ....|....| ....|....| ....|....|

725 735 745 755 765 775

HexN1 VPISIPSRNW AAFRGWSFTR LKTKETPSLG SGFDPYFVYS GSIPYLDGTF YLNHTFKKVS

B_3_AAW33168 IPISIPSRNW AAFRGWSFTR LKTKETPSLG SGFDPYFVYS GSIPYLDGTF YLNHTFKKVS

C_5_AAW65514 VPISIPSRNW AAFRGWAFTR LKTKETPSLG SGYDPYYTYS GSIPYLDGTF YLNHTFKKVA

B_7_AAD03663 IPISIPSRNW AAFRGWSFTR LKTKETPSLG SGFDPYFVYS GSIPYLDGTF YLNHTFKKVS

C_2_AAO24095 VPISIPSRNW AAFRGWAFTR LKTKETPSLG SGYDPYYTYS GSIPYLDGTF YLNHTFKKVA

C_1_AAQ10553 VPISIPSRNW AAFRGWAFTR LKTKETPSLG SGYDPYYTYS GSIPYLDGTF YLNHTFKKVA

C_57_ADM46153 VPISIPSRNW AAFRGWAFTR LKTKETPSLG SGYDPYYTYS GSIPYLDGTF YLNHTFKKVA

C_6_ADV03661 VPISIPSRNW AAFRGWAFTR LKTKETPSLG SGYDPYYTYS GSIPYLDGTF YLNHTFKKVA

D_37_ABA00016 VPISIPSRNW AAFRGWSFTR LKTKETPSLG SGFDPYFVYS GSIPYLDGTF YLNHTFKKVS

D_13_ABA00000 VPISIPSRNW AAFRGWSFTR LKTKETPSLG SGFDPYFVYS GSIPYLDGTF YLNHTFKKVS

D_70_AKI33526 VPISIPSRNW AAFRGWSFTR LKTKETPSLG SGFDPYFVYS GSIPYLDGTF YLNHTFKKVS

D_63_AEV92966 VPISIPSRNW AAFRGWSFTR LKTKETPSLG SGFDPYFVYS GSIPYLDGTF YLNHTFKKVS

D_72_AGT78001 VPISIPSRNW AAFRGWSFTR LKTKETPSLG SGFDPYFVYS GSIPYLDGTF YLNHTFKKVS

D_23_ABA00005 VPISIPSRNW AAFRGWSFTR LKTKETPSLG SGFDPYFVYS GSIPYLDGTF YLNHTFKKVS

D_24_ABA00006 VPISIPSRNW AAFRGWSFTR LKTKETPSLG SGFDPYFVYS GSIPYLDGTF YLNHTFKKVS

D_39_ABA00018 VPISIPSRNW AAFRGWSFTR LKTKETPSLG SGFDPYFVYS GSIPYLDGTF YLNHTFKKVS

D_43_ABA00020 VPISIPSRNW AAFRGWSFTR LKTKETPSLG SGFDPYFVYS GSIPYLDGTF YLNHTFKKVS

D_58_ADW95419 VPISIPSRNW AAFRGWSFTR LKTKETPSLG SGFDPYFVYS GSIPYLDGTF YLNHTFKKVS

D_33_ABA00014 VPISIPSRNW AAFRGWSFTR LKTKETPSLG SGFDPYFVYS GSIPYLDGTF YLNHTFKKVS

D_38_ABA00017 VPISIPSRNW AAFRGWSFTR LKTKETPSLG SGFDPYFVYS GSIPYLDGTF YLNHTFKKVS

D_36_ACY04472 VPISIPSRNW AAFRGWSFTR LKTKETPSLG SGFDPYFVYS GSIPYLDGTF YLNHTFKKVS

D_49_ABA00025 VPISIPSRNW AAFRGWSFTR LKTKETPSLG SGFDPYFVYS GSIPYLDGTF YLNHTFKKVS

D_8_AAZ99998 VPISIPSRNW AAFRGWSFTR LKTKETPSLG SGFDPYFVYS GTIPYLDGTF YLNHTFKKVS

D_46_ABA00023 VPISIPSRNW AAFRGWSFTR LKTKETPSLG SGFDPYFVYS GSIPYLDGTF YLNHTFKKVS

D_47_ABA00024 VPISIPSRNW AAFRGWSFTR LKTKETPSLG SGFDPYFVYS GSIPYLDGTF YLNHTFKKVS

D_44_ABA00021 VPISIPSRNW AAFRGWSFTR LKTKETPSLG SGFDPYFVYS GSIPYLDGTF YLNHTFKKVS

D_48_ABO61301 VPISIPSRNW AAFRGWSFTR LKTKETPSLG SGFDPYFVYS GSIPYLDGTF YLNHTFKKVS

D_22_ABA00004 VPISIPSRNW AAFRGWSFTR LKTKETPSLG SGFDPYFVYS GSIPYLDGTF YLNHTFKKVS

D_53_BAJ46350 VPISIPSRNW AAFRGWSFTR LKTKETPSLG SGFDPYFVYS GSIPYLDGTF YLNHTFKKVS

D_45_ABA00022 VPISIPSRNW AAFRGWSFTR LKTKETPSLG SGFDPYFVYS GSIPYLDGTF YLNHTFKKVS

D_26_ABA00008 VPISIPSRNW AAFRGWSFTR LKTKETPSLG SGFDPYFVYS GSIPYLDGTF YLNHTFKKVS

D_32_ABA00013 VPISIPSRNW AAFRGWSFTR LKTKETPSLG SGFDPYFVYS GSIPYLDGTF YLNHTFKKVS

D_73_APD28385 VPISIPSRNW AAFRGWSFTR LKTKETPSLG SGFDPYFVYS GSIPYLDGTF YLNHTFKKVS

D_67_BAL63187 VPISIPSRNW AAFRGWSFTR LKTKETPSLG SGFDPYFVYS GSIPYLDGTF YLNHTFKKVS

D_9_BAE66671 VPISIPPRNW AAFRGWSFTR LKTKETPSLG SGFDPYFVYS GSIPYLDGTF YLNHTFKKVS

D_54_YP_0030386 VPISIPSRNW AAFRGWSFTR LKTKETPSLG SGFDPYFVYS GSIPYLDGTF YLNHTFKKVS

D_17_ADY18429 VPISIPSRNW AAFRGWSFTR LKTKETPSLG SGFDPYFVYS GSIPYLDGTF YLNHTFKKVS

D_10_AAZ99999 VPISIPSRNW AAFRGWSFTR LKTKETPSLG SGFDPYFVYS GSIPYLDGTF YLNHTFKKVS

D_65_BAL41721 VPISIPSRNW AAFRGWSFTR LKTKETPSLG SGFDPYFVYS GSIPYLDGTF YLNHTFKKVS

D_62_AEL78842 VPISIPSRNW AAFRGWSFTR LKTKETPSLG SGFDPYFVYS GSIPYLDGTF YLNHTFKKVS

D_25_ABA00007 VPISIPSRNW AAFRGWSFTR LKTKETPSLG SGFDPYFVYS GSIPYLDGTF YLNHTFKKVS

D_59_AEI91289 VPISIPSRNW AAFRGWSFTR LKTKETPSLG SGFDPYFVYS GSIPYLDGTF YLNHTFKKVS

D_19_ABA00002 VPISIPSRNW AAFRGWSFTR LKTKETPSLG SGFDPYFVYS GSIPYLDGTF YLNHTFKKVS

D_64_ABN10535 VPISIPSRNW AAFRGWSFTR LKTKETPSLG SGFDPYFVYS GSIPYLDGTF YLNHTFKKVS

D_27_ABA00009 VPISIPSRNW AAFRGWSFTR LKTKETPSLG SGFDPYFVYS GSIPYLDGTF YLNHTFKKVS

D_28_ACQ91158 VPISIPSRNW AAFRGWSFTR LKTKETPSLG SGFDPYFVYS GSIPYLDGTF YLNHTFKKVS

D_20_ABA00003 VPISIPSRNW AAFRGWSFTR LKTKETPSLG SGFDPYFVYS GSIPYLDGTF YLNHTFKKVS

D_60_AEK87026 VPISIPSRNW AAFRGWSFTR LKTKETPSLG SGFDPYFVYS GSIPYLDGTF YLNHTFKKVS

D_71_AGT76762 VPISIPSRNW AAFRGWSFTR LKTKETPSLG SGFDPYFVYS GSIPYLDGTF YLNHTFKKVS

D_42_ABA00019 VPISIPSRNW AAFRGWSFTR LKTKETPSLG SGFDPYFVYS GSIPYLDGTF YLNHTFKKVS

D_51_ABA00026 VPISIPSRNW AAFRGWSFTR LKTKETPSLG SGFDPYFVYS GSIPYLDGTF YLNHTFKKAS

D_30_ABA00012 VPISIPSRNW AAFRGWSFTR LKTKETPSLG SGFDPYFVYS GSIPYLDGTF YLNHTFKKVS

D_29_BAJ22326 VPISIPSRNW AAFRGWSFTR LKTKETPSLG SGFDPYFVYS GSIPYLDGTF YLNHTFKKVS

D_56_ADM66117 VPISIPSRNW AAFRGWSFTR LKTKETPSLG SGFDPYFVYS GSIPYLDGTF YLNHTFKKVS

D_69_AFK92217 VPISIPSRNW AAFRGWSFTR LKTKETPSLG SGFDPYFVYS GSIPYLDGTF YLNHTFKKVS

D_15_BAJ22290 VPISIPSRNW AAFRGWSFTR LKTKETPSLG SGFDPYFVYS GSIPYLDGTF YLNHTFKKVS

B_35_AAP92351 IPISIPSRNW AAFRGWSFTR LKTKETPSLG SGFDPYFVYS GSIPYLDGTF YLNHTFKKVS

B_11_ACZ06785 IPISIPSRNW AAFRGWSFTR LKTKETPSLG SGFDPYFVYS GSIPYLDGTF YLNHTFKKVS

B_55_AIS92536 IPISIPSRNW AAFRGWSFTR LKTKETPSLG SGFDPYFVYS GSIPYLDGTF YLNHTFKKVS

B_21_AAG21823 VPISIPSRNW AAFRGWSFTR LKTKETPSLG SGFDPYFVYS GSIPYLDGTF YLNHTFKKVS

B_50_ABA00027 VPISIPSRNW AAFRGWSFTR LKTKETPSLG SGFDPYFVYS GSIPYLDGTF YLNHTFKKVS

B_14_AAZ99996 IPISIPSRNW AAFRGWSFTR LKTKETPSLG SGFDPYFVYS GSIPYLDGTF YLNHTFKKVS

B_34_AAW33485 IPISIPSRNW AAFRGWSFTR LKTKETPSLG SGFDPYFVYS GSIPYLDGTF YLNHTFKKVS

B_79_BAW32492 IPISIPSRNW AAFRGWSFTR LKTKETPSLG SGFDPYFVYS GSIPYLDGTF YLNHTFKKVS

B_68_AET87230 IPISIPSRNW AAFRGWSFTR LKTKETPSLG SGFDPYFVYS GSIPYLDGTF YLNHTFKKVS

B_66_AET87148 IPISIPSRNW AAFRGWSFTR LKTKETPSLG SGFDPYFVYS GSIPYLDGTF YLNHTFKKVS

E_4_AAD03660 VPISIPSRNW AAFRGWSFTR LKTKETPSLG SGFDPYFVYS GSIPYLDGTF YLNHTFKKVS

B_16_AAW33444 IPISIPSRNW AAFRGWSFTR LKTKETPSLG SGFDPYFVYS GSIPYLDGTF YLNHTFKKVS

A_61_AEK79922 VPISIPSRNW AAFRGWSFTR LKTKETPSLG SGFDPYFVYS GTIPYLDGTF YLNHTFKKVS

A_12_BAG48789 VPISIPSRNW AAFRGWSFTR LKTKETPSLG SGFDPYFVYS GTIPYLDGTF YLNHTFKKVS

A_18_AAZ99994 VPISIPSRNW AAFRGWSFTR LKTKETPSLG SGFDPYFVYS GTIPYLDGTF YLNHTFKKVS

A_31_CAO78638 VPISIPSRNW AAFRGWSFTR LKTKETPSLG SGFDPYFVYS GTIPYLDGTF YLNHTFKKVS

G_52_ABK35044 VPISIPSRNW AAFRGWSFTR LKTKETPSLG SGFDPYFVYS GSIPYLDGTF YLNHTFKKVS

F_41_ACH90432 VPISIPSRNW AAFRGWSFTR LKTKETPSLG SGFDPYFTYS GSVPYLDGTF YLNHTFKKVS

F_40_AMQ95234 VPISIPSRNW AAFRGWSFTR LKTKETPSLG SGFDPYFTYS GSVPYLDGTF YLNHTFKKVS

....|....| ....|....| ....|....| ....|....| ....|....| ....|....|

785 795 805 815 825 835

HexN1 IMFDSSVSWP GNDRLLTPNE FEIKRSVDGE GYNVAQCNMT KDWFLVQMLS HYNIGYQGFH

B_3_AAW33168 IMFDSSVSWP GNDRLLSPNE FEIKRTVDGE GYNVAQCNMT KDWFLVQMLA NYNIGYQGFY

C_5_AAW65514 ITFDSSVSWP GNDRLLTPNE FEIKRSVDGE GYNVAQCNMT KDWFLVQMLA NYNIGYQGFY

B_7_AAD03663 IMFDSSVSWP GNDRLLSPNE FEIKRTVDGE GYNVAQCNMT KDWFLVQMLA NYNIGYQGFY

C_2_AAO24095 ITFDSSVSWP GNDRLLTPNE FEIKRSVDGE GYNVAQCNMT KDWFLVQMLA NYNIGYQGFY

C_1_AAQ10553 ITFDSSVSWP GNDRLLTPNE FEIKRSVDGE GYNVAQCNMT KDWFLVQMLA NYNIGYQGFY

C_57_ADM46153 ITFDSSVSWP GNDRLLTPNE FEIKRSVDGE GYNVAQCNMT KDWFLVQMLA NYNIGYQGFY

C_6_ADV03661 ITFDSSVSWP GNDRLLTPNE FEIKRSVDGE GYNVAQCNMT KDWFLVQMLA NYNIGYQGFY

D_37_ABA00016 IMFDSSVSWP GNDRLLTPNE FEIKRSVDGE GYNVAQCNMT KDWFLVQMLS HYNIGYQGFH

D_13_ABA00000 IMFDSSVSWP GNDRLLTPNE FEIKRSVDGE GYNVAQCNMT KDWFLVQMLS HYNIGYQGFH

D_70_AKI33526 IMFDSSVSWP GNDRLLTPNE FEIKRSVDGE GYNVAQCNMT KDWFLVQMLS HYNIGYQGFY

D_63_AEV92966 IMFDSSVSWP GNDRLLTPNE FEIKRSVDGE GYNVAQCNMT KDWFLVQMLS HYNIGYQGFH

D_72_AGT78001 IMFDSSVSWP GNDRLLTPNE FEIKRSVDGE GYNVAQCNMT KDWFLVQMLS HYNIGYQGFH

D_23_ABA00005 LMFDSSVSWP GNDRLLTPNE FEIKRSVDGE GYNVAQCNMT KDWFLVQMLS HYNIGYQGFH

D_24_ABA00006 IMFDSSVSWP GNDRLLTPNE FEIKRSVDGE GYNVAQCNMT KDWFLVQMLS HYNIGYQGFH

D_39_ABA00018 IMFDSSVSWP GNDRLLTPNE FEIKRSVDGE GYNVAQCNMT KDWFLVQMLS HYNIGYQGFH

D_43_ABA00020 IMFDSSVSWP GNDRLLTPNE FEIKRSVDGE GYNVAQCNMT KDWFLVQMLS HYNIGYQGFH

D_58_ADW95419 IMFDSSVSWP GNDRLLTPNE FEIKRSVDGE GYNVAQCNMT KDWFLVQMLS HYNIGYQGFH

D_33_ABA00014 IMFDSSVSWP GNDRLLTPNE FEIKRSVDGE GYNVAQCNMT KDWFLVQMLS HYNIGYQGFH

D_38_ABA00017 IMFDSSVSWP GNDRLLTPNE FEIKRSVDGE GYNVAQCNMT KDWFLVQMLS HYNIGYQGFH

D_36_ACY04472 IMFDSSVSWP GNDRLLTPNE FEIKRSVDGE GYNVAQCNMT KDWFLVQMLS HYNIGYQGFY

D_49_ABA00025 IMFDSSVSWP GNDRLLTPNE FEIKRSVDGE GYNVAQCNMT KDWFLVQMLS HYNIGYQGFH

D_8_AAZ99998 IMFDSSVSWP GNDRLLTPNE FEIKRSVDGE GYNVAQCNMT KDWFLVQMLS HYNIGYQGFH

D_46_ABA00023 IMFDSSVSWP GNDRLLTPNE FEIKRSVDGE GYNVAQCNMT KDWFLVQMLS HYNIGYQGFH

D_47_ABA00024 IMFDSSVSWP GNDRLLTPNE FEIKRSVDGE GYNVAQCNMT KDWFLVQMLS HYNIGYQGFH

D_44_ABA00021 IMFDSSVSWP GNDRLLTPNE FEIKRSVDGE GYNVAQCNMT KDWFLVQMLS HYNIGYQGFH

D_48_ABO61301 IMFDSSVSWP GNDRLLTPNE FEIKRSVDGE GYNVAQCNMT KDWFLVQMLS HYNIGYQGFH

D_22_ABA00004 IMFDSSVSWP GNDRLLTPNE FEIKRSVDGE GYNVAQCNMT KDWFLVQMLS HYNIGYQGFH

D_53_BAJ46350 IMFDSSVSWP GNDRLLTPNE FEIKRSVDGE GYNVAQCNMT KDWFLVQMLS HYNIGYQGFH

D_45_ABA00022 IMFDSSVSWP GNDRLLTPNE FEIKRSVDGE GYNVAQCNMT KDWFLVQMLS HYNIGYQGFH

D_26_ABA00008 IMFDSSVSWP GNDRLLTPNE FEIKRSVDGE GYNVAQCNMT KDWFLVQMLS HYNIGYQGFH

D_32_ABA00013 IMFDSSVSWP GNDRLLTPNE FEIKRSVDGE GYNVAQCNMT KDWFLVQMLS HYNIGYQGFH

D_73_APD28385 IMFDSSVSWP GNDRLLTPNE FEIKRSVDGE GYNVAQCNMT KDWFLVQMLS HYNIGYQGFH

D_67_BAL63187 IMFDSSVSWP GNDRLLTPNE FEIKRSVDGE GYNVAQCNMT KDWFLVQMLS HYNIGYQGFH

D_9_BAE66671 IMFDSSVSWP GNDRLLTPNE FEIKRSVDGE GYNVAQCNMT KDWFLVQMLS HYNIGYQGFH

D_54_YP_0030386 IMFDSSVSWP GNDRLLTPNE FEIKRSVDGE GYNVAQCNMT KDWFLIQMLS HYNIGYQGFH

D_17_ADY18429 IMFDSSVSWP GNDRLLTPNE FEIKRSVDGE GYNVAQCNMT KDWFLVQMLS HYNIGYQGFH

D_10_AAZ99999 IMFDSSVSWP GNDRLLTPNE FEIKRSVDGE GYNVAQCNMT KDWFLVQMLS HYNIGYQGFH

D_65_BAL41721 IMFDSSVSWP GNDRLLTPNE FEIKRSVDGE GYNVAQCNMT KDWFLVQMLS HYNIGYQGFH

D_62_AEL78842 IMFDSSVSWP GNDRLLTPNE FEIKRSVDGE GYNVAQCNMT KDWFLVQMLS HYNIGYQGFH

D_25_ABA00007 IMFDSSVSWP GNDRLLTPNE FEIKRSVDGE GYNVAQCNMT KDWFLVQMLS HYNIGYQGFH

D_59_AEI91289 IMFDSSVSWP GNDRLLTPNE FEIKRSVDGE GYNVAQCNMT KDWFLVQMLS HYNIGYQGFH

D_19_ABA00002 IMFDSSVSWP GNDRLLTPNE FEIKRSVDGE GYNVAQCNMT KDWFLVQMLS HYNIGYQGFH

D_64_ABN10535 IMFDSSVSWP GNDRLLTPNE FEIKRSVDGE GYNVAQCNIT KDWFLVQMLS HYNIGYQGFH

D_27_ABA00009 IMFDSSVSWP GNDRLLTPNE FEIKRSVDGE GYNVAQCNMT KDWFLVQMLS HYNIGYQGFH

D_28_ACQ91158 IMFDSSVSWP GNDRLLTPNE FEIKRSVDGE GYNVAQCNMT KDWFLVQMLS HYNIGYQGFH

D_20_ABA00003 IMFDSSVSWP GNDRLLTPNE FEIKRSVDGE GYNVAQCNMT KDWFLVQMLS HYNIGYQGFH

D_60_AEK87026 IMFDSSVSWP GNDRLLTPNE FEIKRSVDGE GYNVAQCNMT KDWFLVQMLS HYNIGYQGFH

D_71_AGT76762 IMFDSSVSWP GNDRLLTPNE FEIKRSVDGE GYNVAQCNMT KDWFLVQMLS HYNIGYQGFH

D_42_ABA00019 IMFDSSVSWP GNDRLLTPNE FEIKRSVDGE GYNVAQCNMT KDWFLVQMLS HYNIGYQGFH

D_51_ABA00026 IMFDSSVSWP GNDRLLTPNE FEIKRSVDGE GFNVAQCNMT KDWFLVQMLS HYNIGYQGFH

D_30_ABA00012 IMFDSSVSWP GNDRLLTPNE FEIKRSVDGE GYNVAQCNMT KDWFLVQMLS HYNIGYQGFH

D_29_BAJ22326 IMFDSSVSWP GNDRLLTPNE FEIKRSVDGE GYNVAQCNMT KDWFLVQMLS HYNIGYQGFY

D_56_ADM66117 IMFDSSVSWP GNDRLLTPNE FEIKRSVDGE GYNVAQCNMT KDWFLVQMLS HYNIGYQGFH

D_69_AFK92217 IMFDSSVSWP GNDRLLTPNE FEIKRSVDGE GYNVAQCNMT KDWFLVQMLS HYNIGYQGFH

D_15_BAJ22290 IMFDSSVSWP GNDRLLTPNE FEIKRSVDGE GYNVAQCNMT KDWFLVQMLS HYNIGYQGFH

B_35_AAP92351 IMFDSSVSWP GNDRLLSPNE FEIKRTVDGE GYNVAQCNMT KDWFLVQMLA NYNIGYQGFY

B_11_ACZ06785 IMFDSSVSWP GNDRLLSPNE FEIKRTVDGE GYNVAQCNMT KDWFLVQMLA NYNIGYQGFY

B_55_AIS92536 IMFDSSVSWP GNDRLLSPNE FEIKRTVDGE GYNVAQCNMT KDWFLVQMLA NYNIGYQGFY

B_21_AAG21823 IMFDSSVSWP GNDRLLSPNE FEIKRTVDGE GYNVAQCNMT KDWFLVQMLA NYNIGYQGFY

B_50_ABA00027 IMFDSSVSWP GNDRLLSPNE FEIKRTVDGE GYNVAQCNMT KDWFLVQMLA NYNIGYQGFY

B_14_AAZ99996 IMFDSSVSWP GNDRLLSPNE FEIKRTVDGE GYNVAQCNMT KDWFLVQMLA NYNIGYQGFY

B_34_AAW33485 IMFDSSVSWP GNDRLLSPNE FEIKRTVDGE GYNVAQCNMT KDWFLVQMLA NYNIGYQGFY

B_79_BAW32492 IMFDSSVSWP GNDRLLSPNE FEIKRTVDGE GYNVAQCNMT KDWFLVQMLA NYNIGYQGFY

B_68_AET87230 IMFDSSVSWP GNDRLLSPNE FEIKRTVDGE GYNVAQCNMT KDWFLVQMLA NYNIGYQGFY

B_66_AET87148 IMFDSSVSWP GNDRLLSPNE FEIKRTVDGE GYNVAQCNMT KDWFLVQMLA NYNIGYQGFY

E_4_AAD03660 ITFDSSVSWP GNDRLLTPNE FEIKRTVDGE GYNVAQCNMT KDWFLVQMLA HYNIGYQGFY

B_16_AAW33444 IMFDSSVSWP GNDRLLSPNE FEIKRTVDGE GYNVAQCNMT KDWFLVQMLA NYNIGYQGFY

A_61_AEK79922 IMFDSSVSWP GNDRLLTPNE FEIKRSVDGE GYNVAQCNMT KDWFLIQMLS HYNIGYQGFY

A_12_BAG48789 IMFDSSVSWP GNDRLLTPNE FEIKRSVDGE GYNVAQCNMT KDWFLIQMLS HYNIGYQGFY

A_18_AAZ99994 IMFDSSVSWP GNDRLLTPNE FEIKRSVDGE GYNVAQCNMT KDWFLIQMLS HYNIGYQGFY

A_31_CAO78638 IMFDSSVSWP GNDRLLTPNE FEIKRSVDGE GYNVAQCNMT KDWFLVQMLS HYNIGYQGFY

G_52_ABK35044 IMFDSSVSWP GNDRLLTPNE FEIKRSVDGE GYNVAQSNMT KDWFLIQMLS HYNIGYQGFY

F_41_ACH90432 IMFDSSVSWP GNDRLLTPNE FEIKRTVDGE GYNVAQCNMT KDWFLIQMLS HYNIGYQGFY

F_40_AMQ95234 VMFDSSVSWP GNDRLLTPNE FEIKRTVDGE GYNVAQCNMT KDWFLIQMLS HYNIGYQGFH

....|....| ....|....| ....|....| ....|....| ....|....| ....|....|

845 855 865 875 885 895

HexN1 VPEGYKDRMY SFFRNFQPMS RQVVDEINYK D-YKAVTLPF QHNNSGFTGY LAPTMRQGQP

B_3_AAW33168 IPEGYKDRMY SFFRNFQPMS RQVVDEVNYT D-YKAVTLPY QHNNSGFVGY LAPTMRQGEP

C_5_AAW65514 IPESYKDRMY SFFRNFQPMS RQVVDDTKYK D-YQQVGILH QHNNSGFVGY LAPTMREGQA

B_7_AAD03663 IPEGYKDRMY SFFRNFQPMS RQVVDEVNYT D-YKAVTLPY QHNNSGFVGY LAPTMRQGEP

C_2_AAO24095 IPESYKDRMY SFFRNFQPMS RQVVDDTKYK E-YQQVGILH QHNNSGFVGY LAPTMREGQA

C_1_AAQ10553 IPESYKDRMY SFFRNFQPMS RQVVDDTKYK D-YQQVGILH QHNNSGFVGY LAPTMREGQA

C_57_ADM46153 IPESYKDRMY SFFRNFQPMS RQVVDDTKYK D-YQQVGILH QHNNSGFVGY LAPTMREGQA

C_6_ADV03661 IPESYKDRMY SFFRNFQPMS RQVVDDTKYK D-YQQVGIIH QHNNSGFVGY LAPTMREGQA

D_37_ABA00016 VPEGYKDRMY SFFRNFQPMS RQVVDEINYK D-YKAVTLPF QHNNSGFTGY LAPTMRQGQP

D_13_ABA00000 VPEGYKDRMY SFFRNFQPMS RQVVDEINYK D-YKAVTLPF QHNNSGFTGY LAPTMRQGQP

D_70_AKI33526 VPEGYKDRMY SFFRNFQPMS RQVVDEINYK D-YKAVTLPF QHNNSGFTGY LAPTMRQGQP

D_63_AEV92966 VPEGYKDRMY SFFRNFQPMS RQVVDEINYK D-YKAVTLPF QHNNSGFVGY LAPTMRQGQP

D_72_AGT78001 VPEGYKDRMY SFFRNFQPMS RQVVDEINYK D-YKAVTLPF QHNNSGFTGY LAPTMRQGQP

D_23_ABA00005 VPEGYKDRMY SFFRNFQPMS RQVVDEINYK D-YKAVTLPF QHNNSGFTGY LAPTMRQGQP

D_24_ABA00006 VPEGYKDRMY SFFRNFQPMS RQVVDEINYK D-YKAVTLPF QHNNSGFTGY LAPTMRQGQP

D_39_ABA00018 VPEGYKDRMY SFFRNFQPMS RQVVDEINYK D-YKAVTLPF QHNNSGFTGY LAPTMRQGQP

D_43_ABA00020 VPEGYKDRMY SFFRNFQPMS RQVVDEINYK D-YKAVTLPF QHNNSGFTGY LAPTMRQGQP

D_58_ADW95419 VPEGYKDRMY SFFRNFQPMS RQVVDEINYK D-YKAVTLPF QHNNSGFTGY LAPTMRQGQP

D_33_ABA00014 VPEGYKDRMY SFFRNFQPMS RQVVDEINYK D-YKAVTLPF QHNNSGFTGY LAPTMRQGQP

D_38_ABA00017 VPEGYKDRMY SFFRNFQPMS RQVVDEINYK D-YKAVTLPF QHNNSGFTGY LAPTMRQGQP

D_36_ACY04472 VPEGYKDRMY SFFRNFQPMS RQVVDEINYK D-YKAVTLPF QHNNSGFTGY LAPTMRQGQP

D_49_ABA00025 VPEGYKDRMY SFFRNFQPMS RQVVDEINYK D-YKAVTLPF QHNNSGFTGY LAPTMRQGQP

D_8_AAZ99998 VPEGYKDRMY SFFRNFQPMS RQVVDEINYK D-YKAVALPF QHNNSGFTGY LAPTLRQGQP

D_46_ABA00023 VPEGYKDRMY SFFRNFQPMS RQVVDEINYK D-YKAVTLPF QHNNSGFTGY LAPTMRQGQP

D_47_ABA00024 VPEGYKDRMY SFFRNFQPMS RQVVDEINYK D-YKAVTLPF QHNNSGFTGY LAPTMRQGQP

D_44_ABA00021 VPEGYKDRMY SFFRNFQPMS RQVVDEINYK D-YKAVTLPF QHNNSGFTGY LAPTMRQGQP

D_48_ABO61301 VPEGYKDRMY SFFRNFQPMS RQVVDEINYK D-YKAVTLPF QHNNSGFTGY LAPTMRQGQP

D_22_ABA00004 VPEGYKDRMY SFFRNFQPMS RQVVDEINYK D-YKAVTLPF QHNNSGFTGY LAPTMRQGQP

D_53_BAJ46350 VPEGYKDRMY SFFRNFQPMS RQVVDEINYK D-YKAVTLPF QHNNSGFTGY LAPTMRQGQP

D_45_ABA00022 VPEGYKDRMY SFFRNFQPMS RQVVDEINYK D-YKAVTLPF QHNNSGFTGY LAPTMRQGQP

D_26_ABA00008 VPEGYKDRMY SFFRNFQPMS RQVVDEINYK D-YKAVTLPF QHNNSGFTGY LAPTMRQGQP

D_32_ABA00013 VPEGYKDRMY SFFRNFQPMS RQVVDEINYK D-YKAVTLPF QHNNSGFTGY LAPTMRQGQP

D_73_APD28385 VPEGYKDRMY SFFRNFQPMS RQVVDEINYK D-YKAVTLPF QHNNSGFTGY LAPTMRQGQP

D_67_BAL63187 VPEGYKDRMY SFFRNFQPMS RQVVDEINYK D-YKAVTLPF QHNNSGFTGY LAPTMRQGQP

D_9_BAE66671 VPEGYKDRMY SFFRNFQPMS RQVVDEINYK D-YKAVTLPF QHNNSGFTGY LAPTMRQGQP

D_54_YP_0030386 VPEGYKDRMY SFFRNFQPMS RQVVDEINYK D-YKAVALPF QHNNSGFTGY LAPTMRQGQP

D_17_ADY18429 VPEGYKDRMY SFFRNFQPMS RQVVDEINYK D-YKAVTLPF QHNNSGFTGY LAPTMRQGQP

D_10_AAZ99999 VPEGYKDRMY SFFRNFQPMS RQVVDEINYK D-YKAVTLPF QHNNSGFTGY LAPTMRQGQP

D_65_BAL41721 VPEGYKDRMY SFFRNFQPMS RQVVDEINYK D-YKAVTLPF QHNNSGFTGY LAPTMRQGQP

D_62_AEL78842 VPEGYKDRMY SFFRNFQPMS RQVVDEINYK D-YKAVTLPF QHNNSGFVGY LAPTMRQGQP

D_25_ABA00007 VPEGYKDRMY SFFRNFQPMS RQVVDEINYK D-YKAVTLPF QHNNSGFTGY LAPTMRQGQP

D_59_AEI91289 VPEGYKDRMY SFFRNFQPMS RQVVDEINYK D-YKAVTLPF QHNNSGFTGY LAPTMRQGQP

D_19_ABA00002 VPEGYKDRMY SFFRNFQPMS RQVVDEINYK D-YKAVTLPF QHNNSGFTGY LAPTMRQGQP

D_64_ABN10535 VPEGYKDRMY SFFRNFQPMS RQVVDEINYK D-YKAVTLPF QHNNSGFTGY LAPTMRQGQP

D_27_ABA00009 VPEGYKDRMY SFFRNFQPMS RQVVDEINYK D-YKAVTLPF QHNNSGFTGY LAPTMRQGQP

D_28_ACQ91158 VPEGYKDRMY SFFRNFQPMS RQVVDEINYK D-YKAVTLPF QHNNSGFTGY LAPTMRQGQP

D_20_ABA00003 VPEGYKDRMY SFFRNFQPMS RQVVDEINYK D-YKAVTLPF QHNNSGFTGY LAPTMRQGQP

D_60_AEK87026 VPEGYKDRMY SFFRNFQPMS RQVVDEINYK D-YKAVTLPF QHNNSGFTGY LAPTMRQGQP

D_71_AGT76762 VPEGYKDRMY SFFRNFQPMS RQVVDEINYK D-YKAVTLPF QHNNSGFVGY LAPTMRQGQP

D_42_ABA00019 VPEGYKDRMY SFFRNFQPMS RQVVDEINYK D-YKAVTLPF QHNNSGFTGY LAPTMRQGQP

D_51_ABA00026 VPEGYKDRMY SFFRNFQPMS RQVVDEINYK D-YKAVTLPF QHNNSGFTGY LAPTMRQGQP

D_30_ABA00012 VPEGYKDRMY SFFRNFQPMS RQVVDEINYK D-YKAVTLPF QHNNSGFVGY LAPTMRQGQP

D_29_BAJ22326 VPEGYKDRMY SFFRNFQPMS RQVVDEINYK D-YKAVTLPF QHNNSGFTGY LAPTMRQGQP

D_56_ADM66117 VPEGYKDRMY SFFRNFQPMS RQVVDEINYK D-YKAVTLPF QHNNSGFTGY LAPTMRQGQP

D_69_AFK92217 VPEGYKDRMY SFFRNFQPMS RQVVDEINYK D-YKAVTLPF QHNNSGFTGY LAPTMRQGQP

D_15_BAJ22290 VPEGYKDRMY SFFRNFQPMS RQVVDEINYK D-YKAVTLPF QHNNSGFTGY LAPTMRQGQP

B_35_AAP92351 IPEGYKDRMY SFFRNFQPMS RQVVDEVNYK D-FKAVAIPY QHNNSGFVGY MAPTMRQGQP

B_11_ACZ06785 IPEGYKDRMY SFFRNFQPMS RQVVDEVNYK D-FKAVAIPY QHNNSGFVGY MAPTMRQGQP

B_55_AIS92536 IPEGYKDRMY SFFRNFQPMS RQVVDEVNYK D-FKAVAIPY QHNNSGFVGY MAPTMRQGQP

B_21_AAG21823 VPEGYKDRMY SFFRNFQPMS RQVVDEINYK D-YKAVAVPY QHNNSGFVGY MAPTMRQGQA

B_50_ABA00027 VPEGYKDRMY SFFRNFQPMS RQVVDEINYK D-YKAVAVPY QHNNSGFVGY MAPTMRQGQA

B_14_AAZ99996 IPEGYKDRMY SFFRNFQPMS RQVVDEVNYK D-FKAVAIPY QHNNSGFVGY MAPTMRQGQP

B_34_AAW33485 IPEGYKDRMY SFFRNFQPMS RQVVDEVNYK D-FKAVAIPY QHNNSGFVGY MAPTMRQGQP

B_79_BAW32492 IPEGYKDRMY SFFRNFQPMS RQVVDEVNYK D-FKAVAIPY QHNNSGFVGY MAPTMRQGQP

B_68_AET87230 IPEGYKDRMY SFFRNFQPMS RQVVDEVNYT D-YKAVTLPY QHNNSGFVGY LAPTMRQGEP

B_66_AET87148 IPEGYKDRMY SFFRNFQPMS RQVVDEVNYT D-YKAVTLPY QHNNSGFVGY LAPTMRQGEP

E_4_AAD03660 VPEGYKDRMY SFFRNFQPMS RQVVDEVNYK D-YQAVTLPY QHNNSGFVGY LAPTMRRGQP

B_16_AAW33444 IPEGYKDRMY SFFRNFQPMS RQVVDEVNYT D-YKAVTLPY QHNNSGFVGY LAPTMRQGEP

A_61_AEK79922 VPESYKDRMY SFFRNFQPMS RQVVDTTEYK E-YKKVTVEF QHNNSGFVGY LGPTMREGQA

A_12_BAG48789 IPESYKDRMY SFFRNFQPMS RQVVDTTEYK N-YKKVTVEF QHNNSGFVGY LGPTMREGQA

A_18_AAZ99994 VPEGYKDRMY SFFRNFQPMS RQVVDTTEYK D-YKQVTVKF QHNNSGFVGY LGPTMREGQA

A_31_CAO78638 VPESYKDRMY SFFRNFQPMS RQVVDTTEYK E-YKKVTVEF QHNNSGFVGY LGPTMREGQA

G_52_ABK35044 VPENYKDRMY SFFRNFQPMS RQVVDTVAYK DYYQDVKLPY QHNNSGFVGY MGPTMREGQA

F_41_ACH90432 VPESYKDRMY SFFRNFQPMS RQVVNTTTYK E-YQNVTLPF QHNNSGFVGY MGPTMREGQA

F_40_AMQ95234 VPESYKDRMY SFFRNFQPMS RQVVDTTTYT E-YQNVTLPF QHNNSGFVGY MGPAIREGQA

....|....| ....|....| ....|....| ....|....| ....|....| ....|....|

905 915 925 935 945 955

HexN1 YPANFPYPLI GSTAVPSVTQ KKFLCDRVMW RIPFSSNFMS MGALTDLGQN MLYANSAHAL

B_3_AAW33168 YPANYPYPLI GTTAVKSVTQ KKFLCDRTMW RIPFSSNFMS MGALTDLGQN MLYANSAHAL

C_5_AAW65514 YPANFPYPLI GKTAVDSITQ KKFLCDRTLW RIPFSSNFMS MGALTDLGQN LLYANSAHAL

B_7_AAD03663 YPANYPYPLI GTTAVKSVTQ KKFLCDRTMW RIPFSSNFMS MGALTDLGQN MLYANSAHAL

C_2_AAO24095 YPANVPYPLI GKTAVDSITQ KKFLCDRTLW RIPFSSNFMS MGALTDLGQN LLYANSAHAL

C_1_AAQ10553 YPANFPYPLI GKTAVDSITQ KKFLCDRTLW RIPFSSNFMS MGALTDLGQN LLYANSAHAL

C_57_ADM46153 YPANFPYPLI GKTAVDSITQ KKFLCDRTLW RIPFSSNFMS MGALTDLGQN LLYANSAHAL

C_6_ADV03661 YPANVPYPLI GKTAVDSITQ KKFLCDRTLW RIPFSSNFMS MGALTDLGQN LLYANSAHAL

D_37_ABA00016 YPANFPYPLI GSTAVPSVTQ KKFLCDRVMW RIPFSSNFMS MGALTDLGQN MLYANSAHAL

D_13_ABA00000 YPANFPYPLI GSTAVPSVTQ KKFLCDRVMW RIPFSSNFMS MGALTDLGQN MLYANSAHAL

D_70_AKI33526 YPANFPYPLI GQTAVPSVTQ KKFLCDRVMW RIPFSSNFMS MGALTDLGQN MLYANSAHAL

D_63_AEV92966 YPANFPYPLI GQTAVPSVTQ KKFLCDRVMW RIPFSSNFMS MGALTDLGQN MLYANSAHAL

D_72_AGT78001 YPANFPYPLI GQTAVPSVTQ KKFLCDRVMW RIPFSSNFMS MGALTDLGQN MLYANSAHAL

D_23_ABA00005 YPANFPYPLI GSTAVPSVTQ KKFLCDRVMW RIPFSSNFMS MGALTDLGQN MLYANSAHAL

D_24_ABA00006 YPANFPYPLI GSTAVPSVTQ KKFLCDRVMW RIPFSSNFMS MGALTDLGQN MLYANSAHAL

D_39_ABA00018 YPANFPYPLI GSTAVPSVTQ KKFLCDRVMW RIPFSSNFMS MGALTDLGQN MLYANSAHAL

D_43_ABA00020 YPANFPYPLI GQTAVPSVTQ KKFLCDRVMW RIPFSSNFMS MGALTDLGQN MLYANSAHAL

D_58_ADW95419 YPANFPYPLI GSTAVPSVTQ KKFLCDRVMW RIPFSSNFMS MGALTDLGQN MLYANSAHAL

D_33_ABA00014 YPANFPYPLI GSTAVPSVTQ KKFLCDRVMW RIPFSSNFMS MGALTDLGQN MLYANSAHAL

D_38_ABA00017 YPANFPYPLI GQTAVPSVTQ KKFLCDRVMW RIPFSSNFMS MGALTDLGQN MLYANSAHAL

D_36_ACY04472 YPANFPYPLI GQTAVPSVTQ KKFLCDRVMW RIPFSSNFMS MGALTDLGQN MLYANSAHAL

D_49_ABA00025 YPANFPYPLI GSTAVPSVTQ KKFLCDRVMW RIPFSSNFMS MGALTDLGQN MLYANSAHAL

D_8_AAZ99998 YPANFPYPLI GQTAVPSVTQ KKFLCDRVMW RIPFSSNFMS MGALTDLGQN MLYANSAHAL

D_46_ABA00023 YPANFPYPLI GSTAVPSVTQ KKFLCDRVMW RIPFSSNFMS MGALTDLGQN MLYANSAHAL

D_47_ABA00024 YPANFPYPLI GSAAVPSVTQ KKFLCDRVMW RIPFSSNFMS MGALTDLGQN MLYANSAHAL

D_44_ABA00021 YPANFPYPLI GQTAVPSVTQ KKFLCDRVMW RIPFSSNFMS MGALTDLGQN MLYANSAHAL

D_48_ABO61301 YPANFPYPLI GQTAVPSVTQ KKFLCDRVMW RIPFSSNFMS MGALTDLGQN MLYANSAHAL

D_22_ABA00004 YPANFPYPLI GQTAVPSVTQ KKFLCDRVMW RIPFSSNFMS MGALTDLGQN MLYANSAHAL

D_53_BAJ46350 YPANFPYPLI GSTAVPSVTQ KKFLCDRVMW RIPFSSNFMS MGALTDLGQN MLYANSAHAL

D_45_ABA00022 YPANFPYPLI GQTAVPSVTQ KKFLCDRVMW RIPFSSNFMS MGALTDLGQN MLYANSAHAL

D_26_ABA00008 YPANFPYPLI GQTAVPSVTQ KKFLCDRVMW RIPFSSNFMS MGALTDLGQN MLYANSAHAL

D_32_ABA00013 YPANFPYPLI GSTAVPSVTQ KKFLCDRVMW RIPFSSNFMS MGALTDLGQN MLYANSAHAL

D_73_APD28385 YPANFPYPLI GSTAVPSVTQ KKFLCDRVMW RIPFSSNFMS MGALTDLGQN MLYANSAHAL

D_67_BAL63187 YPANFPYPLI GQTAVPSVTQ KKFLCDRVMW RIPFSSNFMS MGALTDLGQN MLYANSAHAL

D_9_BAE66671 YPANFPYPLI GQTAVPSVTQ KKFLCDRVMW RIPFSSNFMS MGALTDLGQN MLYANSAHAL

D_54_YP_0030386 YPANFPYPLI GETAVPSVTQ KKFLCDRVMW RIPFSSNFMS MGALTDLGQN MLYANSAHAL

D_17_ADY18429 YPANFPYPLI GQTAVPSVTQ KKFLCDRVMW RIPFSSNFMS MGALTDLGQN MLYANSAHAL

D_10_AAZ99999 YPANFPYPLI GQTAVPSVTQ KKFLCDRVMW RIPFSSNFMS MGALTDLGQN MLYANSAHAL

D_65_BAL41721 YPANFPYPLI GSTAVPSVTQ KKFLCDRVMW RIPFSSNFMS MGALTDLGQN MLYANSAHAL

D_62_AEL78842 YPANFPYPLI GSTAVPSVTQ KKFLCDRVMW RIPFSSNFMS MGALTDLGQN MLYANSAHAL

D_25_ABA00007 YPANFPYPLI GSTAVPSVTQ KKFLCDRVMW RIPFSSNFMS MGALTDLGQN MLYANSAHAL

D_59_AEI91289 YPANFPYPLI GQTAVPSVTQ KKFLCDRVMW RIPFSSNFMS MGALTDLGQN MLYANSAHAL

D_19_ABA00002 YPANFPYPLI GSTAVPSVTQ KKFLCDRVMW RIPFSSNFMS MGALTDLGQN MLYANSAHAL

D_64_ABN10535 YPANFPYPLI GSTAVPSVTQ KKFLCDRVMW RIPFSSNFMS MGALTDLGQN MLYANSAHAL

D_27_ABA00009 YPANFPYPLI GSTAVPSVTQ KKFLCDRVMW RIPFSSNFMS MGALTDLGQN MLYANSAHAL

D_28_ACQ91158 YPANFPYPLI GQTAVPSVTQ KKFLCDRVMW RIPFSSNFMS MGALTDLGQN MLYANSAHAL

D_20_ABA00003 YPANFPYPLI GQTAVPSVTQ KKFLCDRVMW RIPFSSNFMS MGALTDLGQN MLYANSAHAL

D_60_AEK87026 YPANFPYPLI GQTAVPSVTQ KKFLCDRVMW RIPFSSNFMS MGALTDLGQN MLYANSAHAL

D_71_AGT76762 YPANFPYPLI GQTAVPSVTQ KKFLCDRVMW RIPFSSNFMS MGALTDLGQN MLYANSAHAL

D_42_ABA00019 YPANFPYPLI GSTAVPSVTQ KKFLCDRVMW RIPFSSNFMS MGALTDLGQN MLYANSAHAL

D_51_ABA00026 YPANFPYPLI GSTAVPSVTQ KKFLCDRVMW RIPFSSNFMS MGALTDLGQN MLYANSAHAL

D_30_ABA00012 YPANFPYPLI GQTAVPSVTQ KKFLCDRVMW RIPFSSNFMS MGALTDLGQN MLYANSAHAL

D_29_BAJ22326 YPANFPYPLI GSTAVPSVTQ KKFLCDRVMW RIPFSSNFMS MGALTDLGQN MLYANSAHAL

D_56_ADM66117 YPANFPYPLI GQTAVPSVTQ KKFLCDRVMW RIPFSSNFMS MGALTDLGQN MLYANSAHAL

D_69_AFK92217 YPANFPYPLI GQTAVPSVTQ KKFLCDRVMW RIPFSSNFMS MGALTDLGQN MLYANSAHAL

D_15_BAJ22290 YPANFPYPLI GQTAVPSVTQ KKFLCDRVMW RIPFSSNFMS MGALTDLGQN MLYANSAHAL

B_35_AAP92351 YPANYPYPLI GTTAVNSVTQ KKFLCDRTMW RIPFSSNFMS MGALTDLGQN MLYANSAHAL

B_11_ACZ06785 YPANYPYPLI GTTAVNSVTQ KKFLCDRTMW RIPFSSNFMS MGALTDLGQN MLYANSAHAL

B_55_AIS92536 YPANYPYPLI GTTAVNSVTQ KKFLCDRTMW RIPFSSNFMS MGALTDLGQN MLYANSAHAL

B_21_AAG21823 YPANYPYPLI GTTAVTSVTQ KKFLCDRTMW RIPFSSNFMS MGALTDLGQN LLYANSAHAL

B_50_ABA00027 YPANYPYPLI GTTAVTSVTQ KKFLCDRTMW RIPFSSNFMS MGALTDLGQN LLYANSAHAL

B_14_AAZ99996 YPANYPYPLI GTTAVNSVTQ KKFLCDRTMW RIPFSSNFMS MGALTDLGQN MLYANSAHAL

B_34_AAW33485 YPANYPYPLI GTTAVNSVTQ KKFLCDRTMW RIPFSSNFMS MGALTDLGQN MLYANSAHAL

B_79_BAW32492 YPANYPYPLI GTTAVNSVTQ KKFLCDRTMW RIPFSSNFMS MGALTDLGQN MLYANSAHAL

B_68_AET87230 YPANYPYPLI GTTAVKSVTQ KKFLCDRTMW RIPFSSNFMS MGALTDLGQN LLYANSAHAL

B_66_AET87148 YPANYPYPLI GTTAVKSVTQ KKFLCDRTMW RIPFSSNFMS MGALTDLGQN LLYANSAHAL

E_4_AAD03660 YPANYPYPLI GKSAVTSVTQ KKFICDRVMW RIPFSSNFMS MGALTDLGQN MLYANSAHAL

B_16_AAW33444 YPANYPYPLI GTTAVKSVTQ KKFLCDRTMW RIPFSSNFMS MGALTDLGQN LLYANSAHAL

A_61_AEK79922 YPANYPYPLI GKTAVQSVTQ KKFLCDRVMW RIPFSSNFMS MGALTDLGQN MLYANSAHAL

A_12_BAG48789 YPANYPYPLI GKTAVESITQ KKFLCDRVMW RIPFSSNFMS MGALTDLGQN MLYANSAHAL

A_18_AAZ99994 YPANYPYPLI GKTAVESITQ KKFLCDRVMW RIPFSSNFMS MGALTDLGQN MLYANSAHAL

A_31_CAO78638 YPANYPYPLI GKTAVQSVTQ KKFLCDRVMW RIPFSSNFMS MGAVTDLGQN MLYANSAHAL

G_52_ABK35044 YPANYPYPLI GATAVPSLTQ KKFLCDRVMW RIPFSSNFMS MGSLTDLGQN MLYANSAHAL

F_41_ACH90432 YPANYPYPLI GQTAVPSLTQ KKFLCDRTMW RIPFSSNFMS MGALTDLGQN MLYANSAHAL

F_40_AMQ95234 YPANYPYPLI GQTAVPSLTQ KKFLCDRTMW RIPFSSNFMS MGALTDLGQN MLYANSAHAL

....|....| ....|....| ....|....| ....|....| ....|....| ...

965 975 985 995 1005

HexN1 DMTFEVDPMD EPTLLYLLFE VFDVVRVHQP HRGVIEAVYL RTPFSAGNAT T--

B_3_AAW33168 DMTFEVDPMD EPTLLYLLFE VFDVVRVHQP HRGVIEAVYL RTPFSAGNAT T--

C_5_AAW65514 DMTFEVDPMD EPTLLYVLFE VFDVVRVHQP HRGVIETVYL RTPFSAGNAT T--

B_7_AAD03663 DMTFEVDPMD EPTLLYLLFE VFDVVRVHQP HRGVIEAVYL RTPFSAGNAT T--

C_2_AAO24095 DMTFEVDPMD EPTLLYVLFE VFDVVRVHQP HRGVIETVYL RTPFSAGNAT T--

C_1_AAQ10553 DMTFEVDPMD EPTLLYVLFE VFDVVRVHQP HRGVIETVYL RTPFSAGNAT T--

C_57_ADM46153 DMTFEVDPMD EPTLLYVLFE VFDVVRVHQP HRGVIETVYL RTPFSAGNAT T--

C_6_ADV03661 DMTFEVDPMD EPTLLYVLFE VFDVVRVHQP HRGVIETVYL RTPFSAGNAT T--

D_37_ABA00016 DMTFEVDPMD EPTLLYLLFE VFDVVRVHQP HRGVIEAVYL RTPFSAGNAT T--

D_13_ABA00000 DMTFEVDPMD EPTLLYLLFE VFDVVRVHQP HRGVIEAVYL RTPFSAGNAT T--

D_70_AKI33526 DMTFEVDPMD EPTLLYLLFE VFDVVRVHQP HRGVIEAVYL RTPFSAGNAT T--

D_63_AEV92966 DMTFEVDPMD EPTLLYLLFE VFDVVRVHQP HRGVIEAVYL RTPFSAGNAT T--

D_72_AGT78001 DMTFEVDPMD EPTLLYLLFE VFDVVRVHQP HRGVIEAVYL RTPFSAGNAT T--

D_23_ABA00005 DMTFEVDPMD EPTLLYLLFE VFDVVRVHQP HRGVIEAVYL RTPFSAGNAT T--

D_24_ABA00006 DMTFEVDPMD EPTLLYLLFE VFDVVRVHQP HRGVIEAVYL RTPFSAGNAT T--

D_39_ABA00018 DMTFEVDPMD EPTLLYLLFE VFDVVRVHQP HRGVIEAVYL RTPFSAGNAT T--

D_43_ABA00020 DMTFEVDPMD EPTLLYLLFE VFDVVRVHQP HRGVIEAVYL RTPFSAGNAT T--

D_58_ADW95419 DMTFEVDPMD EPTLLYLLFE VFDVVRVHQP HRGVIEAVYL RTPFSAGNAT T--

D_33_ABA00014 DMTFEVDPMD EPTLLYLLFE VFDVVRVHQP HRGVIEAVYL RTPFSAGNAT T--

D_38_ABA00017 DMTFEVDPMD EPTLLYLLFE VFDVVRVHQP HRGVIEAVYL RTPFSAGNAT T--

D_36_ACY04472 DMTFEVDPMD EPTLLYLLFE VFDVVRVHQP HRGVIEAVYL RTPFSAGNAT T--

D_49_ABA00025 DMTFEVDPMD EPTLLYLLFE VFDVVRVHQP HRGVIEAVYL RTPFSAGNAT T--

D_8_AAZ99998 DMTFEVDPMD EPTLLYLLFE VFDVVRVHQP HRGVIEAVYL RTPFSAGNAT T--

D_46_ABA00023 DMTFEVDPMD EPTLLYLLFE VFDVVRVHQP HRGVIEAVYL RTPFSAGNAT T--

D_47_ABA00024 DMTFEVDPMD EPTLLYLLFE VFDVVRVHQP HRGVIEAVYL RTPFSAGNAT T--

D_44_ABA00021 DMTFEVDPMD EPTLLYLLFE VFDVVRVHQP HRGVIEAVYL RTPFSAGNAT T--

D_48_ABO61301 DMTFEVDPMD EPTLLYLLFE VFDVVRVHQP HRGVIEAVYL RTPFSAGNAT T--

D_22_ABA00004 DMTFEVDPMD EPTLLYLLFE VFDVVRVHQP HRGVIEAVYL RTPFSAGNAT T--

D_53_BAJ46350 DMTFEVDPMD EPTLLYLLFE VFDVVRVHQP HRGVIEAVYL RTPFSAGNAT T--

D_45_ABA00022 DMTFEVDPMD EPTLLYLLFE VFDVVRVHQP HRGVIEAVYL RTPFSAGNAT T--

D_26_ABA00008 DMTFEVDPMD EPTLLYLLFE VFDVVRVHQP HRGVIEAVYL RTPFSAGNAT T--

D_32_ABA00013 DMTFEVDPMD EPTLLYLLFE VFDVVRVHQP HRGVIEAVYL RTPFSAGNAT TKA

D_73_APD28385 DMTFEVDPMD EPTLLYLLFE VFDVVRVHQP HRGVIEAVYL RTPFSAGNAT T--

D_67_BAL63187 DMTFEVDPMD EPTLLYLLFE VFDVVRVHQP HRGVIEAVYL RTPFSAGNAT T--

D_9_BAE66671 DMTFEVDPMD EPTLLYLLFE VFDVVRVHQP HRGVIEAVYL RTPFSAGNAT T--

D_54_YP_0030386 DMTFEVDPMD EPTLLYLLFE VFDVVRVHQP HRGVIEAVYL RTPFSAGNAT T--

D_17_ADY18429 DMTFEVDPMD EPTLLYLLFE VFDVVRVHQP HRGVIEAVYL RTPFSAGNAT T--

D_10_AAZ99999 EMTFEVDPMD EPTLLYLLFE GFDVVRVHQP PSGVIEAVYL RTPFSAGNAT T--

D_65_BAL41721 DMTFEVDPMD EPTLLYLLFE VFDVVRVHQP HRGVIEAVYL RTPFSAGNAT T--

D_62_AEL78842 DMTFEVDPMD EPTLLYLLFE VFDVVRVHQP HRGVIEAVYL RTPFSAGNAT T--

D_25_ABA00007 DMTFEVDPMD EPTLLYLLFE VFDVVRVHQP HRGVIEAVYL RTPFSAGNAT T--

D_59_AEI91289 DMTFEVDPMD EPTLLYLLFE VFDVVRVHQP HRGVIEAVYL RTPFSAGNAT T--

D_19_ABA00002 DMTFEVDPMD EPTLLYLLFE VFDVVRVHQP HRGVIEAVYL RTPFSAGNAT T--

D_64_ABN10535 DMTFEVDPMD EPTLLYLLFE VFDVVRVHQP HRGVIEAVYL RTPFSAGNAT T--

D_27_ABA00009 DMTFEVDPMD EPTLLYLLFE VFDVVRVHQP HRGVIEAVYL RTPFSAGNAT T--

D_28_ACQ91158 DMTFEVDPMD EPTLLYLLFE VFDVVRVHQP HRGVIEAVYL RTPFSAGNAT T--

D_20_ABA00003 DMTFEVDPMD EPTLLYLLFE VFDVVRVHQP HRGVIEAVYL RTPFSAGNAT T--

D_60_AEK87026 DMTFEVDPMD EPTLLYLLFE VFDVVRVHQP HRGVIEAVYL RTPFSAGNAT T--

D_71_AGT76762 DMTFEVDPMD EPTLLYLLFE VFDVVRVHQP HRGVIEAVYL RTPFSAGNAT T--

D_42_ABA00019 DITFEVDPMD EPTLLYLLFE VFDVVRVHQP HRGVIEAVYL RTPFSAGNAT T--

D_51_ABA00026 DMTFEVDPMD EPTLLYLLFE VFDVVRVHQP HRGVIEAVYL RTPFSAGNAT T--

D_30_ABA00012 DMTFEVDPMD EPTLLYLLFE VFDVVRVHQP HRGVIEAVYL RTPFSAGNAT T--

D_29_BAJ22326 DMTFEVDPMD EPTLLYLLFE VFDVVRVHQP HRGVIEAVYL RTPFSAGNAT T--

D_56_ADM66117 DMTFEVDPMD EPTLLYLLFE VFDVVRVHQP HRGVIEAVYL RTPFSAGNAT T--

D_69_AFK92217 DMTFEVDPMD EPTLLYLLFE VFDVVRVHQP HRGVIEAVYL RTPFSAGNAT T--

D_15_BAJ22290 DMTFEVDPMD EPTLLYLLFE VFDVVRVHQP HRGVIEAVYL RTPFSAGNAT T--

B_35_AAP92351 DMTFEVDPMD EPTLLYLLFE VFDVVRVHQP HRGIIEAVYL RTPFSAGNAT T--

B_11_ACZ06785 DMTFEVDPMD EPTLLYLLFE VFDVVRVHQP HRGIIETVYL RTPFSAGNAT T--

B_55_AIS92536 DMTFEVDPMD EPTLLYLLFE VFDVVRVHQP HRGIIETVYL RTPFSAGNAT T--

B_21_AAG21823 DMTFEVDPMD EPTLLYLLFE VFDVVRVHQP HRGVIEAVYL RTPFSAGNAT T--

B_50_ABA00027 DMTFEVDPMD EPTLLYLLFE VFDVVRVHQP HRGVIEAVYL RTPFSAGNAT T--

B_14_AAZ99996 DMTFEVDPMD EPTLLYLLFE VFDVVRVHQP HRGIIETVYL RTPFSAGNAT T--

B_34_AAW33485 DMTFEVDPMD EPTLLYLLFE VFDVVRVHQP HRGIIEAVYL RTPFSAGNAT T--

B_79_BAW32492 DMTFEVDPMD EPTLLYLLFE VFDVVRVHQP HRGIIEAVYL RTPFSAGNAT T--

B_68_AET87230 DMTFEVDPMD EPTLLYLLFE VFDVVRVHQP HRGVIEAVYL RTPFSAGNAT T--

B_66_AET87148 DMTFEVDPMD EPTLLYLLFE VFDVVRVHQP HRGVIEAVYL RTPFSAGNAT T--

E_4_AAD03660 DMNFEVDPMD ESTLLYVVFE VFDVVRVHQP HRGVIEAVYL RTPFSAGNAT T--

B_16_AAW33444 DMTFEVDPMD EPTLLYLLFE VFDVVRVHQP HRGVIEAVYL RTPFSAGNAT T--

A_61_AEK79922 DMTFEVDPME EPTLLYVLFE VFDVVRIHQP HRGVIEAVYL RTPFSAGNAT T--

A_12_BAG48789 DMTFEVDPMD EPTLLYVLFE VFDVVRIHQP HRGVIEAVYL RTPFSAGNAT T--

A_18_AAZ99994 DMTFEVDPMD EPTLLYVLFE VFDVVRIHQP HRGVIEAVYL RTPFSAGNAT T--

A_31_CAO78638 DMTFEVDPME EPTLLYVLFE VFDVVRIHQP HRGVIEAVYL RTPFSAGNAT T--

G_52_ABK35044 DMTFEVDPMD EPTLLYVLFE VFDVVRIHQP HRGVIEAVYL RTPFSAGNAT T--

F_41_ACH90432 DMTFEVDPMD EPTLLYVLFE VFDVVRIHQP HRGVIEAVYL RTPFSAGNAT T--

F_40_AMQ95234 DMTFEVDPMD EPTLLYVLFE VFDVVRIHQP HRGVIEAVYL RTPFSAGNAT T--
